# *De novo* transcriptome assembly: A comprehensive cross-species comparison of short-read RNA-Seq assemblers

**DOI:** 10.1093/gigascience/giz039

**Published:** 2019-05-11

**Authors:** Martin Hölzer, Manja Marz

**Affiliations:** 1 RNA Bioinformatics and High-Throughput Analysis, Friedrich Schiller University, Leutragraben 1, 07743 Jena, Germany; 2 European Virus Bioinformatics Center, Friedrich Schiller University, Leutragraben 1, 07743 Jena, Germany; 3 FLI Leibniz Institute for Age Research, Beutenbergstraße 11, 07743 Jena, Germany

**Keywords:** transcriptomics, RNA-Seq, assembly, de novo, comparison

## Abstract

**Background:**

In recent years, massively parallel complementary DNA sequencing (RNA sequencing [RNA-Seq]) has emerged as a fast, cost-effective, and robust technology to study entire transcriptomes in various manners. In particular, for non-model organisms and in the absence of an appropriate reference genome, RNA-Seq is used to reconstruct the transcriptome *de novo*. Although the *de novo* transcriptome assembly of non-model organisms has been on the rise recently and new tools are frequently developing, there is still a knowledge gap about which assembly software should be used to build a comprehensive *de novo* assembly.

**Results:**

Here, we present a large-scale comparative study in which 10 *de novo* assembly tools are applied to 9 RNA-Seq data sets spanning different kingdoms of life. Overall, we built >200 single assemblies and evaluated their performance on a combination of 20 biological-based and reference-free metrics. Our study is accompanied by a comprehensive and extensible Electronic Supplement that summarizes all data sets, assembly execution instructions, and evaluation results. Trinity, SPAdes, and Trans-ABySS, followed by Bridger and SOAPdenovo-Trans, generally outperformed the other tools compared. Moreover, we observed species-specific differences in the performance of each assembler. No tool delivered the best results for all data sets.

**Conclusions:**

We recommend a careful choice and normalization of evaluation metrics to select the best assembling results as a critical step in the reconstruction of a comprehensive *de novo* transcriptome assembly.

## Background

In the past decade, the sequencing of entire transcriptomes (RNA sequencing [RNA-Seq]) has established itself as a powerful technique to understand versatile molecular mechanisms and to address various biological questions [1–6]. In particular for non-model organisms and in the absence of a suitable reference genome, RNA-Seq is used to reconstruct and quantify whole transcriptomes [1,4,5]. Thus, RNA-Seq allows the identification of differentially expressed genes, even if there is currently no reference genome available: the short reads, nowadays most commonly produced by Illumina systems, can be assembled into contigs [2,4]. Ideally, each contig corresponds to a certain transcript isoform. A key challenge is the management of the resulting data set, especially if different tools and parameter settings are used for the construction of multiple *de novo* transcriptome assemblies. Even though a reference genome is available, it is still recommended to complement a gene expression study by a *de novo* transcriptome assembly to identify transcripts that have been missed by the genome assembly process or are just not appropriately annotated [2].

At first glance, the transcriptome assembly process seems similar to genome assembly, but actually, there are fundamental differences and various challenges. On the one hand, some transcripts might have a shallow expression level, while others are highly expressed [2,4,6]. Especially in eukaryotes, potentially each locus produces several transcripts (isoforms) due to alternative splicing events [4]. Short reads derived from 1 exon can be part of multiple paths in the assembly graph. Therefore, the graph structure can be ambiguous and the represented isoforms can be challenging to resolve. Furthermore, some transcript variants with a low expression level might be considered to be sequencing errors by various tools and removed from the assembly process [7]. As with genome assembly, repetitive regions are also a major problem for the construction of transcripts [8]. The assembly problem gets even more complicated as the transcriptome varies between different cell types, environmental conditions, and time points. A successful transcriptome assembler should address all of these issues and be able to recover full-length transcripts of different levels of expression.

The *de novo* transcriptome assembly of non-model organisms has been on the rise recently, and new tools are frequently developed. Now there is a knowledge gap: which assembly software and parameter settings should be used to construct a *good* assembly? In addition, there is no consensus about which metrics should be used to evaluate the quality of multiple *de novo* transcriptome assemblies.

In the past decade, several tools have been developed specifically for *de novo* transcriptome assembly [9–17]. Some of them are built on top of already existing genome assembly tools [9,11,18]; others were specially designed for transcriptome assembly [10]. Some tools may fit the needs of eukaryotic transcripts, where alternative splicing has to be considered to construct different isoforms, whereas other tools can handle simpler prokaryotic transcripts. More complicating, different RNA-Seq library preparation protocols result in reads of different kinds: single-end vs paired-end, strand-specific vs not strand-specific, different insertion sizes as well as varying read lengths, and can comprise protein- and/or non-coding transcripts.

Although the evaluation of *de novo* transcriptome assembly tools has already been performed in the past [6,19–26], these studies often rely on limited data sets (e.g., a single species, a single sequencing protocol) or focus only on a subset of all currently available assembly tools.

However, all of these studies agree on one point: currently, there is no optimal assembly tool for all RNA-Seq data sets. Different species, sequencing protocols, and parameter settings necessitate different approaches and adjustments of the underlying algorithms to obtain the best possible results. Merging the contigs of different assembly tools and parameter settings to overcome the different disadvantages of certain assemblers and to combine their advantages seems to be the best way to obtain a comprehensive *de novo* transcriptome assembly [22]. Nevertheless, knowing the advantages and disadvantages of each tool is an essential step in the direction of an automated evaluation and merging algorithm for multiple *de novo* transcriptome assemblies.

**Figure 1:**
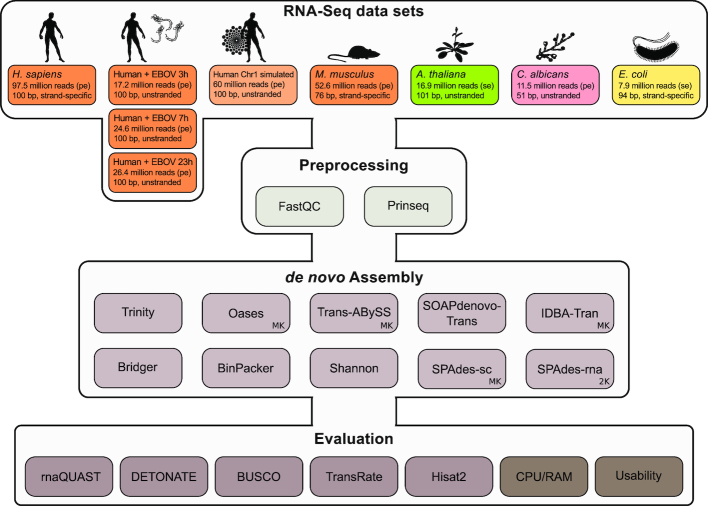
Overview of the RNA-Seq data sets used (orange: eukaryote; light orange: simulated human Chr1; green: plant; pink: fungi; yellow: bacterium) and assembly tools evaluated. Each data set was quality controlled with FastQC [34] and preprocessed with Prinseq [35] before assembly. Overall, >200 single *k*-mer assemblies were calculated. For details about the data sets and assembly tools see Electronic Supplement Tables S1 and S2, respectively. We selected a combination of 20 biological-based and reference-free metrics from the different evaluation tools to assess the quality of each assembly (Table 4 in Methods). The CPU/RAM consumption and the usability of each assembler were not included in the calculated metric scores. Details can be found in the Methods. bp: base pairs; EBOV: Ebola virus; MK: the assembler's built-in multiple–*k*-mer approach was applied; pe: paired-end reads; se: single-end reads. SPAdes-rna uses 2 *k*-mers (2K) per default.

Here, we present a comprehensive evaluation of 10 *de novo* assembly tools (long-standing and novel ones) across 9 short-read RNA-Seq data sets of different species relying on different Illumina sequencing parameters and protocols. In comparison with recent studies, we do not only focus on RNA-Seq data of 1 species or kingdom. Instead, we use data sets from bacteria, fungi, plants, and higher eukaryotes (Fig. 1). We also include data sets from virus-infected cell lines. Our study shows substantial differences between the assembly results of RNA-Seq data derived from various species. We tested promising biological-based and reference-free metrics of several evaluation tools. To evaluate the performance of each assembler, we summarized scores that were normalized in the interval between 0 and 1 of all raw metric values (see Methods). In a next step, such metrics could be used for an automatized selection of good assemblies or contigs to build a more comprehensive and improved cluster-assembly. Our results provide insights into the performance and usability of the different assemblers and how they perform on the different data sets. To our knowledge, this is the most complete comparison of short-read *de novo* transcriptome assembly tools currently available.

## Data Description

### Description of RNA-Seq data used for assembly

We included 9 RNA-Seq data sets of 5 different species with available reference genomes and annotations (Table 1). The data sets cover different kingdoms of life, comprising representatives for bacteria (*Escherichia coli*), fungi (*Candida albicans*), plant (*Arabidopsis thaliana*), and higher eukaryotes (*Mus musculus, Homo sapiens*). The reference genomes, annotations, and coding sequences were obtained from Ensembl (release 87) [27]. For *E. coli* strain K-12 substrain MG1655 and *A. thaliana* reference data was obtained from the Ensembl bacteria [28] or plant [29] database (release 34), respectively. Genome and annotation data for *C. albicans* SC5314 were obtained from the *Candida* Genome Database (Ca22) [30].

**Table 1:** The 9 RNA-Seq data sets used for assembly

No.	Species	ID	Kingdom	Study	Run	Protocol	Reads		Source
							No.	Length (nt)	
1	*E. coli*	ECO	Bacteria	PRJNA238884	SRR1173967	SE, SS	7.9	94	[36]
2	*C. albicans*	CAL	Fungi	PRJNA213618	SRR1654847	PE	11.5	51	[37]
3	*A. thaliana*	ATH	Plant	PRJNA231064	SRR1049376	SE	16.9	101	[38]
4	*M. musculus*	MMU	Animal	PRJNA140057	SRR203276	PE, SS	52.6	76	[10]
5	*H. sapiens*	HSA	Animal	ENCSR000AED		PE, SS	97.5	101	
*H. sapiens*+ EBOV		HSA-EBOV							[31]
6	3 h poi	−3h	Animal + virus	PRJNA429171	SRR6453200	PE	17.2	100	
7	7 h poi	−7h	Animal + virus	PRJNA429171	SRR6453205	PE	24.7	100	
8	23 h poi	−23h	Animal + virus	PRJNA429171	SRR6453206	PE	26.5	100	
Simulated									
9	*H. sapiens* Chr 1	HSA-FLUX	Animal			PE	60.0	100	

Study and run accession numbers are given for the National Center for Biotechnology Information short-read archive (SRA). For the HSA data set the ENCODE data center accession is provided. Read numbers are given in millions. We simulated 1 artificial data set based on protein-coding and non-coding transcripts of human chromosome 1 (Chr 1) using flux simulator [29] (HSA-FLUX). Details can be found in Electronic Supplement Table S1. nt: nucleotides; PE: paired-end reads; SE: single-end reads; SS: strand-specific. *x* h poi indicates total RNA extracted *x* hours post infection.

From a previous study (PRJNA429171) we obtained 3 samples of an Ebola virus (EBOV)-infected HuH7 cell line with total RNA extracted 3 h, 7 h, and 23 h after infection [31] (Table 1). For the evaluation, we concatenated the human genome data with the EBOV genome of strain Zaire, Mayinga (GenBank: NC_002549).

In addition, we quasi-simulated RNA-Seq data based on a selection of protein- and long non-coding transcripts of human chromosome 1 (Chr1). We downloaded the human annotation GTF file and protein-coding sequences (excluding *ab initio* predictions) from Ensembl and selected all protein-coding genes of Chr1 (2,044 genes), comprising 352 genes with 1 isoform, 196 with 2 isoforms, and 1,496 with >2 isoforms. We extended this set of protein-coding genes by 1,075 non-coding genes from Chr1. The combined set of protein- and non-coding genes was used to create a set of transcripts including all known isoforms with a length >200  nucleotides (nt) and without ambiguous N bases from which paired-end reads were simulated. Our final set of transcripts comprised 12,793 protein-coding transcripts as well as 1,006 long intergenic non-coding RNAs, 839 antisense RNAs, and 7 small nucleolar RNAs of human Chr1. Overall 14,645 transcript sequences were used as an input for flux simulator [32] for RNA-Seq raw read simulation, yielding 60 million paired-end 100-nt reads (Table 1). We used flux simulator as suggested for Illumina data, utilizing the default 76–base pair error model. With these simulated sequences, we attempted to mimic a state-of-the-art RNA-Seq data set based on Illumina’s Ribo-Zero protocol for library preparation and ribosomal RNA depletion, further multiplexed 3 times and sequenced on 1 HiSeq 2500 lane.

Details about all RNA-Seq data sets used can be found in Electronic Supplement Table S1 [33].

### Quality control of all RNA-Seq data sets

We investigated the quality of each data set with FastQC [34] and used Prinseq [35] for an initial quality processing of all raw reads. Low-quality regions (with a mean quality <20) were trimmed using a 5-base sliding window approach. Only reads that resulted in a remaining read length of ≥25 nt were considered for further analysis. All reads including ambiguous N bases were removed. PolyA/T tails were trimmed. Details about the trimmed data that were finally used for assembly can be found in Electronic Supplement Table S1.

### Data availability

The RNA-Seq data sets used in our study are publicly available and accessions can be found in the Methods and Supplement Table S1. The processed RNA-Seq data files (FASTQ) as well as all calculated assemblies (FASTA) were uploaded into the Open Science Framework and are freely available under accession doi.org/10.17605/OSF.IO/5ZDX4.

## Analyses

We used 9 RNA-Seq data sets, 10 assembly tools, and various evaluation metrics as summarized in Fig. 1. Details can be found in the Methods and in the comprehensive online Electronic Supplement [33], providing deep insights into the performance of each assembler on each data set and individual metric. With our selection of different data sets, we aimed to represent not only various kingdoms of life but also different experimental setups for RNA-Seq data: (i) single-end vs paired-end data, (ii) strand specificity vs unstranded protocols, (iii) polyA-enriched vs ribosomal RNA–depleted library preparations, (iv) different read lengths, and (v) different sequencing depths.

**Figure 2:**
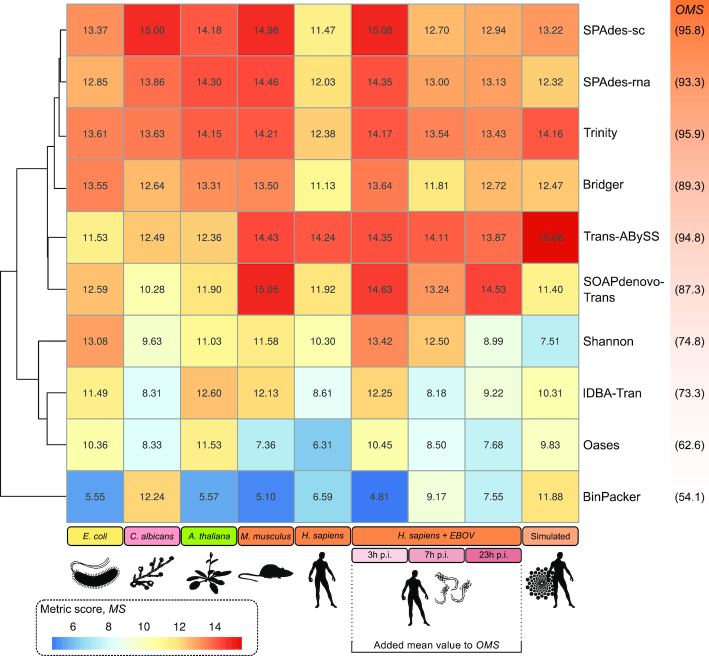
Heat map showing for each data set (column) and each assembler (row) the calculated metric score (MS) (detailed definition in the Methods). The assembly tools are clustered based on their achieved MS over all data sets. The MS for 1 assembly tool and a single data set is based on 20 pre-selected metrics (see Table 4 and Methods for details) and is shown in 1 cell in the heat map (e.g., the MS for *E. coli* and Trinity [10] is 13.61). For each data set, an assembler's MS is the sum of (0,1)-normalized scores of every single metric. The hierarchical clustering of the metric scores divides the assembly tools into 2 groups of generally high-ranked (upper half) and low-ranked (bottom half) tools. Except for Trans-ABySS [9], the MS reached for the largest human RNA-Seq data set is generally lower. Numbers in brackets next to the assembler names present the summarized metric scores (overall metric score, OMS) for all 9 data sets (see Methods). For the 3 similar human data sets infected with EBOV (Fig. 1), we added the mean MS value to the OMS. Details about the metric results for the human data set (no infection) can be found in Table 2 and for all other data sets Electronic Supplement Table S10.

The following sections show how each assembly tool performed for the various data sets and selected evaluation metrics (Table 4 in Methods). For each combination of a metric and a data set, we normalized the achieved raw scores of all assembly tools to range between 0 and 1. This approach is identical to a *z*-score transformation with additional normalization in the interval (0,1) (see Methods for details). In this way, we aimed to achieve the fairest possible comparison of the various data sets, assembly tools, and metrics. For each data set and assembly tool, the normalized scores are summarized to achieve a final score, the so-called "metric score" (MS), for comparison. Table   2 shows the raw and normalized results for all 20 metrics and each assembly tool for the *H. sapiens* data set. Similar tables for all other data sets can be found in Electronic Supplement Table S10. The summarized MS values shown in the last row of Table 2 correspond to the summarized MS values shown for the *H. sapiens* data set in Fig. 2. For example, Trinity [10] achieved an MS of 12.38 for the *H. sapiens* data set across all 20 metrics evaluated (hereafter denoted as 12.38/20) (Fig. 2, Table 2). We further summarized the MS for a single assembly tool overall data sets to calculate an overall metric score (OMS). In the following, the tools sorted by their OMS are discussed in more detail. Further definitions for the calculation of the normalized scores as well as the MS and OMS values are provided in the Methods.

**Table 2:** Results for all 20 selected evaluation metrics

No.	*k*-mer size	Trinity	Oases	Trans-ABySS	SOAP-Trans	Bridger	BinPacker	IDBA-Tran	Shannon	SPAdes-sc	SPAdes-rna
		default	25,35,45,55,65	25,35,45,55,65	default	default	default	25,35,45,55,65	default	default	default
**Evaluation metrics 1–20**											
HISAT2											
1	Overall mapping rate	0.81$_{\, 91.9}$	0.69$_{\, 88.04}$	1.00$_{\, 98.34}$	0.75$_{\, 89.93}$	0.66$_{\, 86.83}$	0.24$_{\, 72.6}$	0.00$_{\, 64.61}$	0.58$_{\, 84.27}$	0.81$_{\, 92.04}$	0.93$_{\, 95.95}$
rnaQUAST											
2	Transcripts ≥1 ,000 nt	0.22$_{\, 64061}$	1.00$_{\, 207474}$	0.20$_{\, 59779}$	0.03$_{\, 27529}$	0.11$_{\, 43201}$	0.00$_{\, 22611}$	0.00$_{\, 23516}$	0.05$_{\, 31328}$	0.05$_{\, 31039}$	0.15$_{\, 49860}$
3	Misassemblies	0.99$_{\, 3378}$	0.00$_{\, 216127}$	0.99$_{\, 2743}$	1.00$_{\, 279}$	0.97$_{\, 7329}$	0.98$_{\, 5603}$	1.00$_{\, 302}$	0.99$_{\, 2837}$	0.99$_{\, 2022}$	0.98$_{\, 5126}$
4	Mismatches per transcript	0.74$_{\, 1.38}$	0.77$_{\, 1.25}$	0.93$_{\, 0.57}$	1.00$_{\, 0.27}$	0.73$_{\, 1.44}$	0.00$_{\, 4.63}$	0.91$_{\, 0.67}$	0.77$_{\, 1.26}$	0.88$_{\, 0.8}$	0.78$_{\, 1.25}$
5	Average alignment length	0.27$_{\, 795.23}$	0.06$_{\, 343.48}$	0.01$_{\, 246.85}$	0.00$_{\, 218}$	0.21$_{\, 654.41}$	1.00$_{\, 2335.73}$	0.13$_{\, 487.11}$	0.23$_{\, 711.83}$	0.09$_{\, 410.22}$	0.09$_{\, 412.24}$
6	95%-assembled isoforms	0.99$_{\, 6788}$	0.10$_{\, 868}$	1.00$_{\, 6824}$	0.31$_{\, 2264}$	0.28$_{\, 2105}$	0.39$_{\, 2824}$	0.07$_{\, 709}$	0.00$_{\, 242}$	0.23$_{\, 1755}$	0.46$_{\, 3253}$
7	Duplication ratio	0.00$_{\, 2.396}$	0.03$_{\, 2.355}$	0.47$_{\, 1.743}$	0.87$_{\, 1.187}$	0.50$_{\, 1.708}$	0.01$_{\, 2.389}$	1.00$_{\, 1.012}$	0.63$_{\, 1.53}$	1.00$_{\, 1.015}$	0.87$_{\, 1.192}$
8	Ex90N50	0.00$_{\, 326}$	0.17$_{\, 666}$	0.06$_{\, 441}$	0.19$_{\, 711}$	0.51$_{\, 1370}$	1.00$_{\, 2381}$	0.19$_{\, 708}$	0.49$_{\, 1324}$	0.42$_{\, 1186}$	0.22$_{\, 782}$
9	No. of full-length transcripts	0.97$_{\, 8930}$	0.83$_{\, 8024}$	1.00$_{\, 9110}$	0.64$_{\, 6806}$	0.89$_{\, 8440}$	0.26$_{\, 4456}$	0.00$_{\, 2783}$	0.63$_{\, 6758}$	0.46$_{\, 5676}$	0.69$_{\, 7155}$
TransRate											
10	Reference coverage	0.87$_{\, 0.23}$	0.33$_{\, 0.09}$	1.00$_{\, 0.26}$	0.34$_{\, 0.09}$	0.31$_{\, 0.09}$	0.27$_{\, 0.07}$	0.31$_{\, 0.08}$	0.00$_{\, 0}$	0.30$_{\, 0.08}$	0.42$_{\, 0.11}$
11	Mean ORF percentage	0.64$_{\, 50.82}$	0.00$_{\, 42.09}$	0.72$_{\, 51.92}$	0.44$_{\, 48.02}$	0.22$_{\, 45.1}$	0.04$_{\, 42.57}$	0.76$_{\, 52.46}$	1.00$_{\, 55.7}$	0.30$_{\, 46.13}$	0.31$_{\, 46.25}$
12	Optimal score^a^	0.30$_{\, 0.13}$	0.00$_{\, 0.02}$	0.23$_{\, 0.11}$	0.66$_{\, 0.27}$	0.32$_{\, 0.14}$	0.14$_{\, 0.07}$	0.61$_{\, 0.25}$	0.13$_{\, 0.07}$	1.00$_{\, 0.4}$	0.57$_{\, 0.23}$
13	Percentage bases uncovered^a^	0.38$_{\, 0.59}$	0.00$_{\, 0.94}$	0.33$_{\, 0.63}$	0.67$_{\, 0.33}$	0.57$_{\, 0.42}$	0.11$_{\, 0.84}$	1.00$_{\, 0.02}$	0.48$_{\, 0.5}$	0.99$_{\, 0.03}$	0.79$_{\, 0.21}$
14	No. of ambiguous bases	0.72$_{\, 286}$	0.00$_{\, 843}$	0.53$_{\, 437}$	0.78$_{\, 241}$	0.83$_{\, 206}$	1.00$_{\, 72}$	0.91$_{\, 138}$	0.94$_{\, 117}$	0.86$_{\, 177}$	0.71$_{\, 294}$
DETONATE											
15	Nucleotide F1	0.59$_{\, 0.43}$	0.08$_{\, 0.18}$	0.77$_{\, 0.51}$	0.89$_{\, 0.57}$	0.71$_{\, 0.48}$	0.00$_{\, 0.15}$	0.86$_{\, 0.55}$	0.42$_{\, 0.35}$	0.97$_{\, 0.61}$	1.00$_{\, 0.62}$
16	Contig F1	0.08$_{\, 0.02}$	0.09$_{\, 0.02}$	0.99$_{\, 0.2}$	1.00$_{\, 0.21}$	0.05$_{\, 0.01}$	0.00$_{\, 0}$	0.08$_{\, 0.02}$	0.11$_{\, 0.02}$	0.07$_{\, 0.01}$	0.06$_{\, 0.01}$
17	KC score	0.87$_{\, 0.51}$	0.00$_{\, 0.24}$	1.00$_{\, 0.55}$	0.42$_{\, 0.37}$	0.51$_{\, 0.4}$	0.40$_{\, 0.37}$	0.14$_{\, 0.29}$	0.58$_{\, 0.42}$	0.47$_{\, 0.39}$	0.60$_{\, 0.43}$
18	RSEM EVAL	0.98$_{\, -6.51}$	0.45$_{\, -11.82}$	1.00$_{\, -6.26}$	0.72$_{\, -9.03}$	0.85$_{\, -7.72}$	0.62$_{\, -10.03}$	0.00$_{\, -16.3}$	0.73$_{\, -8.96}$	0.42$_{\, -12.12}$	0.91$_{\, -7.16}$
BUSCO											
19	Complete BUSCOs	0.96$_{\, 4004}$	0.79$_{\, 3588}$	1.00$_{\, 4106}$	0.39$_{\, 2625}$	0.92$_{\, 3909}$	0.13$_{\, 2009}$	0.00$_{\, 1682}$	0.70$_{\, 3385}$	0.39$_{\, 2625}$	0.58$_{\, 3089}$
20	Missing BUSCOs	0.99$_{\, 1804}$	0.93$_{\, 1922}$	1.00$_{\, 1770}$	0.83$_{\, 2164}$	0.98$_{\, 1812}$	0.00$_{\, 4078}$	0.63$_{\, 2615}$	0.84$_{\, 2133}$	0.78$_{\, 2268}$	0.92$_{\, 1949}$
**Summarized metric (0,1)-score**		12.38	6.31	14.24	11.92	11.13	6.59	8.61	10.3	11.47	12.03

Here, we show results for all 20 selected metrics (rows) based on the output of rnaQUAST [39], HISAT2 [40], DETONATE [41], TransRate [42], BUSCO [43,44], and the Trinity [10] toolkit utilities for the transcripts assembled by all 10 assembly tools (columns). Results are shown for the non-infected *H. sapiens* RNA-Seq strand-specific paired-end library with read length 101 nt (accession No. ENCSR000AED). For each metric normalized scores in the range between 0 and 1 are displayed. The raw values are given in subscript next to the normalized values. In the last row, the summarized MS of (0,1)-normalized scores is given (see Methods for details). The RSEM-EVAL score is divided by 10^9^. The number of ambiguous bases is given in millions. Ex90N50 values are computed as usual N50 but limited to the top most highly expressed transcripts that represent 90% of the total normalized expression data. An F1 score of 1 states that all nucleotides/contigs in the estimated true assembly were recovered with ≥90% identity. KC score: *k*-mer compression score reflecting the similarity of each assembly to DETONATE's estimated “true” assembly. Complete BUSCOs: sum of single-copy and duplicated benchmarked universal single-copy orthologs (BUSCOs). Details and more statistics complementing this evaluation can be found in the Electronic Supplement, Fig. S4–Table S9. Summaries for all other data sets can be found in Table S10. ORF: open reading frame.

^*a*^Not available for the *E. coli* and *A. thaliana* data sets because this metric is only calculated by TransRate in the case of paired-end data.

### Assembly tool performance is diverse regarding different data sets and quality metrics

All evaluated assembly tools are summarized in Fig. 1 and Table 3. Finding the best parameter setting for each tool and each data set is obviously beyond the scope of this evaluation. Therefore, we used the default settings of each tool and adjusted only a few key parameters such as *k*-mer values and strand-specificity options (see Methods for details). Full execution details and commands can be found in the Electronic Supplement, Files S3. For the tools with built-in functions for the automatic integration of different *k*-mer values (Oases, Trans-ABySS, IDBA-Tran, SPAdes; see Table 3), we applied a set of selected *k*-mers (for details see Files S3). If strand-specific data were used for the assembly, we applied the corresponding option of each tool. In application, one should try several different parameter settings and compare the resulting assemblies to optimize the whole assembly process. In particular, different *k*-mers should be tested and evaluated against each other [21]. Here, we carefully chose *k*-mer values to obtain a somewhat fair comparison between the assemblers, although some parameters may not be optimal.

**Table 3:** Overview of the different *de novo* assembly tools evaluated in this study

Assembler	Version	MK	Setup	Usage	Runtime			Memory (GB)			Source	Year
					Min	Max	Median	Min	Max	Median		
Trans-ABySS	2.0.1	Yes	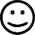	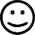	16 m	2 d 6 h 23 m	11 h 11 m	0.6	49.2	19.7	[9]	2010
Trinity	2.8.4	No	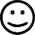	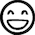	28 m	1 d 20 h 10 m	6 h 40 m	7.2	243.9	27.7	[10]	2011
Oases^*a*^	0.2.08	Yes	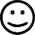	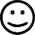	25 m	8 d 15 h 45 m	6 h 47 m	3.1	110.2	31.3	[11]	2012
SPAdes-sc ^*b*^	3.13.0	Yes	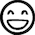	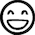	16 m	7 h 52 m	2 h 26 m	5.0	37.4	25.3	[18]	2012
SPAdes-rna ^*b*^	3.13.0	Yes^*c*^	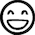	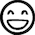	11 m	7 h 24 m	2 h 17 m	5.0	44.2	19.5	[17]	2018
IDBA-Tran	1.1.1	Yes	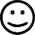	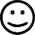	7 m	8 h 49 m	2 h 44 m	0.6	29.1	9.6	[12]	2013
SOAPdenovo-Trans	1.03	No	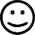	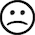	1 m	1 h 48 m	24 m	2.1	45.6	26.4	[13]	2014
Bridger *^d^*	14-12-01	No	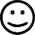	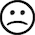	11 m	21 h 11 m	5 h 9 m	1.6	109.3	30.4	[14]	2015
BinPacker ^*d*^	1.0	No	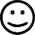	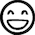	5 m	15 h 57 m	3 h 3 m	1.5	96.2	27.9	[15]	2016
Shannon	0.0.2	No	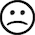	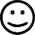	9 m	10 h 45 m	3 h 18 m	3.8	121.4	83.6	[16]	2016

We rated our experiences regarding the installation and usability of each tool (^
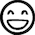
^: excellent; ^
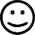
^: good; ^
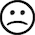
^: unsatisfactory). These experiences might be subjective; nevertheless, we want to share them to give non-experienced users an idea of how difficult it is to get each tool installed (Setup) and executed (Usage) (see Methods for details). For Trinity, we observed high memory peaks at the beginning of the calculations for large (human, mouse) data sets, which immediately returned to moderate memory levels after a few minutes. More details about runtime and memory consumption can be found in Electronic Supplement Fig. S11. MK: presence of a built-in multiple *k*-mer approach and the ability to automatically integrate the output of different *k*-mer runs.

^*a*^ Oases was used on top of the *de novo* genome assembler Velvet (v1.2.10) [45].

^*b*^ SPAdes, originally designed as a *de novo* genome assembler for single-cell data, was used in single-cell modus (–sc) and RNA-Seq modus (–rna).

*^c^*When running SPAdes in RNA-Seq modus, 2 *k*-mer values are used by default.

^*d*^Bridger and BinPacker are based on a splicing graph construction instead of de *Bruijn* graphs.

Whenever a tool was complicated to install (e.g., due to missing dependencies) or could not be run on a specific data set, we attempted to debug the source code and in some cases also contacted the authors to solve the problem. Therefore, we also decided to share our experiences regarding the installation procedure and execution of each tool (Table 3).

#### Trinity

**Figure 3:**
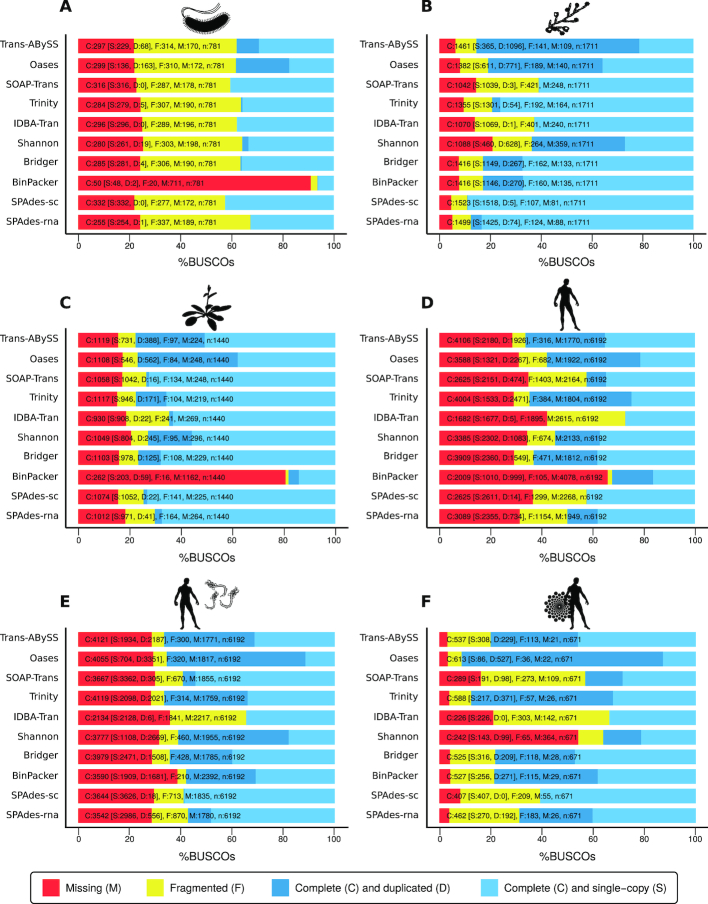
Selected BUSCO (benchmarked universal single-copy orthologs) [43,42] assessment results for *E. coli***(A)**, *C. albicans***(B)**, *A. thaliana***(C)**, *H. sapiens***(D)**, HuH7 cells infected with EBOV 7 h post-infection **(E)**, and flux- simulated reads [29] of human Chr1 **(F)**. The numbers indicate the absolute amount of complete (C) and single-copy (S), complete and duplicated (D), fragmented (F), and missing (M) BUSCOs (see Methods for details). For our evaluation, we have used the number of missing BUSCOs and the combined number of complete/single-copy and complete/duplicated BUSCOs to consider alternative transcripts better. BUSCO results for all other data sets can be found in the Electronic Supplement, Fig. S8.

The re-mapping rate of Trinity [10] was generally high (>90.0%, 97.32% for *C. albicans*) except for the *E. coli* data set (77.01%); see Fig. S4. Trinity performed in the midfield or better regarding the TransRate [44] metrics and very well regarding DETONATEs [41] RSEM-EVAL scores on almost all data sets (Table S6 and S9). Trinity achieved the best RSEM-EVAL scores for 3 of the 9 data sets. The assembler detected many complete BUSCOs [43,44] (Fig. 3) and achieved high 95%-assembled isoform rates [43] for almost all data sets. For the eukaryotic data sets, approximately the half amount of complete BUSCOs is included multiple times in the assembly. This might be a result of the sub-graphs that Trinity relies on to detect different isoforms of 1 transcript [10]. Trinity achieved the best OMS of 95.9 (see Methods for definition) of all assembly tools tested (Fig. 2) and performed generally well in constructing full-length transcripts and the entire Ebola RNA genome out of the virus-infected data sets.

#### SPAdes-sc and -rna

Although initially designed for single-cell and smaller bacterial-sized genome assemblies, we also included SPAdes [18] in our evaluation. It has previously been reported that, when used in single-cell mode, the assembler achieves good results with RNA-Seq data [17,39]. This may be due to the uneven coverage optimization implemented for single-cell data, which also fits very well with the behavior of low- and high-level expressed transcripts. Based on these observations, SPAdes also has a special RNA-Seq mode [17]. Therefore, we evaluated the performance of SPAdes in single-cell (-sc; SPAdes-sc) and transcriptome (-rna; SPAdes-rna) mode (Files S3) and present here the results of both parameter options together.

The re-mapping rates for both SPAdes parameter options were on a comparable level and among the top mapping rates for all data sets (88.04–97.51%; Fig. S4). Based on the TransRate metrics, SPAdes built the most accurate assemblies (Table S6), especially in the single-cell mode. For almost all data sets, the SPAdes-sc and -rna assemblies achieved the highest optimal score, the lowest percentage of uncovered bases, and a low to moderate amount of ambiguous bases together with Trinity, SOAPdenovo-Trans [13], and IDBA-Tran [12]. The RSEM-EVAL scores of the SPAdes assemblies were always good but varied among the different RNA-Seq data sets. For some samples, SPAdes-sc achieved a better score than SPAdes-rna, and vice versa (Table S9). SPAdes assemblies were among the top scorers in complete BUSCO detections, with the -sc mode performing in most cases better than the -rna mode (Fig. 3). Most likely as a result of using only 2 *k*-mers in -rna mode, SPAdes-rna assembled fewer BUSCOs for some data sets (Fig. S8). SPAdes-sc and -rna were the best performing tools for the detection of complete BUSCOs in the *C. albicans* transcriptome (Fig. 3). The SPAdes assemblies generally showed a low duplication ratio (Table S10).

SPAdes-sc achieved one of the top OMSs (OMS = 95.8; Fig. 2), only slightly outperformed by Trinity (OMS = 95.9), and reached the highest MS for the *C. albicans* (MS = 15.0) and the HSA-EBOV–3h (MS = 15.08) assemblies. Comparable to SPAdes in single-cell mode, SPAdes-rna generally performed well on all data sets (OMS = 93.3). Regarding the number of 95%-assembled isoforms, the -rna mode of SPAdes outperformed the single-cell mode for most data sets (Fig. S5). Especially, for larger RNA-Seq data sets, SPAdes-rna was able to reconstruct more full-length transcripts (Table S10). Based on these observations, we suggest that the RNA mode of SPAdes should be preferred for the reconstruction of larger eukaryotic RNA-Seq data sets.

Our comparisons with an older version of SPAdes running in RNA mode (at that time only 1 *k*-mer was allowed) revealed that the performance of the algorithm was greatly improved by using 2 *k*-mers as it is now implemented in the current version [17].

#### Trans-ABySS

Compared with the other tools, Trans-ABySS [9] achieved the highest re-mapping rates (98.45% for *C. albicans*, 99.56% for the simulated data; Fig. S4) but scored only within the midfield or worse regarding the optimal score calculated by TransRate. On the other hand, the assemblies produced by Trans-ABySS achieved for 6 of the 9 data sets the best RSEM-EVAL scores. Only Trinity slightly outperformed Trans-ABySS on this metric for 3 data sets (Table S9). Therefore, the transcripts constructed by Trans-ABySS are well supported by the reads used to build the assembly. Trans-ABySS performed well in all BUSCO analyses and showed a high amount of complete (C) ortholog detections (Fig. 3, Fig. S8). Many hits occurred multiple times (complete and duplicated), e.g., in the *C. albicans* assembly (Fig. S8). This might be a result of the multiple *k*-mer approach (MK), when too many potential isoforms are assembled and not merged accurately at the end of the assembly process. Thus, the assemblies of Trans-ABySS generally showed a high duplication rate (Fig. S5). We observed similar results for the MK runs of Oases [11]. Regarding the amount of fragmented (F) and missing (M) BUSCOs, Trans-ABySS was among the best performing tools (Fig. 3). Trans-ABySS achieved one of the highest OMSs of 94.8 of all assembly tools (Fig. 2) and performed best for the large (human, mouse) data sets and the simulated data of human Chr1. By far, Trans-ABySS achieved the best MS (14.24) for the non-infected human data set. The lowest metric score was achieved for the bacterium data set (Fig. 2). Apart from the running time (Table 3), these results make Trans-ABySS one of the best-performing assembly tools in our comparison (besides Trinity and SPAdes).

#### Bridger

In general, Bridger [14] assemblies resulted in high re-mapping rates between 87.35% (*E. coli*) and 96.72% (*C. albicans*; Fig. S4). For almost all TransRate metrics, the Bridger assemblies placed in the midfield of scores (Table S6). According to the RSEM-EVAL scores, Bridger generally performed well among the top tools (Table S9). Furthermore, Bridger performed well in the detection of complete BUSCOs with a moderate amount of duplicated hits. The amount of missing BUSCOs was comparably low (Fig. 3, Fig. S8). Based on a low duplication ratio and a low number of contigs, Bridger seems to produce very compact but also complete assemblies, especially for smaller data sets. The rate of mismatches per transcript was generally low (Table S10). Altogether, Bridger assemblies were of good quality and achieved among the top scores (OMS = 89.3).

#### SOAPdenovo-Trans

The re-mapping rate of SOAPdenovo-Trans [13] was generally high (>85%), except for the *E. coli* data set (Fig. S4). SOAPdenovo-Trans performed quite well regarding most TransRate statistics and the calculated optimal score (Table S6). In most cases, only the Trinity and SPAdes assemblies could outperform SOAPdenovo-Trans on the TransRate metrics. The RSEM-EVAL scores varied depending on the assembled RNA-Seq data set (Table S9). For the HSA-EBOV–23h and *M. musculus* samples, SOAPdenovo-Trans achieved good RSEM-EVAL scores, whereas for the bacterial, fungal, plant, and the simulated RNA-Seq data the tool placed among the last 3 assemblers. The amount of complete and duplicated BUSCOs was very low (Fig. 3), which correlates with the generally low amount of detected isoforms (e.g., compare number of 95%-assembled isoforms calculated with rnaQUAST; Fig. S5). This could be a result of the single *k*-mer approach. SOAPdenovo-Trans achieved a good OMS of 87.3 (Fig. 2), and the assembler performed well on each evaluated data set (MS between 10.28 and 15.05). SOAPdenovo-Trans was the only assembly tool capable of reconstructing the entire Ebola genome in a single contig from all 3 virus-infected data sets.

#### Shannon

The most variable re-mapping rates were observed for Shannon [16], ranging between 30.77% for the human simulated data set and 96.51% for *A. thaliana* (Fig. S4). The Shannon assemblies did not result in good TransRate optimal scores; however, the percentage of uncovered bases placed in the midfield of all scores and Shannon did not introduce that many ambiguous bases in the assembled transcriptome (Table S6). The RSEM-EVAL scores of Shannon varied among the assembled data sets (Table S9). Regarding the amount of assembled complete BUSCOs, Shannon placed in the midfield and showed a relatively high amount of duplicated hits (Fig. 3). Shannon achieved a moderate OMS of 74.8 (Fig. 2).

#### IDBA-Tran

In general, IDBA-Tran [12] achieved low re-mapping rates between 34.31% (*E. coli*) and 89.04% (*A. thaliana*) (Fig. S4). However, the TransRate metrics of the IDBA-Tran assemblies were generally good (Table S6). Comparable to SOAPdenovo-Trans, some of the IDBA-Tran results were within the top 3 assemblies regarding the optimal score calculated by TransRate. DETONATE's RSEM-EVAL scores revealed a different picture, as IDBA-Tran in many cases placed last in this metric and never reached the top 5 (Table S9). Furthermore, IDBA-Tran was one of the tools with the fewest complete BUSCOs and a high amount of fragmented and missing BUSCOs (Fig. 3 and Fig. S8). The number of 95%-assembled isoforms was generally low (Table S10). IDBA-Tran placed in the lower half of OMS (OMS = 73.3; Fig. 2) and showed the best performance for smaller RNA-Seq data sets.

#### Oases

The re-mapping rates of Oases [11] were generally good (>85%). However, they decreased for the simulated human data (73.26%), the HSA-EBOV–23h data (70.05%), and the *E. coli* data (49.16%) below acceptable thresholds (Fig. S4). Oases introduced the greatest number of ambiguous bases in the assemblies and scored among the last places regarding the TransRate statistics (Table S6). Oases assemblies placed in the bottom third on the RSEM-EVAL scores calculated by DETONATE. However, a good amount of complete BUSCOs could be detected, but many duplicate hits were included, which could be again a result of the MK approach (Fig. 3). In addition, the Oases assemblies comprise an enormous number of contigs (as well as high duplication rates) and introduced many misassemblies (Fig. S5). Oases performed best for the plant, bacteria, and simulated data and achieved an OMS of only 62.6 (Fig. 2).

#### BinPacker

The re-mapping rates of BinPacker [15] were generally low and varied considerably between data sets (36.6–96.7%; Fig. S4). The TransRate metrics of the BinPacker assemblies were comparable to the Bridger results, placing BinPacker among the lower performing tools regarding this statistic (Table S6). On the other hand, BinPacker introduced only a low amount of ambiguous bases in the assemblies. The RSEM-EVAL score was comparatively low, except for the simulated human data, where BinPacker achieved scores similar to Bridger and reached third place behind Trinity and Trans-ABySS (Table S9). Regarding the detection of orthologs, BinPacker had the lowest performance of all tools and was only able to assemble a reasonable amount of complete BUSCOs for *C. albicans*, HSA-EBOV–7h, and the human simulated data set (Fig. 3 and Fig. S8). BinPacker built the smallest assemblies in terms of the number of contigs (Fig. S5). Interestingly, BinPacker achieved for most data sets (and especially for the large human data sets) the best Ex90N50 values (Table S7). Therefore, it seems that BinPacker can construct highly expressed transcripts into long contigs very well. However, the general statistics, and for example, the BUSCO results, show that BinPacker misses many transcripts that might be of low expression in the data sets. Overall, the performance of BinPacker was quite low (OMS = 54.1; Fig. 2) and surprisingly far away from the performance of Bridger (OMS = 89.3), although the assembler is built on the same principles and as an extension of Bridger [15]. In summary, BinPacker showed quite different behavior concerning the MS values, which were generally low, between 5.1 ( *M. musculus*) and 12.24 (*C. albicans*) (Fig. 2).

When designing this study, we also aimed to include an assembly tool that is not based on *k*-mers. Mira [46] (version 4.0rc5) uses an overlap consensus graph for assembly and can be executed in EST mode for RNA-Seq data. However, for 1 human sample 62 h runtime was needed, >300 GB temporary files were produced, and ∼130 GB RAM consumed. Furthermore, we were not able to detect any BUSCO hits in the Mira assemblies. As a result of this low performance and high running time and memory consumption, we decided to remove the tool from our comparison.

### Usability

We rated our experiences regarding the installation and usability of each tool (Table 3). These experiences may be subjective, but we share them to give inexperienced users an idea of how difficult it is to install and run each tool. Some of the tools rely on many dependencies and/or are more difficult to compile (Shannon, SOAPdenovo-Trans, Trans-ABySS), at least on our test system without administrative permissions, while others could be installed easily (SPAdes). Furthermore, some assemblers need additional parameter files for execution (SOAPdenovo-Trans), are circuitous to run (Oases, SOAPdenovo-Trans), need additional preprocessing steps to be performed on the reads (IDBA-Tran assumes paired-end reads to be in order forward–reverse), or are just not terminating for all data sets (Bridger), while with others we had no problems and could execute them straightforwardly (Trinity, SPAdes, BinPacker).

Bridger failed in the path search step for some of the generated temporary files. Therefore, we performed the last step of Bridger by manually combining the transcript output. Furthermore, we had to start Bridger 2 times for each data set because the tool crashed each time after the first start but continued with the assembly when started a second time on the same output folder (see execution commands in Files S3).

In the past, Oases and Trans-ABySS were always circuitous to run because the corresponding genome assemblers Velvet [45] and ABySS [47] needed to be executed first with an MK approach. These difficulties have been somehow overcome by new wrapper scripts provided by the developers to automatically execute the underlying genome assemblers.

### Computational efficiency

Because *de novo* transcriptome assembly can involve the analysis of large sequencing data, computational efficiency is an important benchmark, especially for deep sequencing projects and large sample sizes. Furthermore, it is highly recommended to run multiple assemblies with different tools and parameter settings (e.g., different *k*-mers), so computation time is an important parameter to measure for each tool. Table 3 summarizes the computational time and the memory consumption of all data sets and assemblers. Details can be found in Electronic Supplement Fig. S11.

#### Runtime

By far, SOAPdenovo-Trans proved to be the fastest algorithm, with a median runtime of only 24 m, followed by SPAdes-rna (2 h 17 m), SPAdes-sc (2 h 26 m), IDBA-Tran (2 h 44 m), BinPacker (3 h 3 m), and Shannon (3 h 18 m) (Table 3, Fig. S11). Older tools such as Oases (6 h 47 m) and Trans-ABySS (11 h 11 m), which are additionally based on an MK strategy, are comparatively slower. For example, Oases needed >8 days for the large human RNA-Seq data set. However, if these tools were to be executed only on 1 *k*-mer, the runtime would be comparable to that of the other assemblers or even faster. SOAPdenovo-Trans can also run on different *k*-mers, but no automatic merge function for the different assemblies is implemented. The Trinity median runtime (6 h 40 m) lies between the faster tools and the slower MK approaches, although the tool relies on 1 *k*-mer only. Although based on an MK strategy, IDBA-Tran and SPAdes are much faster than the older MK algorithms and can compete against the other single-*k*-mer tools based on speed.

#### Memory consumption

IDBA-Tran seemed to be the tool with the least memory consumption estimated overall data sets (median, 9.6 GB and maximum, 29.1 GB; Table   3, Fig. S11). Shannon showed high memory peaks (median, 83.6 GB), especially for the larger data sets (>100 GB for the EBOV-infected human samples; see Fig. S11), followed by Oases (31.3 GB), Bridger (30.4 GB), and BinPacker (27.9 GB).

When running Trinity (median memory consumption, 27.7 GB), we observed in the first phase of assembly (meaning in the first seconds up to a few minutes, depending on the size of the input data set) very high memory peaks, especially for the larger data sets. For example, in the first 5 minutes of execution of all human data sets we noticed memory peaks of ∼240 GB with Trinity. Immediately after this initial peak, the memory consumption decreased to comparatively normal levels (Fig. S11). In Electronic Supplement Figure S11, we removed the high initial memory peaks observed for Trinity from the comparison to achieve a better overview of the memory usage of all assemblers. The high memory consumption in the first phase might be a result of the many individual *de Bruijn* graphs built by Trinity based on partitions of the sequence data [10].

Users should pay particular attention to planning enough processing power and time when using many tools for different parameter settings, especially when working on projects with high sequencing depth and large sample size.

### Contamination of viruses decreases performance of most assembly tools

Although not the main focus of this study, we were interested in how the assemblers work with RNA-Seq data as virus contamination increases, and whether they are still able to construct complete viral genomes. Therefore, we used Blastn [48] to search for contigs in the virus-infected assemblies (Fig. 1) that match the full EBOV genome. The EBOV genome comprises a single-stranded RNA genome with negative orientation and a size of ∼19 kb [49]. We assembled 3 human samples infected with EBOV at 3 different time points. Therefore, we were able to investigate how the different assemblers perform on increasing amounts of viral reads in the data (3 h: ∼0.1% viral reads; 7 h: ∼2%; 23 h: ∼20%; compare [31]).

Surprisingly, the performance of most assembly tools in constructing the viral RNA genome decreased with a higher amount of viral reads. In general, Trans-ABySS, SOAPdenovo-Trans, Trinity, Shannon, Bridger, BinPacker, and SPAdes (-sc and -rna mode) performed well and constructed the full EBOV genome out of the 3 h data set. On the 7 h data set (∼2% viral reads), Trinity and SOAPdenovoTrans performed best. Trans-ABySS assembled 2 contigs (9.2 and 9.7 kb) that together would represent the entire EBOV genome. Bridger and BinPacker were only able to construct the same 10-kb partial EBOV genome. SPAdes-rna assembled a partial viral contig of a length of 16 kb. After 23 h post-infection and a viral read contamination of almost 20%, only SOAPdenovo-Trans was able to construct the full EBOV genome with high accuracy (18,901 nt [99.53%]). Bridger, BinPacker, and Trans-ABySS constructed partial virus genomes of a length of 14.8, 12.0, and 10.6 kb, respectively. Trinity built 2 contigs of similar length that together would cover the entire viral genome.

## Discussion

Although the evaluation of *de novo* transcriptome assemblies was frequently performed in the past [6,19–24,26], there is still a lack of knowledge regarding which assembler should be used for which kind of RNA-Seq data. Furthermore, these studies rely on limited data sets (e.g., a single species, a single sequencing protocol) or focus only on a subset of all currently available assembly tools. Here, we present a comprehensive evaluation of 10 *de novo* assembly tools across various RNA-Seq data sets of different kingdoms of life.

### Using a combination of biological-based and reference-free metrics to evaluate an assembly

We evaluated biological/reference-based metrics and statistical/reference-free metrics only based on the input read data and the final assembly itself. Evaluation metrics are important to assess the quality of a genome or transcriptome assembly. However, there is a lack of consensus regarding which evaluation metrics work best for *de novo* transcriptome assembly.

For example, Rana et al. [50] compared different assemblers and *k*-mer strategies using killifish RNA-Seq data and based their comparisons on 11 selected metrics, such as contig number, N50 value, contigs >1 kb, re-mapping rate, number of full-length transcripts, number of open reading frames, DETONATEs RSEM-EVAL score, and the percentage of alignments to closely related fish. Another study performed comparisons on peanut RNA-Seq data and evaluated the assemblies on metrics such as N50, average contig length, number of contigs, and the number of full-length transcripts [51]. Moreton et al. [52] also used the N50 length, the number of transcripts, the number of transcripts ≥1 kb, and reads mapped back to transcripts and CEGMA (Core Eukaryotic Genes Mapping Approach) percentages when evaluating different assemblies of duck. Surely, more information on which metrics best predict the quality of a *de novo* transcriptome assembly would help to establish “best practice” protocols that could be further utilized to develop automatic evaluations to improve assemblies.

There is still a general lack of consensus regarding which metrics should be used for an appropriate evaluation of *de novo* transcriptome assemblies. More complicating, we observed that some metrics provide results that contradict each other, such as the optimal assembly score calculated by TransRate [42] and the RSEM-EVAL score of DETONATE [40]. For example, assemblies of the *H. sapiens* simulated data set achieved the best RSEM-EVAL scores for Trans-ABySS and Trinity, whereas Shannon and IDBA-Tran performed worst (Table S9 and S10). However, IDBA-Tran achieved the second-best optimal score of TransRate, only outperformed by SPAdes-sc, and Shannon scored in next-to-last place on this metric (Tables S6 and S10). On the other hand, certain metrics can be highly correlated (Fig. S12) and therefore lead to further distortions in assembly evaluation.

We conclude that a careful selection of biological-based and reference-free evaluation metrics is necessary to select the best performing results out of multiple assembly runs. In addition, the normalization and the way the results of different metrics are summarized can influence the evaluation. On the basis of our observations, we suggest initially using reference-free metrics as provided by the TransRate [42] software. In general, TransRate’s optimal assembly score seems to be a good measure of the quality of an assembly. Assemblies that needed fewer contigs for a comprehensive description of the whole transcriptome also achieved in most cases good TransRate scores (Table S6). However, this score can be calculated only for paired-end RNA-Seq data at the moment.

If biological/reference-based metrics should be included, the 95%-assembled isoforms statistics calculated by rnaQUAST [39], as well as the scores calculated by BUSCO [43,42] and the number of fully reconstructed protein-coding transcripts, are good metrics for the evaluation of the best assembly results.

### Different species and RNA-Seq setups require specialized assembly tools

Although no tool's performance was dominant for all data sets, we found that Trinity [10], SPAdes [17,18], and Trans-ABySS [9] produced consistently good assemblies among all data sets, followed by Bridger [14] and SOAPdenovo-Trans [13] (Fig. 2).

SPAdes, although originally developed as a *de novo* assembly tool for small genomes, also produced highly accurate transcriptome assemblies in both modes, for single-cell (SPAdes-sc) and RNA-Seq data (SPAdes-rna). Interestingly, the single-cell mode outperformed the RNA mode for some of the data sets on our metrics (Fig. 2). This might be a result of the 2 *k*-mer approach and the different handling of single-end data in the RNA mode. According to the authors [17], SPAdes-rna was initially designed based on the principles of SPAdes-sc so that an MK option could be easily activated as well. However, it was noticed that smaller *k*-mers result in a higher number of false junctions and lead to more misassemblies for transcriptomic data. Therefore, the authors decided only to use 2 *k*-mers as the default in RNA mode [17]. Furthermore, to join sequences with small overlaps, SPAdes-rna uses a gap-closing procedure based on read pairs [17]. Indeed, this might be one reason why SPAdes-rna achieved for some metrics lower scores for single-end data. Taking a closer look at the BUSCO results, SPAdes produced in both modes the fewest complete and duplicated transcripts (Fig. 3). This could further indicate that SPAdes merges highly similar transcripts into single contigs, therefore losing similar isoforms. This behavior can also be observed when looking at the number of 95%-assembled isoforms calculated with rnaQUAST (Fig. S5 and Table S10). Here, the single-cell mode of SPAdes scored for most data sets in the midfield whereas in RNA mode more complete isoform assemblies are constructed.

On closer examination of the BUSCO (Fig. 3) and fully reconstructed transcript results, Oases [11] performed well overall. However, the tool produced the highest quantities of complete and duplicated hits, which might indicate that highly similar isoforms derived from the MK approach are not resolved efficiently. Oases, as well as Trans-ABySS and SOAPdenovo-Trans, constructed large assemblies with a high number of (sometimes very small) contigs. By far, Oases constructed the highest number of contigs but did not achieve the best reference coverage in all test cases. For example, the Oases assembly of the *H. sapiens* data set comprises ∼207,000 transcripts with a length >1,000 nt, covering only 8% of the reference transcripts (Table 2). In comparison, the Trans-ABySS assembly needed only ∼59,000 contigs with a length >1,000 nt to achieve a reference coverage of 26% (Table S10). Therefore, Oases has the potential to create good assembly results but also produces big assemblies with many contigs that might complicate and confuse downstream analyses.

With a median runtime of only 24 minutes over all data sets (maximum runtime, 1 h 48 min), SOAPdenovo-Trans [13] outperformed all other assemblers (Table 3, Fig. S11). Combined with the moderate memory consumption (median, 26.4 GB; maximum, 45.6 GB), this makes SOAPdenovo-Trans the most resource-efficient tool evaluated in this study. However, it might be interesting to run MK assemblies with SOAPdenovo-Trans and use another assembly merge strategy (e.g., conducted from Oases or TransABySS) to merge the final transcripts resulting from each run. In general, MK approaches (Trans-ABySS, SPAdes, IDBA-Tran, Oases) performed better than single *k*-mer approaches regarding full-length isoform reconstruction and assembly completeness.

As long as the amount of viral contamination in RNA-Seq data was low (∼0.1%), all assembly tools except Oases and IDBA-Tran generated accurate viral contigs with high similarity to the EBOV genome and a length >18 kb. In general, SOAPdenovoTrans performed best on all 3 virus-infected data sets by constructing accurate full-length contigs with high similarity to the EBOV genome. Therefore, it will be interesting to evaluate the performance of SOAPdenovo-Trans for the construction of RNA viral genomes out of meta-transcriptomic RNA-Seq data in the future.

## Potential implications

Here, we present a large-scale comparative study by applying 10 *de novo* assembly tools to 9 RNA-Seq data sets comprising different kingdoms of life (Fig. 1). Overall, we calculated >200 single assemblies and evaluated their performance on different metrics (Table 4). All results are summarized in a comprehensive Electronic Supplement, which is easily extendable by more RNA-Seq data sets, new assembler versions, parameter settings, and tools. We summarize some key findings from our comparative study:

- No tool's performance was dominant for all data sets. However, Trinity, SPAdes, and Trans-ABySS, followed by Bridger and SOAPdenovo-Trans, were among the best assembly tools (Fig. 2).
- SOAPdenovo-Trans followed by Trinity performed best for the construction of the EBOV single-stranded RNA genome at all 3 time points tested.
- SOAPdenovo-Trans had the lowest runtime, followed by SPAdes, IDBA-Tran, Shannon, and BinPacker.
- For assembly evaluation, we recommend a hybrid approach by combining biological-based (e.g., BUSCO [43,42], the number of full-length transcripts) and reference-free metrics (e.g., TransRate [42], DETONATE [41]).

**Table 4:** Selected evaluation metrics applied for each assembly and data set

No.	Tool	Selected metric	Source
1	HISAT2	Overall mapping rate	[40]
2	rnaQUAST	Transcripts ≥1,000 nt	[39]
3*		Misassemblies	
4*		Mismatches per transcript	
5*		Average alignment length	
6*		95%-assembled isoforms	
7*		Duplication ratio	
8	Trinity/Salmon	Ex90N50^*a*^	[10,53]
9*	Trinity/Blastx	Full-length transcripts^*b*^	[10,48]
10*	TransRate	Reference coverage	[42]
11		Mean ORF percentage	
12		Optimal score^*c*^	
13		Percentage bases uncovered^*c*^	
14		Number of ambiguous bases	
15	DETONATE	Nucleotide F1	[41]
16		Contig F1	
17		KC score	
18		RSEM-EVAL	
19*	BUSCO	Complete BUSCOs^*d*^	[43,42]
20*		Missing BUSCOs	

Metrics marked with an asterisk are biological/reference-based. All other metrics only rely on the reads used to build the assembly and/or the resulting contigs. Details can be found in the Methods. ORF: open reading frame.

^*a*^N50 statistic limited to the most highly expressed transcripts, which account for 90% of the total normalized expression data, calculated with the Trinity toolkit utilities.

^*b*^Number of proteins covered by >90% by assembled transcripts.

^*c*^Not available for the *E. coli* and *A. thaliana* data sets because only calculated by TransRate if paired-end data are available.

^*d*^Sum of complete single-copy and complete duplicated BUSCOs.

In general, assembly tools such as Trinity, SPAdes, and Trans-ABySS, which are still well maintained, outperformed other tools and should be preferred.

Some of our metrics might not provide independent assessment metrics, such as the number of complete BUSCOs and the number of full-length transcripts (see Fig. S12). To account for such bias between highly correlated metrics, each of our (0,1)-normalized scoring vectors (see Methods) could be multiplied with a weight value (e.g., 0.5). Because it is a somewhat arbitrary decision how to set the weight value for each metric and because we have also observed differences between the data sets, we have decided not to adjust weights in this comparison. However, we only chose 1 of several metrics if our results suggested a strong correlation. In addition, our overall results do not appear to be strongly influenced by such metric correlations on the basis of our internal comparisons. Future assembly evaluation tools may allow the user to define weights for specific metrics or could calculate different weights automatically based on other statistics. Another possibility could be to bundle potentially correlated metrics on the basis of very similar normalized evaluation vectors.

Furthermore, our current comparison does not accurately test how well the individual assemblers reconstruct alternatively spliced transcripts. Therefore, another metric could be included to consider the assembler’s ability to reconstruct different isoforms as an important aspect of a comprehensive evaluation of transcriptome assemblies.

### Limitations and future work

We still recommend applying different tools and parameter settings for *de novo* transcriptome assembly, followed by the evaluation of the output transcripts and selecting the best-performing results. This general idea needs to be investigated in more detail in future studies. The selection of the best assemblies based on appropriate metrics and the subsequent clustering process (without loss of isoforms and the additional introduction of greater redundancy) remain challenging and open tasks.

#### Dynamic extension of this comparison

A common problem of many comparative studies is that they can only make limited proposals based on the tools and data sets available at the time they were conducted. The Electronic Supplement provided here remains consistent with the presented results but can be extended with other metrics, data sets, and assembly tools in future updates.

#### Cluster assembly

Furthermore, the complementary performance of the top-performing tools motivated the development of an ensemble method by combining the best performing methods to achieve an overall better assembly. Based on our findings, a pipeline should be developed that automatically selects the top-performing assemblies (or only the best transcripts from each assembly) using a hybrid approach of biological-based and reference-free metrics and clusters them on the basis of sequence similarity and read coverage to achieve a more comprehensive assembly. For the large bioinformatics community working in the area of RNA-Seq, the development of a high-performing (accurate and fast) *de novo* transcriptome cluster workflow to automatically select and combine the output of top-performing assembly tools remains a challenging however crucial task.

## Methods

### Description of assembly tools and executed commands

We collected 10 *de novo* assembly tools for the transcriptome reconstruction of the 9 RNA-Seq data sets (Table 1), summarized in Table 3 and Electronic Supplement Table S2.

Six of these transcriptome assemblers are specially designed for working with RNA-Seq data and are based on *de Bruijn* graphs: Trans-ABySS (RRID:SCR_013322) [9], Trinity (RRID:SCR_013048) [10], Oases (RRID:SCR_011896) [11], IDBA-Tran (RRID:SCR_011891) [12], SOAPdenovo-Trans (RRID:SCR_013268) [13], and Shannon [16].

Trans-ABySS and Oases are built on top of the *de novo* genome assemblers ABySS v2.1.1 (RRID:SCR_010709) [47] and Velvet v1.2.10 (RRID:SCR_010755) [45], respectively. Both support MK values by running the underlying genome assembler multiple times and merging the assembled contigs. We executed Trans-ABySS (v2.0.1) and Oases (v0.2.08) with MK and in strand-specific mode, if suitable (Files S3).

Trinity and SOAPdenovo-Trans (the latter one built on the principles of SOAPdenovo2 [RRID:SCR_014986] [54]) are stand-alone *de novo* transcriptome assembly tools, also based on *de Bruijn* graphs but lacking an automated MK support. Whereas for SOAPdenovo-Trans different single *k*-mer values can be applied, Trinity relies on a fixed *k*-mer value of 25. Trinity (v2.8.4) was run with default parameters and, if suitable, in strand-specific mode (Files S3). For SOAPdenovo-Trans (v1.03), currently no strand-specific assembly is supported [13].

IDBA-Tran (v1.1.1), a novel assembly tool that claims to be more robust regarding uneven expression levels in RNA-Seq data [12], was run with MK and has no option for strand-specific assembly (Files S3). As IDBA-Tran assumes paired-end reads to be in forward-reverse order we manually converted the orientation of reads if necessary.

Shannon (v0.0.2), a so-called information-optimal *de novo* RNA-Seq assembler [16], was used with a single default *k*-mer value and if suitable in strand-specific mode (-ss; Files S3).

We used Bridger [14] (v2014-12-01) and BinPacker [15] (v1.0), 2 assembly tools that rely on splicing graphs [14] instead of *de Bruijn* graphs. Bridger provides a new framework for *de novo* transcriptome assembly that “bridges” between techniques employed in the Cufflinks [55] pipeline and the Trinity tool, in order to overcome the limitations of Trinity. BinPacker was developed on the basis of Bridger's principles and utilizes similar to Shannon coverage information to dissolve corresponding isoforms efficiently. Bridger can only run with single *k*-mer values between 19 and 32 with a default of 25. We executed Bridger with the default *k*-mer and, if possible, with the strand-specific option (-SS_lib_type). However, for 2 strand-specific RNA-Seq data sets (*M. musculus, H. sapiens*) the tool failed and was executed in the default unstranded mode (Files S3). We observed problems with strand-specific paired-end data in this version of Bridger. The strand-specific assembly of the single-end *E. coli* data (-SS_lib_type F) ran without problems. BinPacker was executed on a single *k*-mer value and if suitable in strand-specific (-m F|RF) mode (Files S3).

We further included SPAdes v3.13.0 (RRID:SCR_000131) [18], a widely used *de novo* genome assembler based on *de Bruijn* graphs and MK values. We were interested in determining how well the tool's optimization for single-cell assembly could be applied to RNA-Seq data and how the tool performed in contrast to the aforementioned specialized transcriptome assemblers. Since version 3.9.0 an RNA-Seq mode is also implemented, which uses 2 *k*-mers for assembly if possible [17]. We evaluated the performance of SPAdes in single-cell (-sc; SPAdes-sc) and RNA-Seq (-rna; SPAdes-rna) mode. Henceforth, we refer to SPAdes-sc and SPAdes-rna as 2 different assemblers, although both are based on the same tool.

In total, we calculated >200 single *k*-mer assemblies (Files S3; doi.org/10.17605/OSF.IO/5ZDX4). Each assembler was run on each data set (Fig. 1). If possible, MKs were used (Table 3). Trans-ABySS, Oases, and IDBA-Tran include a built-in functionality for MKs. SPAdes-sc/-rna can automatically choose multiple/2 *k*-mers for the assembly process and were therefore executed with these default options. For the *E. coli, A. thaliana, H. sapiens*, and the artificial data sets *k*-mers 25, 35, 45, 55, and 65 were used with Trans-ABySS, Oases, and IDBA-Tran. *M. musculus* data were assembled with the *k*-mers 25, 35, 45, and 55 because the read length is shorter in comparison to the bacterial and plant data sets. The short-read *C. albicans* data were run with *k*-mers 21, 27, 33, and 39. The EBOV-infected HuH7 samples were run with *k*-mers 25, 29, 33, 37, and 41. All *k*-mer values were selected on the basis of previous results for these data sets and in relation to the different read lengths and sequencing setups. All assemblers were run with default parameters if not otherwise stated. Details about the execution of each tool on each data set can be found in the Electronic Supplement, Files S3.

### Evaluation metrics

We benchmarked all assembly results using various evaluation tools (Fig. 1) from which 20 metrics were selected (summarized in Table 4). Nine metrics are based on reference sequences and annotations, whereas the others are only based on the final assembly itself (the contigs) or the reads that were used to construct the assembly. We also evaluated the computational efficiency (runtime, memory) to assess the applicability of the tools for deeply sequenced data sets and/or large sample size.

#### Mapping rate

We used HISAT2 v2.0.4 (RRID:SCR_015530) [41] to map the quality-controlled reads back to each assembly. The re-mapping rate can give preliminary insights into the quality of a transcriptome assembly (Fig. S4); however, further metrics are needed to assess a more complete picture of each assembler’s performance.

#### Ex90N50

We have used the Trinity [10] toolkit utilities to calculate a modification of the widely used Nx statistic that also takes transcript expression data into account. This so-called expression-informed ExN50 statistic compensates for short and weakly expressed transcripts that can dominate a transcriptome assembly and can drive the N50 value towards small values for high-quality assemblies. Here we refer to the Ex90N50 value, which calculates the N50 statistics as usual but is limited to the most highly expressed transcripts, which account for 90% of the total normalized expression data. We used Salmon [53] (v0.11.3) for fast alignment-free abundance estimation to calculate the Ex90N50 values (Table S7).

#### Reconstruction of full-length protein-coding transcripts

To assess the number of (nearly) full-length reconstructed protein-coding transcripts, we used Blastx (RRID:SCR_001653) [48] against the UniProtKB/Swiss-Prot database (RRID:SCR_004426) [56] followed by scripts provided by the Trinity [10] toolkit utilities. To improve the overall sequence coverage, we first grouped Blast hits of a single transcript aligning to a single protein sequence with several discontinuous alignments for each assembly (Trinity toolkit script: blast_outfmt6_group_segments.pl). Based on the grouped output, we have calculated the distribution of the percentage length coverage for the top matching database entries (blast_outfmt6_group_segments.tophit_coverage.pl). Finally, for each assembly the number of proteins that are covered by >90% of their protein's length by assembled transcripts were reported.

Note that we performed the Blastx search with the parameters -evalue 1e-20 and -max_target_seqs 1. By setting the maximum target sequences to 1, we drastically reduced the runtime but only reported the first hit passing the e-value threshold. Therefore, we did not necessarily report the best match for each transcript. This problem of misinterpretation of the parameter was recently discussed in the bioinformatics community [57]. However, for our comparison the overall results would only change slightly by increasing the maximum number of target sequences.

#### rnaQUAST

We used rnaQUAST [39] (v1.5.1) to calculate various statistics for each assembly and to demonstrate the completeness and correctness levels of the assembled transcripts. The tool was run with reference transcriptomes to calculate the sensitivity and specificity of an assembly. To check for redundancy in the assemblies, we have included the duplication ratio from the sensitivity report as 1 metric. Furthermore, rnaQUAST calculates various bar plots and histograms to visualize basic statistics such as transcript lengths, mismatch rates, and the number of transcript alignments per isoform. All plots and detailed statistics can be found in the Electronic Supplement, Fig. S5.

#### TransRate

TransRate [42] (v1.0.3) examines an assembly and compares it to experimental evidence such as the reads the assembly was built on. One of our metrics relies on the optimal reference-free TransRate score that utilizes only the reads that were used to generate the assembly as evidence (Table 4). Such a metric should be generally better at optimizing the assembly process because the comparison to a reference will always penalize genuine biological novelty contained in the assembly. The score is produced for the whole assembly and every single contig. Currently, the score can be calculated only for paired-end data. The score of an assembly is calculated as the geometric mean of all contig scores multiplied by the proportion of input reads that provide positive support for the assembly [44]. Thus, the score captures how confident one can be in what was assembled, as well as how complete the assembly is. The minimum possible score is 0.0, while 1.0 is the maximum score (Table S6).

#### DETONATE

We further used the DETONATE workflow: a pipeline for the “DE novo TranscriptOme rNa-seq Assembly with or without the Truth Evaluation” [41] (v1.11). We mainly focused on DETONATE's RSEM-EVAL score. This statistically based evaluation score utilizes multiple factors, such as the compactness of the assembly and its support from the RNA-Seq reads. Therefore, the RSEM-EVAL score can be used to evaluate assemblies even when the ground truth is unknown. Assemblies with higher RSEM-EVAL scores are considered better. DETONATE was run for all assemblies as recommended in the online vignette [58]. The main metrics calculated by DETONATE can be found in Electronic Supplement Table S9.

#### BUSCO

We benchmarked universal single-copy orthologs with BUSCO v2.0 (RRID:SCR_015008) [39]. The tool detects orthologous candidate genes in the assemblies and assesses the presence and abundance of single-copy orthologs as an evaluation criterion. The so-called BUSCOs are selected from OrthoDB orthologous groups at major species radiations requiring orthologs to be present as single-copy genes in the vast majority (>90%) of available species. BUSCO provides a quantitative assessment of the completeness of an assembly in terms of expected gene content. The results are further simplified into categories of (i) complete and single-copy, (ii) complete and duplicated, (iii) fragmented, or (iv) missing BUSCOs. For our evaluation, we summed up the amount of complete/single-copy and complete/duplicated BUSCOs to also take into account the different isoforms reconstructed from the assembly tools.

For the evaluation of the simulated human data set, the Euarchontoglires reference data set was reduced to BUSCO orthologs originating only from human Chr1 (671 BUSCOs). The full BUSCO output for each data set can be found in the Electronic Supplement, Fig. S8.

### Calculation of normalized evaluation scores

We investigated the performance of 10 *de novo* assembly tools *a_k_* ∈ {*a*_1_, …, *a*_10_} on 9 RNA-Seq data sets *d_i_* ∈ {*d*_1_, …, *d*_9_} using 20 pre-selected metrics *m_j_* ∈ {*m*_1_, …, *m*_20_}. For each combination of a data set *d_i_* and a metric *m_j_* we define a vector $\mathbf{v}^{i,j}$ of raw scores $r_{k}^{i,j}$ for each assembly tool *a_k_* as

\begin{equation*}
\mathbf{v}^{i,j}=(r_{1}^{i,j},\dots ,r_{10}^{i,j}).
\end{equation*}

Then, we normalized the values of the vector $\mathbf{v}^{i,j}$ to the interval (0,1) using

\begin{equation*}
\mathrm{normalize}(\mathbf{v}_k^{i,j})=\frac{\mathbf{v}_{k}^{{i,j}} - \mathrm{min}(\mathbf{v}^{i,j})}{\mathrm{max}(\mathbf{v}^{i,j}) - \mathrm{min}(\mathbf{v}^{i,j})} = n_k^{i,j}
\end{equation*}

and denoted the resulting vector of (0,1)-normalized scores as

\begin{equation*}
\mathbf{n}^{i,j}=(n_1^{i,j}, \dots , n_{10}^{i,j}).
\end{equation*}

For example, the following vector of raw scores results for the *E. coli* data set *d*_eco_, the metric overall mapping rate*m*_omr_, and the corresponding raw scores of all 10 assembly tools:

\begin{equation*}
\mathbf{v}^{\mathrm{eco,omr}}=(77.0, 49.1, 95.7, 56.6, 87.4, 71.1, 34.3, 76.7, 88.0, 89.0).
\end{equation*}

In this case, the assembly tool *a*_3_ achieved an overall mapping rate of 95.7. After (0,1)-normalization the vector results in the following:

\begin{equation*}
\mathbf{n}^{\mathrm{eco,omr}}=(0.7,0.24, 1.0, 0.36, 0.86, 0.6, 0.0, 0.69, 0.88, 0.89).
\end{equation*}

This normalization of the raw metric values to the interval (0,1) yields the same results as a *z*-score transformation with additional (0,1)-normalization.

We define the metric score MS for an assembly tool *a_k_* and a data set *d_i_* as the sum of all (0,1)-normalized scores $\mathbf{n}_k^{i,j}$ over all 20 pre-selected metrics *m_j_* as

\begin{equation*}
\mathrm{MS}(d_i,a_k)=\sum _{j=1}^{{20}}\mathbf{n}_k^{i,j}.
\end{equation*}

An MS(*d_i_*, *a_k_*) of 14.62 would mean that the assembler *a_k_* for data set *d_i_* achieved a normalized and summarized score of 14.62 from a maximum possible score of 20 (the number of metrics; denoted as 14.62/20).

To get a more general overview of the performance of each assembler, we summed up the metric scores MS an assembler achieved for each data set *d_i_* to calculate an overall metric score (OMS) for each assembler:

\begin{equation*}
\mathrm{OMS}(a_k) = \sum _{i=1}^{9}\mathrm{MS}(d_i,a_k).
\end{equation*}

The 3 human RNA-Seq data sets from specimens exposed to the EBOV and sampled 3, 7, and 23 h post-infection [31] are based on the same sequencing parameters and comprise roughly the same amount of reads (Fig. 1 and Table S1). Owing to this similarity, we decided to reduce the impact of systematic assembly errors when calculating the OMS for 1 assembly tool and used the mean of all 3 MS scores for these 3 data sets (Fig.   2). For example, Trans-ABySS [9] performed very well in constructing the human transcripts out of all 3 Ebola-infected data sets regarding the MS (14.35/20, 14.11/20, and 13.87/20), whereas BinPacker [15] did not (4.81/20, 9.17/20, and 7.55/20); see Fig. 2.

The maximum achievable metric score for the *E. coli* and *A. thaliana* data sets is 18 and not 20, because the optimal score and the percentage of uncovered bases are only calculated by TransRate [42] in the case of paired-end data. The calculated MSs and OMSs are summarized in Fig. 2.

### Computational resources

All calculations were run on 2 symmetric multiprocessing servers with 14 TB storage (raid-5) and 48 CPU cores each, comprising 4 AMD Opteron 6238 CPUs and 512 GB RAM. Each assembly was executed on 48 threads.

### Usability

We further aimed to install and run all tools without root rights on our test system (Debian GNU/Linux 8 [jessie] 64-bit). Of course, how easily a tool can be installed and executed depends heavily on the machine used, the server setup, and how familiar the user is with the programing language the tool is based on. Nevertheless, it should be the goal of each publicly available piece of software to be as user-friendly as possible. Therefore, we collected our experiences during the installation and execution of each assembler to share our observations (Table 3).

## Availability of supporting data and materials

A comprehensive Electronic Supplement publicly available at www.rna.uni-jena.de/supplements/assembly [33] accompanies this study. The electronic supplement will stay consistent with the results presented in this article. Updates, including new assembly tools, versions, and data sets, will be marked and additionally linked on subpages online. In addition, we have uploaded all processed read data, assemblies, blast alignments, mapping files, and the complete electronic supplement as an additional archive into the Open Science Framework under accession doi.org/10.17605/OSF.IO/5ZDX4 [59]. Additional intermediate and final result files for evaluation tools such as BUSCO and TransRate as well as other results are also archived in the *GigaScience* GigaDB respository [60].

## Additional files

**Supplementary Table S1**: Data sets and preprocessing.

**Supplementary Table S2**: Assembly tools.

**Supplementary Files S3**: Executed assembly commands.

**Supplementary Figures S4**: HISAT2 re-mapping rate.

**Supplementary Figures S5**: rnaQUAST statistics.

**Supplementary Tables S6**: TransRate.

**Supplementary Figures S7**: ExN50.

**Supplementary Figures S8**: BUSCO.

**Supplementary Tables S9**: DETONATE.

**Supplementary Tables S10**: Selected main metrics.

**Supplementary Figures S11**: Runtime and memory consumption.

**Supplementary Figures S12**: (0,1)-normalized scores per data set and metric.

GIGA-D-18-00307_Original_Submission.pdf

GIGA-D-18-00307_Revision_1.pdf

GIGA-D-18-00307_Revision_2.pdf

GIGA-D-18-00307_Revision_3.pdf

Response_to_Reviewer_Comments_Original_Submission.pdf

Response_to_Reviewer_Comments_Revision_1.pdf

Response_to_Reviewer_Comments_Revision_2.pdf

Reviewer_1_Report_Original_Submission -- Andrey D. Prjibelski, M.Sc. 9/26/2018 Reviewed

Reviewer_1_Report_Revision_1 -- Andrey D. Prjibelski, M.Sc. 2/7/2019 Reviewed

Reviewer_2_Report_Original_Submission -- Brian Haas 9/27/2018 Reviewed

Reviewer_2_Report_Revision_1 -- Brian Haas 1/27/2019 Reviewed

## Abbreviations

BUSCO: benchmarked universal single-copy orthologs; Chr1: chromosome 1; EBOV: Ebola virus; HSA: *Homo sapiens*; KC: *k*-mer compression; MK: multiple *k*-mer; MS: metric score; nt: nucleotides; OMS: overall metric score; RNA-Seq: RNA sequencing.

## Competing interests

The authors declare that they have no competing interests.

### Funding

This work has been funded by the German Research Foundation (DFG) projects Collaborative Research Center/Transregio 124—“Pathogenic fungi and their human host: Networks of Interaction,” subproject B5; DFG SPP-1596—“Ecology and species barriers in emerging viral diseases”; and CRC 1076 “AquaDiva”, subproject A06.

### Authors’ contributions

M.M. conceived the research idea. M.H. designed the project, performed calculations and analysis, interpreted the data, and wrote the main manuscript. M.M. contributed in discussions and in proofreading the final manuscript. This work is part of the doctoral thesis of M.H. All authors read and approved the final manuscript.
