# Supplementary material for: De novo transcriptome assembly: A comprehensive cross-species comparison of short-read RNA-Seq assemblers
Source: Gigascience. 2019 May 11;8(5):giz039. doi: 10.1093/gigascience/giz039 (PMC6511074; doi:10.1093/gigascience/giz039)
Supplement: GIGA-D-18-00307_Revision_3.pdf [file giz039_giga-d-18-00307_revision_3.pdf]

GigaScience

De novo transcriptome assembly: A comprehensive cross-species comparison of short-read RNA-Seq assemblers

--Manuscript Draft--

**Manuscript Number:**	GIGA-D-18-00307R3
**Full Title:**	De novo transcriptome assembly: A comprehensive cross-species comparison of short-read RNA-Seq assemblers
**Article Type:**	Research
**Funding Information:**	Deutsche Forschungsgemeinschaft (Collaborative Research Center/Transregio 124 - "Pathogenic fungi and their human host: Networks of Interaction", subproject B5) — Dr. Martin Hölzer<br>Deutsche Forschungsgemeinschaft (SPP-1596 - "Ecology and species barriers in emerging viral diseases") — Dr. Martin Hölzer<br>Deutsche Forschungsgemeinschaft (CRC 1076 "AquaDiva", subproject A06) — Dr. Martin Hölzer
**Abstract:**	Background: In recent years, massively parallel cDNA sequencing (RNA-Seq) has emerged as a fast, cost-effective and powerful technology to study entire transcriptomes in various manners. In particular, for non-model organisms and in the absence of an appropriate reference genome, RNA-Seq is used to reconstruct the transcriptome de novo. Although the de novo transcriptome assembly of non-model organism has been on the rise recently and new tools are developed frequently, there is still a knowledge gap about which assembly software should be used to build a comprehensive de novo assembly.<br>Results: Here we present a large-scale comparative study in which ten de novo assembly tools are applied to nine RNA-Seq data sets spanning different kingdoms of life. Overall, we build more than 200 single assemblies and evaluated their performance on a combination of 20 biological-based and reference-free metrics. Our study is accompanied by a comprehensive and extensible Electronic Supplement that summarizes all data sets, assembly execution instructions, and evaluation results. Trinity, SPAdes and Trans-ABySS, followed by Bridger and SOAPdenovo-Trans, generally outperformed the other tools compared. In addition, we observed species-specific differences in the performance of each assembler. No tool delivered the best results for all data sets.<br>Conclusions: We recommend a careful choice and normalization of evaluation metrics to select the best assembling results as a critical step in the reconstruction of a comprehensive de novo transcriptome assembly.
**Corresponding Author:**	Martin Hölzer<br>Friedrich Schiller University Jena<br>Jena, Thüringen GERMANY
**Corresponding Author Secondary Information:**	
**Corresponding Author's Institution:**	Friedrich Schiller University Jena
**Corresponding Author's Secondary Institution:**	
**First Author:**	Martin Hölzer
**First Author Secondary Information:**	
**Order of Authors:**	Martin Hölzer<br>Manja Marz
**Order of Authors Secondary Information:**	
**Response to Reviewers:**	I included the following requested changes:

	1) added a reference [59] for the OSF and cited it in the data availability section<br><br>2) added a reference [60] for GigaDB and wrote in the data availability section:<br>"Additional intermediate and final result files for evaluation tools such as BUSCO and TransRate as well as other results are also archived in the GigaScience GigaDB repository [60]."<br><br>3) added RRIDs for:<br>- Trans-ABySS (RRID:SCR_013322)<br>- Trinity (RRID:SCR_013048)<br>- Oases (RRID:SCR_011896)<br>- IDBA-Tran (RRID:SCR_011891)<br>- SOAPdenovo-Trans (RRID:SCR_013268)<br>- ABySS (RRID:SCR_010709)<br>- Velvet (RRID:SCR_010755)<br>- SOAPdenovo2 (RRID:SCR_014986)<br>- SPAdes (RRID:SCR_000131)<br>- HISAT2 (RRID:SCR_015530)<br>- BLASTX (RRID:SCR_001653)<br>- UniProtKB/Swiss-Prot (RRID:SCR_004426)<br>- BUSCO (RRID:SCR_015008)<br><br>Thank you very much and best wishes,<br>Martin Hoelzer
**Additional Information:**	
**Question**	**Response**
Are you submitting this manuscript to a special series or article collection?	No
**Experimental design and statistics**<br><br>Full details of the experimental design and statistical methods used should be given in the Methods section, as detailed in our Minimum Standards Reporting Checklist. Information essential to interpreting the data presented should be made available in the figure legends.<br><br>Have you included all the information requested in your manuscript?	Yes
**Resources**<br><br>A description of all resources used, including antibodies, cell lines, animals and software tools, with enough information to allow them to be uniquely identified, should be included in the Methods section. Authors are strongly encouraged to cite Research Resource Identifiers (RRIDs) for antibodies, model organisms and tools, where possible.	Yes

Powered by Editorial Manager® and ProduXion Manager® from Aries Systems Corporation

Have you included the information requested as detailed in our Minimum Standards Reporting Checklist?	
**Availability of data and materials**<br><br>All datasets and code on which the conclusions of the paper rely must be either included in your submission or deposited in publicly available repositories (where available and ethically appropriate), referencing such data using a unique identifier in the references and in the “Availability of Data and Materials” section of your manuscript.<br><br>Have you have met the above requirement as detailed in our Minimum Standards Reporting Checklist?	Yes

Click here to access/download;Manuscript;GIGA-D-18-00307.pdf
Click here to view linked References

OXFORD (GIGA)n SCIENCE

GigaScience, 2018, 1–17

doi: xx.xxxx/xxxx
Manuscript in Preparation
Research

RESEARCH

De novo transcriptome assembly: A comprehensive cross-species comparison of short-read RNA-Seq assemblers

Martin Hölzer[1,2,*] and Manja Marz[1,2,3]

[1]RNA Bioinformatics and High-Throughput Analysis, Friedrich Schiller University, Leutragraben 1, Jena, Germany, and [2]European Virus Bioinformatics Center, Friedrich Schiller University, Leutragraben 1, Jena, Germany, and [3]FLI Leibniz Institute for Age Research, Beutenbergstraße 11, Jena, Germany

[*]To whom correspondence should be addressed. Email: martin.hoelzer@uni-jena.de

Abstract

Background: In recent years, massively parallel cDNA sequencing (RNA-Seq) has emerged as a fast, cost-effective and powerful technology to study entire transcriptomes in various manners. In particular, for non-model organisms and in the absence of an appropriate reference genome, RNA-Seq is used to reconstruct the transcriptome *de novo*. Although the *de novo* transcriptome assembly of non-model organism has been on the rise recently and new tools are developed frequently, there is still a knowledge gap about which assembly software should be used to build a comprehensive *de novo* assembly.
Results: Here, we present a large-scale comparative study in which ten *de novo* assembly tools are applied to nine RNA-Seq data sets spanning different kingdoms of life. Overall, we build more than 200 single assemblies and evaluated their performance on a combination of 20 biological-based and reference-free metrics. Our study is accompanied by a comprehensive and extensible Electronic Supplement that summarizes all data sets, assembly execution instructions, and evaluation results. `Trinity`, `SPAdes` and `Trans-ABySS`, followed by `Bridger` and `SOAPdenovo-Trans`, generally outperformed the other tools compared. In addition, we observed species-specific differences in the performance of each assembler. No tool delivered the best results for all data sets.
Conclusions: We recommend a careful choice and normalization of evaluation metrics to select the best assembling results as a critical step in the reconstruction of a comprehensive *de novo* transcriptome assembly.

Key words: transcriptomics, RNA-Seq, assembly, *de novo*, comparison

Background

In the last decade, the sequencing of entire transcriptomes (RNA sequencing, RNA-Seq) has established itself as a powerful technique to understand versatile molecular mechanisms and to address various biological questions [1–6]. In particular for non-model organisms and in the absence of a suitable reference genome, RNA-Seq is used to reconstruct and quantify whole transcriptomes [1, 4, 5]. Thus, RNA-Seq allows the identification of differentially expressed genes, even if there is currently no reference genome available: The short reads, nowadays most commonly produced by Illumina systems, can be assembled into contigs [2, 4]. Ideally, each contig corresponds to a certain transcript isoform. A key challenge is the management of the resulting data set, especially if different tools and parameter settings are used for the construction of multiple *de novo* transcriptome assemblies. Even though a reference genome is available, it is still recommended to complement a gene expression study by a *de novo* transcriptome assembly to identify transcripts that have been missed by the genome assembly process or are just not appropriately annotated [2].

Compiled on: March 7, 2019.
Draft manuscript prepared by the author.

At first glance, the transcriptome assembly process seems similar to genome assembly, but actually there are fundamental differences and various challenges. On the one hand, some transcripts might have a very low expression level, while others are highly expressed [2, 4, 6]. Especially in eukaryotes, potentially each locus produces several transcripts (isoforms) due to alternative splicing events [4]. Short reads derived from one exon can be part of multiple paths in the assembly graph. Therefore, the graph structure can be ambiguous and the represented isoforms can be difficult to resolve. Furthermore, some transcript variants with a low expression level might be considered as sequencing errors by various tools and removed from the assembly process [7]. As with genome assembly, repetitive regions are also a major problem for the construction of transcripts [8]. The assembly problem gets even more complicated as the transcriptome varies between different cell types, environmental conditions, and time points. A successful transcriptome assembler should address all of these issues and be able to recover full-length transcripts of different levels of expression.

The *de novo* transcriptome assembly of non-model organism has been on the rise recently and new tools are developed frequently. Now there is a knowledge gap: Which assembly software and parameter settings should be used to construct a *good* assembly? In addition, there is no consensus about which evaluation metrics should be used to evaluate the quality of multiple *de novo* transcriptome assemblies.

In the last decade, several tools have been developed specifically for *de novo* transcriptome assembly [9–17]. Some of them are build on top of already existing genome assembly tools [9, 11, 18], others were specially designed for transcriptome assembly [10]. Some tools may fit the needs of eukaryotic transcripts, where alternative splicing has to be considered to construct different isoforms, whereas other tools can handle simpler prokaryotic transcripts. More complicating, different RNA-Seq library preparation protocols result in reads of different kinds: single-end vs. paired-end, strand-specific vs. not strand-specific, different insertion sizes as well as varying read lengths and can comprise protein- and/or non-coding transcripts.

Although the evaluation of *de novo* transcriptome assembly tools have been already performed in the past [6, 19–26], these studies often rely on limited data sets (e.g. a single species, a single sequencing protocol) or focus only on a subset of all currently available assembly tools.

Though, all of these studies agree on one point: currently, there is no optimal assembly tool for all RNA-Seq data sets. Different species, sequencing protocols and parameter settings need different approaches and adjustments of the underlying algorithms to obtain the best possible results. Merging the contigs of different assembly tools and parameter settings to overcome the different disadvantages of certain assemblers and to combine their advantages seems to be the best way to obtain a comprehensive *de novo* transcriptome assembly [22]. Nevertheless, knowing the advantages and disadvantages of each tool is an important step in the direction of an automated evaluation and merging algorithm for multiple *de novo* transcriptome assemblies.

Here, we present a comprehensive evaluation of ten *de novo* assembly tools (long-standing and novel ones) across nine short-read RNA-Seq data sets of different species relying on different Illumina sequencing parameters and protocols. In comparison to recent studies, we do not only focus on RNA-Seq data of one species or kingdom. Instead, we use data sets from bacteria, fungi, plants, and higher eukaryotes (Fig. 1). We also include data sets that underwent viral infections. Our study shows substantial differences between the assembly results of RNA-Seq data derived from various species. We tested promising biological-based and reference-free metrics of several evaluation tools. To evaluate the performance of each assembler, we summarized scores that were normalized in the interval between 0 and 1 of all raw metric values (see Methods). In a next step, such metrics could be used for an automized selection of *good* assemblies or contigs to build a more comprehensive and improved *cluster-assembly*. Our results give insights into the performance and usability of the different assemblers and how they perform on the different data sets. As far as our knowledge goes, this is the most complete comparison of short-read *de novo* transcriptome assembly tools currently available.

Data Description

Description of RNA-Seq data used for assembly

We included nine RNA-Seq data sets of five different species with available reference genomes and annotations (Tab. 1). The data sets cover different kingdoms of life, comprising representatives for bacteria (*Escherichia coli*), fungi (*Candida albicans*), plant (*Arabidopsis thaliana*), and higher eukaryotes (*Mus musculus*, *Homo sapiens*). The reference genomes, annotations, and coding sequences were obtained from Ensembl (release 87) [34]. For *E. coli* str. K-12 substr. MG1655 and *A. thaliana* reference data was obtained from the Ensembl bacteria [35] or plant [36] database (release 34), respectively. Genome and annotation data for *C. albicans* SC5314 were obtained from the *Candida* Genome Database (Ca22) [37].

From a previous study (PRJNA429171) we obtained three samples of an Ebola virus (EBOV) infected HuH7 cell line with total RNA extracted 3 h, 7 h, and 23 h post infection [33] (Tab. 1). For the evaluation, we concatenated the human genome data with the EBOV genome of strain Zaire, Mayinga (GenBank: NC_002549).

In addition, we quasi-simulated RNA-Seq data based on a selection of protein- and long non-coding transcripts of human chromosome 1 (chr1). We downloaded the human annotation GTF file and protein-coding sequences (excluding *ab initio* predictions) from Ensembl and selected all protein-coding genes of chr1 (2,044 genes), comprising 352 genes with one isoform, 196 with two isoforms and 1,496 with more than two isoforms. We extended this set of protein-coding genes by 1,075 non-coding genes from chr1. The combined set of protein- and non-coding genes was used to create a set of transcripts including all known isoforms with a length >200 nt and without ambiguous `N` bases from which paired-end reads were simulated. Our final set of transcripts comprised 12,793 protein-coding transcripts as well as 1,006 lincRNAs, 839 antisense RNAs, and 7 snoRNAs of human chr1. Overall 14,645 transcript sequences were used as an input for `flux simulator` [29] for RNA-Seq raw read simulation, yielding 60 million paired-end 100 nt reads (Tab. 1). We used `flux simulator` as suggested for Illumina data, utilizing the default 76-bp error model. With this simulated sequences, we attempt to mimic a state-of-the-art RNA-Seq data set based on Illumina's Ribo-Zero protocol for library preparation and rRNA depletion, further multiplexed three times and sequenced on one HiSeq 2500 lane.

Details about all used RNA-Seq data sets can be found in Electronic Supplement Tab. S1 [38].

Quality control of all RNA-Seq data sets

We investigated the quality of each data set with `FastQC` [27] and used `Prinseq` [28] for an initial quality processing of all raw reads. Low-quality regions were trimmed with an average

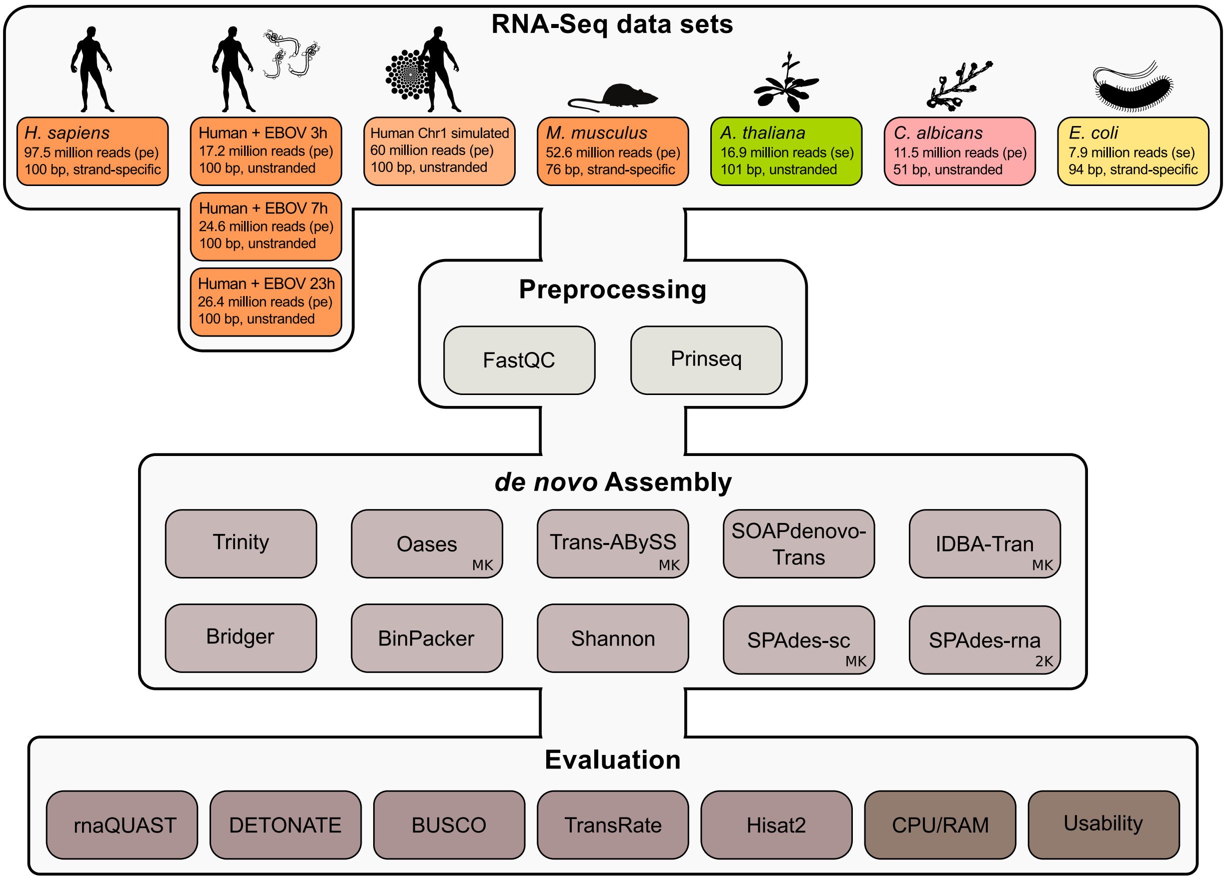

Figure 1. Overview of the used RNA-Seq data sets (orange – eukaryote, light orange – simulated human chromosome 1, green – plant, pink – fungi, yellow – bacterium) and evaluated assembly tools. Each data set was quality controlled with `FastQC` [27] and preprocessed with `Prinseq` [28] prior to assembly. Overall, more than 200 single k-mer assemblies were calculated. For details about the used data sets and assembly tools see Electronic Supplement Tab. S1 and Tab. S2, respectively. We selected a combination of 20 biological-based and reference-free metrics from the different evaluation tools to assess the quality of each assembly (Tab. 4 in Methods). The CPU/RAM consumption and the usability of each assembler were not included in the calculated metric scores. Details can be found in the Methods. EBOV – Ebola virus; se/pe – single-end/paired-end; MK – the assemblers built-in multiple-k-mer approach was applied. `SPAdes-rna` uses two k-mers (2K) per default.

quality below 20 using a five base sliding window approach. Only reads that have resulted in a remaining read length of at least 25 nt were considered for further analysis. All reads including ambiguous `N` bases were removed. PolyA/T tails were trimmed. Details about the trimmed data, finally used for assembly, can be found in Electronic Supplement Tab. S1.

Data availability

The RNA-Seq data sets used in our study are publicly available and accessions can be found in the Methods and online Tab. S1. The processed RNA-Seq data files (`FASTQ`) as well as all calculated assemblies (`FASTA`) were uploaded into the Open Science Framework and are freely available under accession doi.org/10.17605/OSF.IO/5ZDX4.

Analyses

We used nine RNA-Seq data sets, ten assembly tools, and various evaluation metrics as summarized in Fig. 1. Details can be found in the Methods and in the comprehensive online Electronic Supplement [38], providing deep insights into the performance of each assembler on each data set and individual metric. With our selection of different data sets, we aim to represent not only various kingdoms of life, but also different experimental setups for RNA-Seq data: 1) single-end vs. paired-end data, 2) strand specificity vs. unstranded protocols, 3) polyA enriched vs. rRNA depleted library preparations, 4) different read lengths, and 5) different sequencing depths.

The following sections show how each assembly tool performed for the various data sets and selected evaluation metrics (Tab. 4 in Methods). For each combination of a metric and a data set, we normalized the achieved raw scores of all assembly tools to range between 0 and 1. This approach is identical to a z-score transformation with additional normalization in the interval [0,1] (see Methods for details). In this way we want to achieve the fairest possible comparison of the various data sets, assembly tools and metrics. For each data set and assembly tool the normalized scores are summarized to achieve a final score, the so-called *metric score* (*MS*), for comparison. Tab. 2 shows the raw and normalized results for all 20 metrics and each assembly tool for the *H. sapiens* (HSA) data set. Similar tables for all other data sets can be found in Electronic Supplement Tab. S10. The summarized *metric scores* shown in the last row of Tab. 2 correspond to the summarized *MS* values shown for the HSA data set in Fig. 2. For example, `Trinity` [10] achieved an *MS* of 12.38 for the *H. sapiens* data set across all 20 metrics evaluated and hereafter denoted as 12.38/20 (Fig. 2, Tab. 2). We further summarized the *MS* for a single assembly tool over all data sets to calculate an *overall metric score* (*OMS*). In the following, the tools sorted by their *OMS* are discussed in more detail. Further definitions for the calculation of the normalized scores as well as the *MS* and *OMS* values are contained in the Methods.

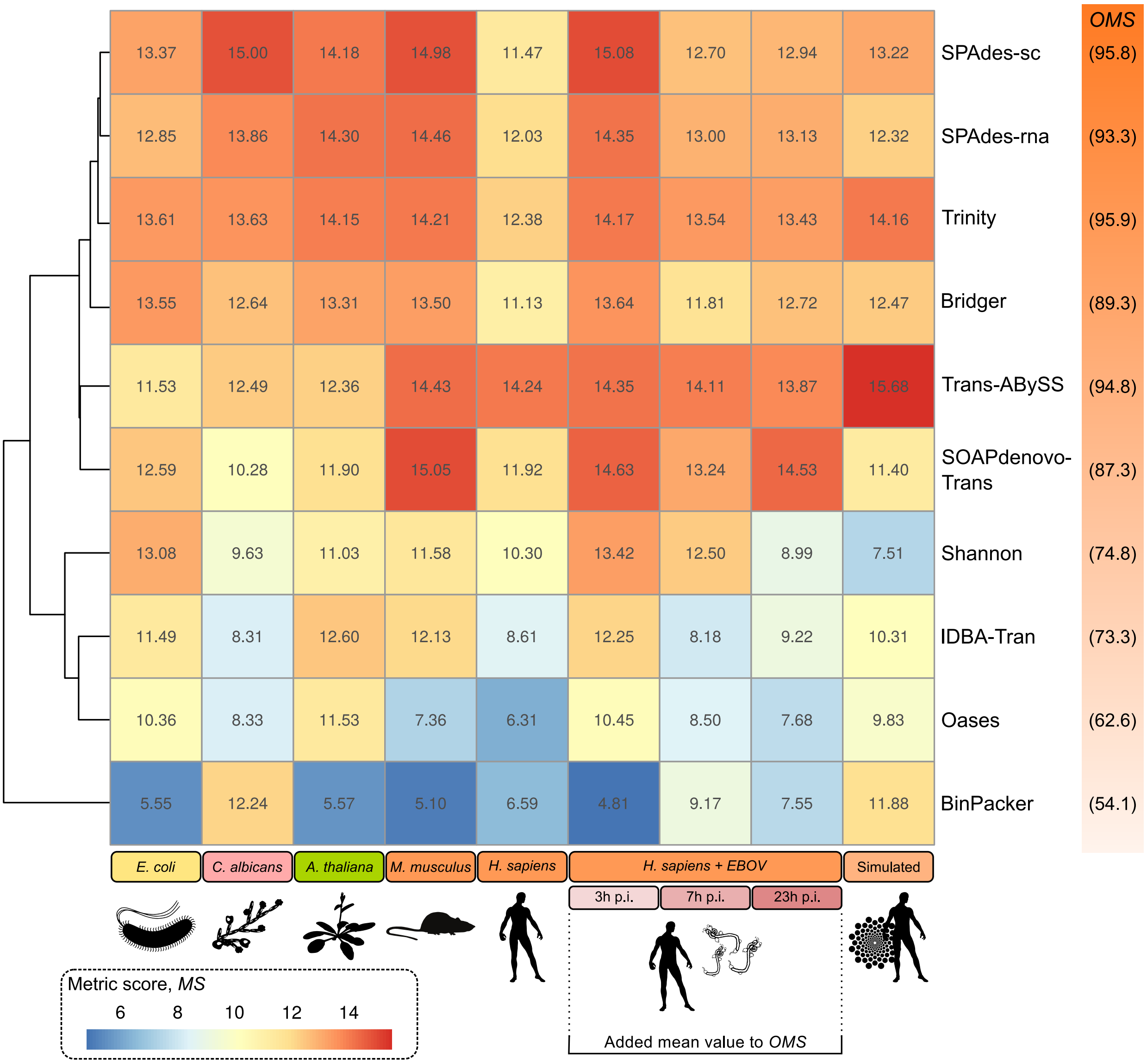

Figure 2. Heat map showing for each data set (column) and each assembler (row) the calculated *metric score MS* (detailed definition in the Methods). The assembly tools are clustered based on their achieved *MS* over all data sets. The *MS* for one assembly tool and a single data set is based on 20 pre-selected metrics (see Tab. 4 and Methods for details) and is shown in one cell in the heat map (e.g. the *MS* for *E. coli* and `Trinity` [10] is 13.61). For each data set, an assemblers *MS* is the sum of (0,1)-normalized scores of each single metric. The hierarchical clustering of the metric scores divides the assembly tools in two groups of generally high-ranked (upper half) and low-ranked (bottom half) tools. With the exception of `Trans-ABySS` [9], the *MS* reached for the largest human RNA-Seq data set is generally lower. Numbers in brackets next to the assembler names present the summarized metric scores (*overall metric score*, *OMS*) for all nine data sets (see Methods). For the three similar human data sets infected with the Ebola virus (Fig. 1), we added the mean *MS* value to the *OMS*. Details about the metric results for the human data set (HSA; no infection) can be found in Tab. 2 and for all other data sets in Electronic Supplement Tab. S10.

Table 1. Nine RNA-Seq data sets were used for assembly. Study and run accession numbers are given for the NCBI short-read archive (SRA). For the HSA data set the ENCODE data center accession is provided. Read numbers are given in million. We simulated one artificial data set based on protein-coding and non-coding transcripts of human chromosome 1 (Chr 1) using `flux simulator` [29] (HSA-FLUX). Details can be found in Electronic Supplement Tab. S1. se/pe – single-end/paired-end; ss – strand-specfic. EBOV – Ebola virus; *x* h poi – total RNA extracted *x* hours post infection.

Nr.	Species	Id.	Kingdom	Study	Run	Protocol	Reads		Ref.
							num.	length	
1	*Escherichia coli*	ECO	Bacteria	PRJNA238884	SRR1173967	se, ss	7,9	94 nt	[30]
2	*Candida albicans*	CAL	Fungi	PRJNA213618	SRR1654847	pe	11,5	51 nt	[31]
3	*Arabidopsis thaliana*	ATH	Plant	PRJNA231064	SRR1049376	se	16,9	101 nt	[32]
4	*Mus musculus*	MMU	Mammal	PRJNA140057	SRR203276	pe, ss	52,6	76 nt	[10]
5	*Homo sapiens*	HSA	Mammal	ENCSR000AED	–	pe, ss	97,5	101 nt	–
Homo sapiens + EBOV		HSA-EBOV							[33]
6	*3 h poi*	-3h	Mammal+Virus	PRJNA429171	SRR6453200	pe	17,2	100 nt	
7	*7 h poi*	-7h	Mammal+Virus	PRJNA429171	SRR6453205	pe	24,7	100 nt	
8	*23 h poi*	-23h	Mammal+Virus	PRJNA429171	SRR6453206	pe	26,5	100 nt	
Simulated									
9	*Homo sapiens* Chr 1	HSA-FLUX	Mammal	–	–	pe	60,0	100 nt	–

Assembly tools performing diverse regarding different data sets and quality metrics

All evaluated assembly tools are summarized in Fig. 1 and Tab. 3. Finding the best parameter setting for each tool and each data set is obviously beyond the scope of this evaluation. Therefore, we used the default settings of each tool and adjusted only a few key parameters such as *k*-mer values and strand-specificity (see Methods for details). Full execution details and commands can be found in the Electronic Supplement, Files S3. For the tools with built-in functions for the automatic integration of different *k*-mer values (`Oases`, `Trans-ABySS`, `IDBA-Tran`, `SPAdes`; see Tab. 3), we applied a set of selected *k*-mers (for details see Files S3). If strand-specific data was used for the assembly, we applied the corresponding option of each tool. In application, one should try several different parameter settings and compare the resulting assemblies to optimize the whole assembly process. In particular, different *k*-mers should be tested and evaluated against each other [21]. Here, we carefully chose *k*-mer values to obtain a somewhat fair comparison between the assemblers, although some parameters may not be optimal.

Whenever a tool was difficult to install (e.g. due to missing dependencies) or could not be run on a specific data set, we attempted to debug the source code and in some cases also contacted the authors to solve the problem. Therefore, we also decided to share our experiences regarding the installation procedure and execution of each tool (Tab. 3).

Trinity.

The re-mapping rate of `Trinity` [10] was generally high (above 90.0 %, 97.32 % for *C. albicans*) except for the *E. coli* data set (77.01 %), see Fig. S4. `Trinity` performed in the midfield or better regarding the `TransRate` [42] metrics and very well regarding `DETONATES` [41]) RSEM-EVAL scores on almost all data sets (Tab. S6 and S9). `Trinity` achieved the best RSEM-EVAL scores for three of the nine data sets. The assembler detected many complete BUSCOs [43, 44] (Fig. 3) and achieved high 95 %-*assembled isoform* rates [39] for almost all data sets. For the eukaryotic data sets, approximately the half amount of complete BUSCOs is included multiple times in the assembly. This might be a result of the sub-graphs `Trinity` relies on to detect different isoforms of one transcript [10]. `Trinity` achieved the best *OMS* of 95.9 (see Methods for definition) of all assembly tools tested (Fig. 2) and performed generally good in constructing full-length transcripts and the entire Ebola RNA genome out of the virus-infected data sets.

SPAdes-sc and -rna.

Although initially designed for single-cell and smaller bacterial-sized genome assemblies, we also included `SPAdes` [18] in our evaluation. It has previously been reported that, when used in single-cell mode, the assembler achieves good results with RNA-Seq data [17, 39]. This may be due to the uneven coverage optimization implemented for single-cell data, which also fits very well with the behavior of low and high-level expressed transcripts. Based on this observations, `SPAdes` also has a special RNA-Seq mode [17]. Therefore, we evaluated the performance of `SPAdes` in single-cell (`--sc`; `SPAdes-sc`) and transcriptome (`--rna`; `SPAdes-rna`) mode (Files S3) and present here the results of both parameter options together.

The re-mapping rates for both `SPAdes` parameter options are on a comparable level and arrange among the top mapping rates for all data sets (88.04–97.51 %, Fig. S4). Based on the `TransRate` metrics, `SPAdes` build the most accurate assemblies (Tab. S6), especially in the single-cell mode. For almost all data sets, the `SPAdes-sc` and `-rna` assemblies achieved the highest *optimal score*, the lowest *percentage of uncovered bases*, and a low up to a moderate *amount of ambiguous bases* together with `Trinity`, `SOAPdenovo-Trans` [13], and `IDBA-Tran` [12]. The RSEM-EVAL scores of the `SPAdes` assemblies are always good but vary among the different RNA-Seq data sets. For some samples, `SPAdes-sc` achieves a better scoring than `SPAdes-rna`, and vice versa (Tab. S9). `SPAdes` assemblies arrange in the top scores of complete BUSCO detections, with the `--sc` mode performing in most cases better than the `--rna` mode (Fig. 3). Most likely due to only two *k*-mers used in `--rna` mode, `SPAdes-rna` assembled a lower amount of BUSCOs for some data sets (Fig. S8). `SPAdes-sc` and `-rna` are the best performing tools for the detection of complete BUSCOs in the *C. albicans* transcriptome (Fig. 3). The `SPAdes` assemblies generally show a low duplication ratio (Tab. S10).

`SPAdes-sc` achieved one of the top places regarding our summarized metric score (*OMS*=95.8, Fig. 2), only slightly outperformed by `Trinity` (*OMS*=95.9), and reached the highest metric scores for the *C. albicans* (*MS*=15.0) and the HSA-EBOV-3h (*MS*=15.08) assemblies. Comparable to `SPAdes` in single-cell mode, `SPAdes-rna` performed generally good on all data sets (*OMS*=93.3). Regarding the number of 95 %-assembled isoforms, the `-rna` mode of `SPAdes` outperformed the single-cell mode for most data sets (Fig. S5). Especially, for larger RNA-Seq data sets, `SPAdes-rna` was able to reconstruct more full-

Table 2. Here, we show results for all 20 selected metrics (rows) based on the output of rnaQUAST [39], HISAT2 [40], DETONATE [41], TransRate [42], BUSCO [43, 44], and the Trinity [10] toolkit utilities for the transcripts assembled by all ten assembly tools (columns). Results are shown for the non-infected *H. sapiens* RNA-Seq strand-specific paired-end library with read length 101 nt (accession number ENCSR000AED). For each metric normalized scores in the range between 0 and 1 are displayed. The raw values are given in subscript next to the normalized values. In the last row, the summarized *metric score* (*MS*) of (0,1)-normalized scores is given (see Methods for details). The RSEM-EVAL score is divided by 10^9. The *Number of ambiguous bases* is given in million. *Ex90N50* values are computed as usual N50 but limited to the top most highly expressed transcripts that represent 90 % of the total normalized expression data. An *F1* score of 1 states that all nucleotides/contigs in the estimated true assembly were recovered with at least 90 % identity. *KC score* – *k*-mer compression score reflecting the similarity of each assembly to DETONATES estimated "true" assembly. *Complete BUSCOs* – sum of single-copy and duplicated BUSCOs. Details and much more statistics complementing this evaluation can be found in the Electronic Supplement, Fig. S4–Tab. S9. Summaries for all other data sets can be found in Tab. S10.

		Trinity	Oases	Trans-ABySS	SOAP-Trans	Bridger	BinPacker	IDBA-Tran	Shannon	SPAdes-sc	SPAdes-rna
	k-mer size	default	25,35,45,55,65	25,35,45,55,65	default	default	default	25,35,45,55,65	default	default	default
**Evaluation metrics 1–20**											
HISAT2											
1	Overall mapping rate	$0.81_{91.9}$	$0.69_{88.04}$	$1.00_{98.34}$	$0.75_{89.93}$	$0.66_{86.83}$	$0.24_{72.6}$	$0.00_{64.61}$	$0.58_{84.27}$	$0.81_{92.04}$	$0.93_{95.95}$
rnaQUAST											
2	Transcripts ≥1000 nt	0.22_{64061}	1.00_{207474}	0.20_{59779}	0.03_{27529}	0.11_{43201}	0.00_{22611}	0.00_{23516}	0.05_{31328}	0.05_{31039}	0.15_{49860}
3	Misassemblies	0.99_{3378}	0.00_{216127}	0.99_{2743}	1.00_{279}	0.97_{7329}	0.98_{5603}	1.00_{302}	0.99_{2837}	0.99_{2022}	0.98_{5126}
4	Mismatches per transcript	$0.74_{1.38}$	$0.77_{1.25}$	$0.93_{0.57}$	$1.00_{0.27}$	$0.73_{1.44}$	$0.00_{4.63}$	$0.91_{0.67}$	$0.77_{1.26}$	$0.88_{0.8}$	$0.78_{1.25}$
5	Average alignment length	$0.27_{795.23}$	$0.06_{343.48}$	$0.01_{246.85}$	0.00_{218}	$0.21_{654.41}$	$1.00_{2335.73}$	$0.13_{487.11}$	$0.23_{711.83}$	$0.09_{410.22}$	$0.09_{412.24}$
6	95 %-assembled isoforms	0.99_{6788}	0.10_{868}	1.00_{6824}	0.31_{2264}	0.28_{2105}	0.39_{2824}	0.07_{709}	0.00_{242}	0.23_{1755}	0.46_{3253}
7	Duplication ratio	$0.00_{2.396}$	$0.03_{2.355}$	$0.47_{1.743}$	$0.87_{1.187}$	$0.50_{1.708}$	$0.01_{2.389}$	$1.00_{1.012}$	$0.63_{1.53}$	$1.00_{1.015}$	$0.87_{1.192}$
8	Ex90N50	0.00_{326}	0.17_{666}	0.06_{441}	0.19_{711}	0.51_{1370}	1.00_{2381}	0.19_{708}	0.49_{1324}	0.42_{1186}	0.22_{782}
9	# full-length transcripts	0.97_{8930}	0.83_{8024}	1.00_{9110}	0.64_{6806}	0.89_{8440}	0.26_{4456}	0.00_{2783}	0.63_{6758}	0.46_{5676}	0.69_{7155}
TransRate											
10	Reference coverage	$0.87_{0.23}$	$0.33_{0.09}$	$1.00_{0.26}$	$0.34_{0.09}$	$0.31_{0.09}$	$0.27_{0.07}$	$0.31_{0.08}$	0.00_{0}	$0.30_{0.08}$	$0.42_{0.11}$
11	Mean ORF percentage	$0.64_{50.82}$	$0.00_{42.09}$	$0.72_{51.92}$	$0.44_{48.02}$	$0.22_{45.1}$	$0.04_{42.57}$	$0.76_{52.46}$	$1.00_{55.7}$	$0.30_{46.13}$	$0.31_{46.25}$
12	Optimal score[a]	$0.30_{0.13}$	$0.00_{0.02}$	$0.23_{0.11}$	$0.66_{0.27}$	$0.32_{0.14}$	$0.14_{0.07}$	$0.61_{0.25}$	$0.13_{0.07}$	$1.00_{0.4}$	$0.57_{0.23}$
13	Percentage bases uncovered[a]	$0.38_{0.59}$	$0.00_{0.94}$	$0.33_{0.63}$	$0.67_{0.33}$	$0.57_{0.42}$	$0.11_{0.84}$	$1.00_{0.02}$	$0.48_{0.5}$	$0.99_{0.03}$	$0.79_{0.21}$
14	Number of ambiguous bases	0.72_{286}	0.00_{843}	0.53_{437}	0.78_{241}	0.83_{206}	1.00_{72}	0.91_{138}	0.94_{117}	0.86_{177}	0.71_{294}
DETONATE											
15	Nucleotide F1	$0.59_{0.43}$	$0.08_{0.18}$	$0.77_{0.51}$	$0.89_{0.57}$	$0.71_{0.48}$	$0.00_{0.15}$	$0.86_{0.55}$	$0.42_{0.35}$	$0.97_{0.61}$	$1.00_{0.62}$
16	Contig F1	$0.08_{0.02}$	$0.09_{0.02}$	$0.99_{0.2}$	$1.00_{0.21}$	$0.05_{0.01}$	0.00_{0}	$0.08_{0.02}$	$0.11_{0.02}$	$0.07_{0.01}$	$0.06_{0.01}$
17	KC score	$0.87_{0.51}$	$0.00_{0.24}$	$1.00_{0.55}$	$0.42_{0.37}$	$0.51_{0.4}$	$0.40_{0.37}$	$0.14_{0.29}$	$0.58_{0.42}$	$0.47_{0.39}$	$0.60_{0.43}$
18	RSEM EVAL	$0.98_{-6.51}$	$0.45_{-11.82}$	$1.00_{-6.26}$	$0.72_{-9.03}$	$0.85_{-7.72}$	$0.62_{-10.03}$	$0.00_{-16.3}$	$0.73_{-8.96}$	$0.42_{-12.12}$	$0.91_{-7.16}$
BUSCO											
19	Complete BUSCOs	0.96_{4004}	0.79_{3588}	1.00_{4106}	0.39_{2625}	0.92_{3909}	0.13_{2009}	0.00_{1682}	0.70_{3385}	0.39_{2625}	0.58_{3089}
20	Missing BUSCOs	0.99_{1804}	0.93_{1922}	1.00_{1770}	0.83_{2164}	0.98_{1812}	0.00_{4078}	0.63_{2615}	0.84_{2133}	0.78_{2268}	0.92_{1949}
**Summarized metric (0,1)-score**		12.38	6.31	14.24	11.92	11.13	6.59	8.61	10.3	11.47	12.03

[a]Not available for the *E. coli* and *A. thaliana* data set because this metric is only calculated by TransRate in the case of paired-end data.

Table 3. Overview of the different *de novo* assembly tools evaluated in this study. We rated our experiences regarding the installation and usability of each tool (😄 – excellent, 🙂 – good, ☹ – unsatisfactory). These experiences might be subjective, nevertheless we want to share them to give non-experienced users an idea of how difficult it is to get each tool installed (*Setup*) and executed (*Usage*), see Methods for details. For `Trinity`, we observed high memory peaks at the beginning of the calculations for large (human, mouse) data sets, which immediately returned to moderate memory levels after a few minutes. More details about runtime and memory consumption can be found in Electronic Supplement Fig. S11. MK – Whether or not the tool has a built-in multiple *k*-mer approach and is able to automatically integrate the output of different *k*-mer runs.

Assembler	Version	MK	Setup	Usage	Runtime			Memory (GB)			Ref.	Year
					min	max	median	min	max	median		
`Trans-ABySS`	2.0.1	yes	🙂	🙂	16 m	2 d 6 h 23 m	11 h 11 m	0.6	49.2	19.7	[9]	2010
`Trinity`	2.8.4	no	🙂	😄	28 m	1 d 20 h 10 m	6 h 40 m	7.2	243.9	27.7	[10]	2011
`Oases`[a]	0.2.08	yes	🙂	🙂	25 m	8 d 15 h 45 m	6 h 47 m	3.1	110.2	31.3	[11]	2012
`SPAdes-sc`[b]	3.13.0	yes	😄	😄	16 m	7 h 52 m	2 h 26 m	5.0	37.4	25.3	[18]	2012
`SPAdes-rna`[b]	3.13.0	yes[c]	😄	😄	11 m	7 h 24 m	2 h 17 m	5.0	44.2	19.5	[17]	2018
`IDBA-Tran`	1.1.1	yes	🙂	🙂	7 m	8 h 49 m	2 h 44 m	0.6	29.1	9.6	[12]	2013
`SOAPdenovo-Trans`	1.03	no	🙂	☹	1 m	1 h 48 m	24 m	2.1	45.6	26.4	[13]	2014
`Bridger`[d]	14-12-01	no	🙂	☹	11 m	21 h 11 m	5 h 9 m	1.6	109.3	30.4	[14]	2015
`BinPacker`[d]	1.0	no	🙂	😄	5 m	15 h 57 m	3 h 3 m	1.5	96.2	27.9	[15]	2016
`Shannon`	0.0.2	no	☹	🙂	9 m	10 h 45 m	3 h 18 m	3.8	121.4	83.6	[16]	2016

[a] `Oases` was used on top of the *de novo* genome assembler `Velvet` (v1.2.10) [45].
[b] `SPAdes`, originally designed as a *de novo* genome assembler for single-cell data, was used in single-cell modus (`-sc`) and RNA-Seq modus (`-rna`).
[c] When running `SPAdes` in RNA-Seq modus, two *k*-mer values are used by default.
[d] `Bridger` and `BinPacker` are based on a splicing graph construction instead of *de Bruijn* graphs.

length transcripts (Tab. S10). Based on these observations, we suggest that the RNA mode of `SPAdes` should be preferred for the reconstruction of larger eukaryotic RNA-Seq data sets.

Our comparisons with an older version of `SPAdes` running in RNA mode (at that time only one *k*-mer was allowed) revealed, that the performance of the algorithm was greatly improved by using two *k*-mers as it is now implemented in the current version [17].

Trans-ABySS.
Compared to the other tools, `Trans-ABySS` [9] achieved the highest re-mapping rates (98.45 % for *C. albicans*, 99.56 % for the simulated data; Fig. S4), however arranges only within the midfield or worse regarding the *optimal score* calculated by `TransRate`. On the other hand, the assemblies produced by `Trans-ABySS` achieved for six out of the nine data sets the best RSEM-EVAL scores. Only `Trinity` slightly outperformed `Trans-ABySS` regarding this metric for three data sets (Tab. S9). Therefore, the transcripts constructed by `Trans-ABySS` are well supported by the reads, used to build the assembly. `Trans-ABySS` performed good in all `BUSCO` analyses and showed a high amount of complete (C) ortholog detections (Fig. 3, Fig. S8). Many hits occur multiple times (complete and duplicated), for example in the *C. albicans* assembly (Fig. S8). This might be a result of the multiple *k*-mer approach (MK), when too many potential isoforms are assembled and not merged accurately at the end of the assembly process. In accordance with this, the assemblies of `Trans-ABySS` generally showed a high *duplication rate* (Fig. S5). We observed similar results for the MK runs of `Oases` [11]. Regarding the amount of fragmented (F) and missing (M) BUSCOs, `Trans-ABySS` arranges among the best performing tools (Fig. 3). `Trans-ABySS` achieved one of the highest *OMS* of 94.8 of all assembly tools (Fig. 2) and performed best for the large (human, mouse) data sets and the simulated data of human chromosome 1. By far, `Trans-ABySS` achieved the best *MS* (14.24) for the non-infected human data set. The lowest metric score was achieved for the bacterium data set (Fig. 2). Apart from the running time (Tab. 3), these results make `Trans-ABySS` one of the best-performing assembly tools in our comparison (besides `Trinity` and `SPAdes`).

Bridger.

In general, `Bridger` [14] assemblies resulted in high re-mapping rates between 87.35 % (*E. coli*) and 96.72 % (*C. albicans*, Fig. S4). For almost all `TransRate` metrics, the `Bridger` assemblies arrange in the midfield of scores (Tab. S6). According to the RSEM-EVAL scores, `Bridger` is performing generally well among the top tools (Tab. S9). Furthermore, `Bridger` performed well in the detection of complete BUSCOs with a moderate amount of duplicated hits. The amount of missing BUSCOs is comparably low (Fig. 3, Fig. S8). Based on a low *duplication ratio* and a low number of contigs, `Bridger` seems to produce very compact but also complete assemblies, especially for smaller data sets. The rate of mismatches per transcript is generally low (Tab. S10). Altogether, `Bridger` assemblies are of good quality and arrange among the top scores (*OMS*=89.3).

SOAPdenovo-Trans.

The re-mapping rate of `SOAPdenovo-Trans` [13] was generally high (>85 %), except for the *E. coli* data set (Fig. S4). `SOAPdenovo-Trans` performed quite well regarding most `TransRate` statistics and the calculated *optimal score* (Tab. S6). In most of the cases, only the `Trinity` and `SPAdes` assemblies could outperform `SOAPdenovo-Trans` regarding the `TransRate` metrics. The RSEM-EVAL scores vary depending on the assembled RNA-Seq data set (Tab. S9). For the HSA-EBOV-23h and *M. musculus* sample, `SOAPdenovo-Trans` achieved good RSEM-EVAL scores, whereas for the bacterial, the fungal, the plant and the simulated RNA-Seq data the tool places among the last three assemblers. The amount of *complete and duplicated BUSCOs* is very low (Fig. 3), which correlates with the generally low amount of detected isoforms (e.g. compare number of *95%-assembled isoforms* calculated with `rnaQUAST`, Fig. S5). This could be a result of the single *k*-mer approach. `SOAPdenovo-Trans` achieved a good *OMS* of 87.3 (Fig. 2) and the assembler performed well on each evaluated data set (*MS* between 10.28–15.05). `SOAPdenovo-Trans` was the only assembly tool capable of reconstructing the entire Ebola genome in a single contig from all three virus-infected data sets.

Shannon.
The most variant re-mapping rates were observed for Shannon [16], ranging between 30.77 % for the human simulated data set and 96.51 % for *A. thaliana* (Fig. S4). The Shannon assemblies did not result in good TransRate *optimal scores*, however the *percentage of uncovered bases* is placed in the midfield of all scorings and Shannon does not introduce that many *ambiguous bases* in the assembled transcriptome (Tab. S6). The RSEM-EVAL scores of Shannon vary among the assembled data sets (Tab. S9). Regarding the amount of assembled *complete BUSCOs*, Shannon arranges in the midfield and showed a relatively high amount of duplicated hits (Fig. 3). Shannon achieved a moderate *OMS* of 74.8 (Fig. 2).

IDBA-Tran.
In general, IDBA-Tran [12] achieved low re-mapping rates between 34.31 % (*E. coli*) and 89.04 % (*A. thaliana*), Fig. S4. However, the TransRate metrics of the IDBA-Tran assemblies are generally good (Tab. S6). Comparable to SOAPdenovo-Trans, some of the IDBA-Tran results arrange within the top three assemblies regarding the *optimal score* calculated by TransRate. DETONATES RSEM-EVAL scores reveal a different picture, as IDBA-Tran is in many cases placed last regarding this metric and never reaches the top five (Tab. S9). Furthermore, IDBA-Tran is one of the tools with the lowest amount of *complete BUSCOs* and a high amount of *fragmented and missing BUSCOs* (Fig. 3 and Fig. S8). The number of *95 %-assembled isoforms* is generally low (Tab. S10). IDBA-Tran is placed in the lower half of all metric scores (*OMS*=73.3, Fig. 2) and showed the best performances for smaller RNA-Seq data sets.

Oases.
The re-mapping rates of Oases [11] were generally good (>85 %). However, they dropped for the simulated human data (73.26 %), the HSA-EBOV-23h data (70.05 %) and the *E. coli* data (49.16 %) below acceptable thresholds (Fig. S4). Oases introduced the highest amount of *ambiguous bases* in the assemblies and arranges among the last places regarding the TransRate statistics (Tab. S6). Oases assemblies place in the last third regarding the RSEM-EVAL scores calculated by DETONATE. However, a good amount of *complete BUSCOs* could be detected, but many duplicate hits are included, which could be again a result of the MK approach (Fig. 3). In addition, the Oases assemblies comprise an enormous number of contigs (as well as high *duplication rates*) and introduce many *misassemblies* (Fig. S5). Oases performed best for the plant, bacteria, and simulated data and achieved an *OMS* of only 62.6 (Fig. 2).

BinPacker.
The re-mapping rates of BinPacker [15] were generally low and vary a lot between data sets (36.6–96.7 %, Fig. S4). The TransRate metrics of the BinPacker assemblies are comparable to the Bridger results, placing BinPacker among the lower-performing tools regarding this statistic (Tab. S6). On the other hand, BinPacker introduced only a low amount of *ambiguous bases* in the assemblies. The RSEM-EVAL score is comparatively low, except for the human simulated data, where BinPacker achieved a scoring similar to Bridger and reaches the third place behind Trinity and Trans-ABySS (Tab. S9). Regarding the detection of orthologs, BinPacker had the lowest performance of all tools and was only able to assemble a reasonable amount of *complete BUSCOs* for *C. albicans*, HSA-EBOV-7h and the human simulated data set (Fig. 3 and Fig. S8). BinPacker built the smallest assemblies in terms of the number of contigs (Fig. S5). Interestingly, BinPacker achieved for most data sets (and especially for the large human data sets) the best *Ex90N50* values (Tab. S7). Therefore, it seems that BinPacker can construct highly expressed transcripts into long contigs very well.

However, the general statistics and for example the BUSCO results show, that BinPacker misses a lot of transcripts that might be of low expression in the data sets. Overall, the performance of BinPacker is quite low (*OMS*=54.1, Fig. 2) and surprisingly far away from the performance of Bridger (*OMS*=89.3), although the assembler is build on the same principles and as an extension of Bridger [15]. In summary, BinPacker showed quite different behavior in relation to the *MS* values, which were generally low between 5.1 (*M. musculus*) and 12.24 (*C. albicans*), Fig. 2.

When designing this study, we also aimed to include an assembly tool that is not based on *k*-mers. Mira [46] (v4.0rc5) uses an overlap-consensus-graph for assembly and can be executed in EST mode for RNA-Seq data. However, for one human sample 62 h runtime were needed, >300 GB temporary files were produced and ~130 GB RAM consumed. Furthermore, we were not able to detect any BUSCO hits in the Mira assemblies. Due to this low performance and high running time and memory consumption, we decided to remove the tool from our comparison.

Usability

We rated our experiences regarding the installation and usability of each tool (Tab. 3). These experiences may be subjective, but we want to share them to give inexperienced users an idea of how difficult it is to install and run each tool. Some of the tools rely on many dependencies and/or are more difficult to compile (Shannon, SOAPdenovo-Trans, Trans-ABySS), at least on our test system without administrative permissions, while others could be installed easily (SPAdes). Furthermore, some assemblers need additional parameter files for execution (SOAPdenovo-Trans), are circuitous to run (Oases, SOAPdenovo-Trans), need additional preprocessing steps of the reads (IDBA-Tran assumes paired-end reads to be in order *forward–reverse*), or are just not terminating for all data sets (Bridger), while with others we had no problems and could execute them straightforward (Trinity, SPAdes, BinPacker).

Bridger failed in the path search step for some of the generated temporary files. Therefore we performed the last step of Bridger by manually combining the transcript output. Furthermore, we had to start Bridger two times for each data set, because the tool crashed each time after the first start, but continued with the assembly when started a second time on the same output folder (see execution commands in Files S3).

In the past, Oases and Trans-ABySS were always circuitous to run, because the corresponding genome assemblers Velvet [45] and ABySS [47] needed to be executed first with multiple *k*-mers. These difficulties were somehow emasculated by new wrapper scripts provided by the developers to automatically execute the underlying genome assemblers.

Computational efficiency

Since *de novo* transcriptome assembly can involve the analysis of large sequencing data, computational efficiency is an important benchmark, especially for deep sequencing projects and large sample sizes. Furthermore, it is highly recommended to run multiple assemblies with different tools and parameter settings (for example different *k*-mers), so computation time is an important part of each tool. Tab. 3 summarizes the computational time and the memory consumption of all data sets and assemblers. Details can be found in Electronic Supplement Fig. S11.

Runtime. By far, SOAPdenovo-Trans proved to be the fastest algorithm with a median runtime of only 24 m, followed by

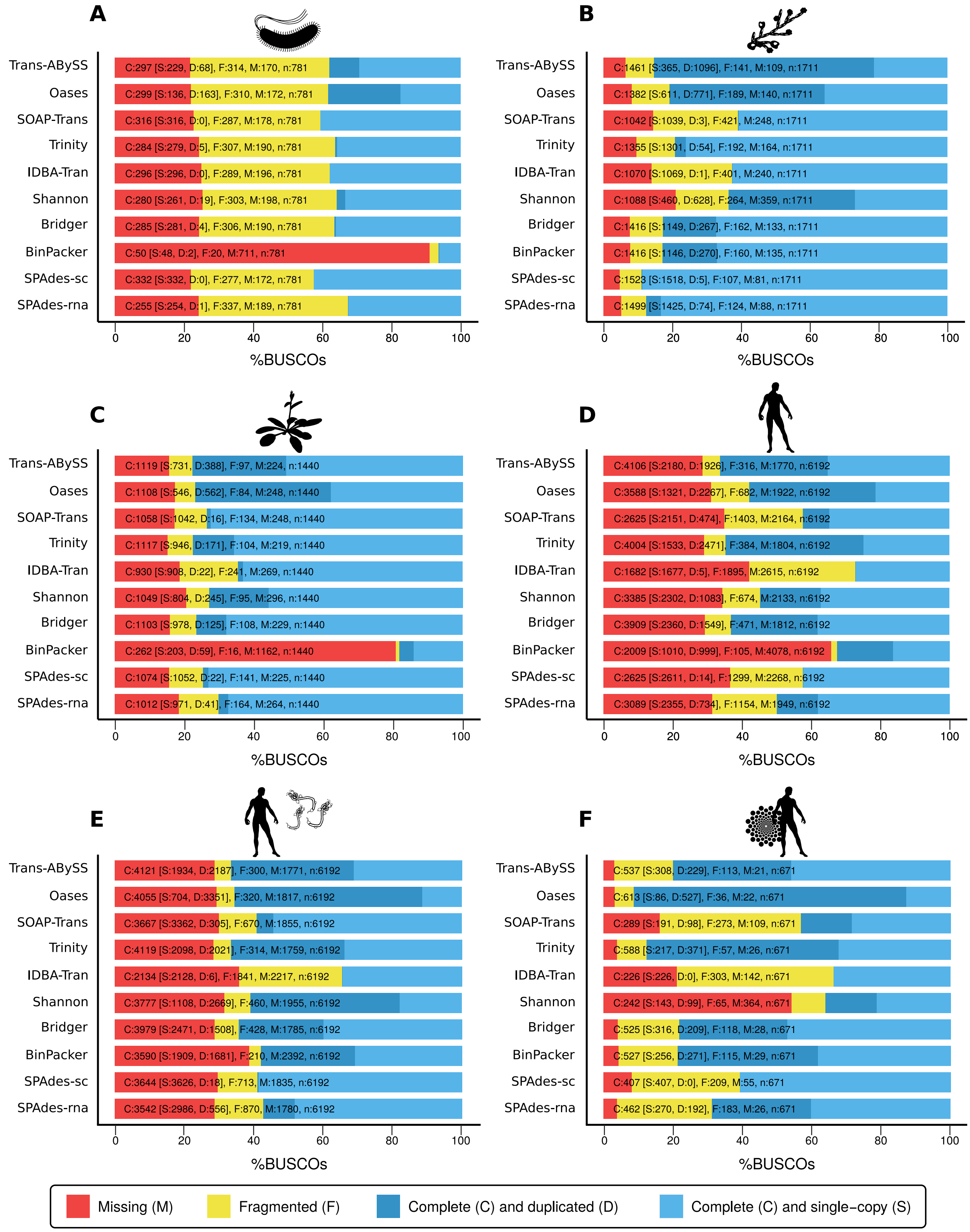

Figure 3. Selected BUSCO (*benchmarked universal single-copy orthologs*) [43, 44] assessment results for *E. coli* **(A)**, *C. albicans* **(B)**, *A. thaliana* **(C)**, *H. sapiens* **(D)**, HuH7 cells infected with EBOV 7 h post infection **(E)** and `flux` simulated reads [29] of human chromosome 1 **(F)**. The numbers indicate the absolute amount of complete (C) and single-copy (S), complete and duplicated (D), fragmented (F), and missing (M) BUSCOs (see Methods for details). For our evaluation, we have used the number of missing BUSCOs and the combined number of complete/single-copy and complete/duplicated BUSCOs to better consider alternative transcripts. BUSCO results for all other data sets can be found in the Electronic Supplement, Fig. S8.

SPAdes-rna (2 h 17 m), SPAdes-sc (2 h 26 m), IDBA-Tran (2 h 44 m), BinPacker (3 h 3 m), and Shannon (3 h 18 m) (Tab. 3, Fig. S11). Older tools such as Oases (6 h 47 m) and Trans-ABySS (11 h 11 m), that are additionally based on a multiple *k*-mer strategy (MK), are comparatively slower. For example, Oases needed more than 8 days for the large human RNA-Seq data set. If these tools would be executed only on one *k*-mer, the runtime would be comparable with the other assemblers or even faster. SOAPdenovo-Trans can also run on different *k*-mers, but no automatic merge function for the different assemblies is implemented. The Trinity runtime (6 h 40 m) lays between the faster tools and the slower MK approaches, although the tool relies on one *k*-mer only. Although based on a MK strategy, IDBA-Tran and SPAdes are much faster than the older MK-algorithms and can compete against the other single-*k*-mer tools in the sense of speed.

Memory consumption. IDBA-Tran appeared to be the tool with less memory consumption estimated over all data sets (median 9.6 GB and maximum 29.1 GB, Tab. 3, Fig. S11). Shannon showed really high memory peaks (median 83.6 GB), especially for the larger data sets (more than 100 GB for the EBOV-infected human samples, see Fig. S11), followed by Oases (31.3 GB), Bridger (30.4 GB) and BinPacker (27.9 GB).

When running Trinity (median memory 27.7 GB), we observed in the first phase of assembly (meaning in the first seconds up too few minutes, depending on the size of the input data set) very high memory peaks, especially for the larger data sets. For example, in the first five minutes of execution of all human data sets we noticed memory peaks of ∼240 GB with Trinity. Immediately after this initial peak, the memory consumption dropped down to comparatively normal levels (Fig. S11). In the Electronic Supplement figures S11, we removed the high initial memory peaks observed for Trinity from the comparison to achieve a better overview of the memory usage of all assemblers. The high memory consumption in the first phase might be a result of the many individual *de Bruijn* graphs build by Trinity based on partitions of the sequence data [10].

Users should pay particular attention to planning enough processing power and time when using many tools for different parameter settings, especially when working on projects with high sequencing depth and large sample size.

Contamination of viruses drops performance of most assembly tools

Although not the main focus of this study, we were interested in how the assemblers work with RNA-Seq data as virus contamination increases, and whether they are still able to construct complete viral genomes. Therefore, we used Blastn [48] to search for contigs in the virus-infected assemblies (Fig. 1) that match the full genome of the Ebola virus (EBOV). The EBOV genome comprises a single-stranded RNA genome with negative orientation and a size of approximately 19 kb [49]. We assembled three human samples infected with EBOV at three different time points. Therefore, we were able to investigate how the different assemblers perform on increasing amounts of viral reads in the data (3 h: ∼0.1 % viral reads, 7 h: ∼2 %, 23 h: ∼20 %; compare [33]).

Surprisingly, the performance of most assembly tools in constructing the viral RNA genome decreased with a higher amount of viral reads. In general, Trans-ABySS, SOAPdenovo-Trans, Trinity, Shannon, Bridger, BinPacker, and SPAdes (--sc and --rna mode) performed well and constructed the full EBOV genome out of the 3 h data set. On the 7 h data set (∼2 % viral reads), Trinity and SOAPdenovoTrans performed best. Trans-ABySS assembled two contigs (9.2 kb and 9.7 kb) that together would represent the entire EBOV genome. Bridger and BinPacker were only able to construct the same 10 kb partial EBOV genome. SPAdes-rna assembled a partial viral contig of a length of 16 kb. After 23 h post infection and a viral read contamination of almost ∼20 %, only SOAPdenovo-Trans was able to construct the full EBOV genome with a high accuracy (18,901 nt, 99.53 %). Bridger, BinPacker, and Trans-ABySS constructed partial virus genomes of a length of 14.8 kb, 12 kb, and 10.6 kb, respectively. Trinity built two contigs of similar length that together would cover the entire viral genome.

Discussion

Although the evaluation of *de novo* transcriptome assemblies was frequently performed in the past [6, 19–24, 26], there is still a lack of knowledge which assembler should be used for which kind of RNA-Seq data. Furthermore, these studies rely on limited data sets (e.g. a single species, a single sequencing protocol) or focus only on a subset of all currently available assembly tools. Here, we present a comprehensive evaluation of ten *de novo* assembly tools across various RNA-Seq data sets of different kingdoms of life.

Using a combination of biological-based and reference-free metrics to evaluate an assembly

We evaluated biological/reference-based metrics and statistical/reference-free metrics only based on the input read data and the final assembly itself. Evaluation metrics are very important to assess the quality of a genome or transcriptome assembly. However, there is a lack of consensus which evaluation metrics work best for *de novo* transcriptome assembly.

For example, Rana *et al.* [50] compared different assemblers and *k*-mer strategies using killifish RNA-Seq data and based their comparisons on eleven selected metrics, such as contig number, N50 value, contigs >1 kb, re-mapping rate, number of full-length transcripts, number of open reading frames, DETONATEs RSEM-EVAL score and the percentage of alignments to closely related fish. Another study performed comparisons on peanut RNA-Seq data and evaluated the assemblies on metrics such as N50, average contig length, number of contigs and the number of full-length transcripts [51]. Moreton *et al.* also used the N50 length, the number of transcripts, the number of transcripts ≥1 kb and RMBT and CEGMA percentages when evaluating different assemblies of duck [52]. Surely, more information on which metrics best predict the quality of a *de novo* transcriptome assembly would help to establish "best practice" protocols that could be further utilized to develop automatic evaluations to improve assemblies.

There is still a general lack of which metrics should be used for an appropriate evaluation of *de novo* transcriptome assemblies. More complicating, we observed that some metrics are contradicting each other, such as the *optimal assembly score* calculated by TransRate [42] and the RSEM-EVAL score of DETONATE [41]. For example, assemblies of the *Homo sapiens* simulated data set achieved the best RSEM-EVAL scores for Trans-ABySS and Trinity, whereas Shannon and IDBA-Tran performed worst (Tab. S9 and S10). However, IDBA-Tran achieved the second-best *optimal score* of TransRate only outperformed by SPAdes-sc and Shannon arranges on the next-to-last place regarding this metric (Tab. S6 and S10). On the other hand, certain metrics can be highly correlated (Figs. S12) and therefore lead to further distortions in assembly evaluation.

We conclude, that a careful selection of biological-based

and reference-free evaluation metrics is necessary to select the best performing results out of multiple assembly runs. In addition, the normalization and the way the results of different metrics are summarized can have a high influence on the evaluation. Based on our observations, we suggest to initially use reference-free metrics as provided by the TransRate [42] software. In general, TransRate's *optimal assembly score* seems to be a good measure of the quality of an assembly. Assemblies, that needed fewer contigs for a comprehensive description of the whole transcriptome achieved in most cases also good TransRate scores (Tab. S6). However, this score can be only calculated for paired-end RNA-Seq data at the moment.

If biological/reference-based metrics should be included, the *95%-assembled isoforms* statistics calculated by rnaQUAST [39] as well as the scores calculated by BUSCO [43, 44] and the *number of fully reconstructed protein-coding transcripts* are good metrics for the evaluation of the best assembly results.

Different species and RNA-Seq setups require specialized assembly tools

Although no tool performed dominantly best for all data sets, we found that Trinity [10], SPAdes [17, 18] and Trans-ABySS [9] produced consistently good assemblies among all data sets, followed by Bridger [14] and SOAPdenovo-Trans [13] (Fig. 2).

SPAdes, although originally developed as *de novo* assembly tool for small genomes, produced also highly accurate transcriptome assemblies in both modes, for single-cell (SPAdes-sc) and RNA-Seq data (SPAdes-rna). Interestingly, the single-cell mode outperformed the RNA mode for some of the data sets regarding our metrics (Fig. 2). This might be a result of the two *k*-mer approach and the different handling of single-end data in the RNA mode. According to the authors [17], SPAdes-rna was initially designed based on the principles of SPAdes-sc, so a multiple *k*-mer option could be easily activated as well. However, it was noticed that smaller *k*-mers result in a higher number of false junctions and lead to more misassemblies for transcriptomic data. Therefore, the authors decided to only use two *k*-mers as the default in RNA mode [17]. Furthermore, to join sequences with small overlaps, SPAdes-rna uses a gap-closing procedure based on read-pairs [17]. Indeed, this might be one reason why SPAdes-rna achieved for some metrics lower scores for single-end data. Taking a closer look at the BUSCO results, SPAdes produced in both modes the lowest amount of complete and duplicated transcripts (Fig. 3). This could further indicate that SPAdes merges highly similar transcripts into single contigs, therefore losing similar isoforms. This behavior can be also observed when looking at the number of *95%-assembled isoforms* calculated with rnaQUAST (Fig. S5 and Tab. S10). Here, the single-cell mode of SPAdes arranges for most data sets in the midfield whereas in RNA mode more complete isoform assemblies are constructed.

On closer examination of the BUSCO (Fig. 3) and *fully-reconstructed transcript* results, Oases [11] performed well overall. However, the tool produced the highest quantities of complete and duplicated hits, which might indicate that highly similar isoforms derived from the multiple *k*-mer approach are not resolved efficiently. Oases, as well as Trans-ABySS and SOAPdenovo-Trans, are constructing large assemblies with a high number of (sometimes very small) contigs. By far, Oases constructed the highest amount of contigs, however did not achieve the best reference coverage in all test cases. For example the Oases assembly of the *H. sapiens* data set comprises ~207,000 transcripts with a length >1000 nt, covering only 8 % of the reference transcripts (Tab. 2). In comparison, the Trans-ABySS assembly needed only ~59,000 contigs with a length >1000 nt to achieve a reference coverage of 26 % (Tab. S10). Therefore, Oases has the potential to create good assembly results, but also produces big assemblies with many contigs that might complicate and confuse downstream analyses.

With an average runtime of only 24 minutes over all data sets (maximum runtime 1 h 48 min), SOAPdenovo-Trans [13] outperformed all other assemblers (Tab. 3, Fig. S11). Combined with the moderate memory consumption (median 26.4 GB, maximum 45.6 GB), this makes SOAPdenovo-Trans the most resource-efficient tool evaluated in this study. However, it might be interesting to run multiple *k*-mer assemblies with SOAPdenovo-Trans and use another assembly merge strategy (e.g., conducted from Oases or TransABySS) to merge the final transcripts resulting from each run. In general, multiple *k*-mer approaches (Trans-ABySS, SPAdes, IDBA-Tran, Oases) performed better than single *k*-mer approaches regarding full-length isoform reconstruction and assembly completeness.

As long as the amount of viral contamination in RNA-Seq data is low (~0.1 %), all assembly tools except Oases and IDBA-Tran generated accurate viral contigs with high similarity to the EBOV genome and a length >18 kb. In general, SOAPdenovoTrans performed best on all three virus infected data sets by constructing accurate full-length contigs with high similarity to the EBOV genome. Therefore, it is interesting to evaluate the performance of SOAPdenovo-Trans for the construction of RNA viral genomes out of meta-transcriptomic RNA-Seq data in the future.

Potential implications

Here, we present a large-scale comparative study by applying ten *de novo* assembly tools to nine RNA-Seq data sets comprising different kingdoms of life (Fig. 1). Overall, we calculated more than 200 single assemblies and evaluated their performance on different metrics (Tab. 4). All results are summarized in a comprehensive Electronic Supplement, that is easily extendible by more RNA-Seq data sets, new assembler versions, parameter settings and tools. We summarize some key findings from our comparative study:

(I) No tool performed dominantly best for all data sets. However, Trinity, SPAdes and Trans-ABySS, followed by Bridger and SOAPdenovo-Trans, were among the best assembly tools (Fig. 2).

(II) SOAPdenovo-Trans followed by Trinity performed best for the construction of the Ebola virus single-stranded RNA genome at all three time points tested.

(III) SOAPdenovo-Trans had the lowest runtime, followed by SPAdes, IDBA-Tran, Shannon and BinPacker.

(IV) For assembly evaluation, we recommend a hybrid-approach by combining biological-based (e.g. BUSCO [43, 44], the *number of full-lenght transcripts*) and reference-free metrics (e.g. TransRate [42], DETONATE [41]).

In general, assembly tools such as Trinity, SPAdes and Trans-ABySS, which are still well maintained, outperformed other tools and should be preferred.

Some of our metrics might not provide independent assessment metrics, such as the number of *Complete BUSCOs* and the number of *full-length transcripts* (see Figs. S12). To account for such bias between highly correlated metrics, each of our (0,1)-normalized scoring vectors (see Methods) could be multiplied with a weight value (e.g. 0.5). Since it is a somewhat arbitrary decision on how to set the weight value for each metric and because we have also observed differences between the data sets, we have decided to not adjust weights in this comparison.

However, we only chose one of several metrics if our results suggested a strong correlation. In addition, our overall results do not appear to be strongly influenced by such metric correlations on the basis of our internal comparisons. Future assembly evaluation tools may allow the user to define weights for specific metrics or could calculate different weights automatically based on other statistics. Another possibility could be to bundle potentially correlated metrics on the basis of very similar normalized evaluation vectors.

Furthermore, our current comparison does not accurately test how well the individual assemblers reconstruct alternatively spliced transcripts. Therefore, another metric could be included to consider the assembler's ability to reconstruct different isoforms as an important aspect of a comprehensive evaluation of transcriptome assemblies.

Limitations and future work

We still recommend applying different tools and parameter settings for *de novo* transcriptome assembly, followed by the evaluation of the output transcripts and selecting the best-performing results. This general idea needs to be investigated in more detail in future studies. The selection of the best assemblies based on appropriate metrics and the subsequent clustering process (without loss of isoforms and the additional introduction of greater redundancy) remain challenging and open tasks.

Dynamic extension of this comparison.
A common problem of many comparative studies is that they can only make limited proposals based on the tools and data sets available at the time they were conducted. The Electronic Supplement provided here remains consistent with the presented results, but can be extended with other metrics, data sets and assembly tools in future updates.

Cluster assembly.
Furthermore, the complementary performance of the top performing tools motivated the development of an ensemble method by combining the best performing methods to achieve an overall better assembly. Based on our findings, a pipeline should be developed, that automatically selects the top performing assemblies (or only the best transcripts from each assembly) using a hybrid approach of biological-based and reference-free metrics and clusters them based on sequence-similarity and read-coverage to achieve a more comprehensive assembly.

For the large bioinformatics community working in the area of RNA-Seq, the development of a high-performing (accurate and fast) *de novo* transcriptome cluster workflow to automatically select and combine the output of top-performing assembly tools remains a challenging however crucial task.

Methods

Description of assembly tools and executed commands

We collected ten *de novo* assembly tools for the transcriptome reconstruction of the nine RNA-Seq data sets (Tab. 1), summarized in Tab. 3 and Electronic Supplement Tab. S2.

Six of these transcriptome assemblers are specially designed for working with RNA-Seq data and are based on *de Bruijn* graphs: `Trans-ABySS` (RRID:SCR_013322) [9], `Trinity` (RRID:SCR_013048) [10], `Oases` (RRID:SCR_011896) [11], `IDBA-Tran` (RRID:SCR_011891) [12], `SOAPdenovo-Trans` (RRID:SCR_013268) [13], and `Shannon` [16].

`Trans-ABySS` and `Oases` are built on top of the *de novo* genome assemblers `ABySS` v2.1.1 (RRID:SCR_010709) [47] and `Velvet` v1.2.10 (RRID:SCR_010755) [45], respectively. Both support multiple *k*-mer values by running the underlying genome assembler multiple times and merging the assembled contigs. We executed `Trans-ABySS` (v2.0.1) and `Oases` (v0.2.08) with multiple *k*-mers (MK) and in strand-specific mode, if suitable (Files S3).

`Trinity` and `SOAPdenovo-Trans` (the later one build on the principles of `SOAPdenovo2` (RRID:SCR_014986) [53]) are stand-alone *de novo* transcriptome assembly tools, also based on *de Bruijn* graphs but lacking an automated MK support. Whereas for `SOAPdenovo-Trans` different single *k*-mer values can be applied, `Trinity` relies on a fixed *k*-mer value of 25. `Trinity` (v2.8.4) was run with default parameters and, if suitable, in strand-specific mode (Files S3). For `SOAPdenovo-Trans` (v1.03), currently no strand-specific assembly is supported [13].

`IDBA-Tran` (v1.1.1), a novel assembly tool that claims to be more robust regarding uneven expression levels in RNA-Seq data [12], was run with multiple *k*-mers and has no option for strand-specific assembly (Files S3). `IDBA-Tran` assumes paired-end reads to be in order (->, <-; *forward-reverse*), therefore we manually converted reads if necessary.

`Shannon` (v0.0.2), a so-called information-optimal *de novo* RNA-Seq assembler [16], was used with a single default *k*-mer value and if suitable in strand-specific mode (`--ss`; Files S3).

We used `Bridger` [14] (v2014-12-01) and `BinPacker` [15] (v1.0), two assembly tools that rely on *splicing graphs* [14] instead of *de Bruijn* graphs. `Bridger` provides a new framework for *de novo* transcriptome assembly, that "bridges" between techniques employed in the `Cufflinks` [54] pipeline and the `Trinity` tool, in order to overcome the limitations of `Trinity`. `BinPacker` was developed based on the principles of `Bridger` and utilizes similar to `Shannon` coverage information to efficiently dissolve corresponding isoforms. `Bridger` can only run with single *k*-mer values between 19 and 32 with a default of 25. We executed `Bridger` with the default *k*-mer and, if possible, with the strand-specific option (`--SS_lib_type`). However, for two strand-specific RNA-Seq data sets (*M. musculus*, *H. sapiens*) the tool failed and was executed in the default unstranded mode (Files S3). We observed problems with strand-specific paired-end data in this version of `Bridger`. The strand-specific assembly of the single-end *E. coli* data (`--SS_lib_type F`) was running without problems. `BinPacker` was executed on a single *k*-mer value and if suitable in strand-specific (`-m F|RF`) mode (Files S3).

We further included `SPAdes` v3.13.0 (RRID:SCR_000131) [18], a widely used *de novo* genome assembler based on *de Bruijn* graphs and multiple *k*-mer values. We were interested, how good the tools optimization for single-cell assembly can be applied to RNA-Seq data and how the tool performs in contrast to the specialized transcriptome assemblers mentioned above. Since version 3.9.0 an RNA-Seq mode is implemented, which uses two *k*-mers for assembly so far [17]. We evaluated the performance of `SPAdes` in single-cell (`--sc`; `SPAdes-sc`) and

RNA-Seq (--rna; SPAdes-rna) mode. Henceforth, we refer to SPAdes-sc and SPAdes-rna as two different assemblers, although both are based on the same tool.

In total, we calculated more than 200 single *k*-mer assemblies (Files S3; doi.org/10.17605/OSF.IO/5ZDX4). Each assembler was run on each data set (Fig. 1). If possible, multiple *k*-mers were used (Tab. 3). Trans-ABySS, Oases and IDBA-Tran dispose a built-in functionality for multiple *k*-mers. SPAdes-sc/-rna can automatically choose multiple/two *k*-mers for the assembly process and were therefore executed with this default options. For the *E. coli*, *A. thaliana*, *H. sapiens*, and the artificial data sets *k*-mers 25, 35, 45, 55 and 65 were used with Trans-ABySS, Oases, and IDBA-Tran. *M. musculus* data was assembled with the *k*-mers: 25, 35, 45 and 55, because the read length is shorter in comparison to the bacterial and plant data sets. The short-read *C. albicans* data was run with *k*-mers 21, 27, 33 and 39. The EBOV infected HuH7 samples were run with *k*-mers 25, 29, 33, 37 and 41. All *k*-mer values were selected based on previous results for these data sets and in relation to the different read lengths and sequencing setups. All assemblers were run with default parameters, if not otherwise stated. Details about the execution of each tool on each data set can be found in the Electronic Supplement, Files S3.

Evaluation metrics

We benchmarked all assembly results using various evaluation tools (Fig. 1) from which 20 metrics were selected (summarized in Tab. 4). Nine metrics are based on reference sequences and annotations, whereas the others are only based on the final assembly itself (the contigs) or the reads that were used to construct the assembly. We also evaluated the computational efficiency (runtime, memory) to assess the applicability of the tools for deeply sequenced data sets and/or large sample size.

Mapping rate.

We have used HISAT2 v2.0.4 (RRID:SCR_015530) [40] to map the quality controlled reads back to each assembly. The remapping rate can give first insights into the quality of a transcriptome assembly (Fig. S4), however further metrics are needed to assess a more complete picture of each assembler's performance.

Ex90N50.

We have used the Trinity [10] toolkit utilities to calculate a modification of the widely used Nx statistic that also takes transcript expression data into account. This so-called expression-informed ExN50 statistic compensates for short and weakly expressed transcripts that can dominate a transcriptome assembly and can drive the N50 value towards small values for high-quality assemblies. Here we refer to the Ex90N50 value, which calculates the N50 statistics as usual, but is limited to the most highly expressed transcripts, which account for 90 % of the total normalized expression data. We used Salmon [55] (v0.11.3) for fast alignment-free abundance estimation to calculate the Ex90N50 values (Tab. S7).

Reconstruction of full-length protein-coding transcripts.

To assess the number of (nearly) full-length reconstructed protein-coding transcripts, we used Blastx (RRID:SCR_001653) [48] against the UniProtKB/Swiss-Prot database (RRID:SCR_004426) [56] followed by scripts provided by the Trinity [10] toolkit utilities. To improve the overall sequence coverage, we first grouped Blast hits of a single transcript aligning to a single protein sequence with several discontinuous alignments for each assembly (Trinity toolkit script: blast_outfmt6_group_segments.pl). Based on the grouped output, we have calculated the distribution of the percentage length coverage for the top matching database entries (blast_outfmt6_group_segments.tophit_coverage.pl). Finally, for each assembly the number of proteins that are covered by more than 90 % of their proteins length by assembled transcripts were reported.

Please note, that we performed the Blastx search with the parameters -evalue 1e-20 and -max_target_seqs 1. By setting the maximum target sequences to 1, we drastically reduced the runtime but only reported the first hit passing the e-value threshold. Therefore, we not necessarily report the best match for each transcript. This problem of misinterpretation of the parameter was recently discussed in the bioinformatics community [57]. However, for our comparison the overall results would only change slightly by increasing the maximum number of target sequences.

Table 4. Selected evaluation metrics applied for each assembly and data set. Metrics highlighted in gray are biological/reference-based. All other metrics only rely on the reads used to build the assembly and/or the resulting contigs. Details can be found in the Methods.

Nr.	Tool	Selected metric	Ref.
1	HISAT2	Overall mapping rate	[40]
2	rnaQUAST	Transcripts ≥1,000 nt	[39]
3		Misassemblies	
4		Mismatches per transcript	
5		Average alignment length	
6		95%-assembled isoforms	
7		Duplication ratio	
8	Trinity/Salmon	Ex90N50[a]	[10, 55]
9	Trinity/Blastx	Full-length transcripts[b]	[10, 48]
10	TransRate	Reference coverage	[42]
11		Mean ORF percentage	
12		Optimal score[c]	
13		Percentage bases uncovered[c]	
14		Number of ambiguous bases	
15	DETONATE	Nucleotide F1	[41]
16		Contig F1	
17		KC score	
18		RSEM-EVAL	
19	BUSCO	Complete BUSCOs[d]	[43, 44]
20		Missing BUSCOs	

[a] N50 statistic limited to the most highly expressed transcripts, which account for 90 % of the total normalized expression data, calculated with the Trinity toolkit utilities.
[b] Number of proteins covered by more than 90 % by assembled transcripts.
[c] Not available for the *E. coli* and *A. thaliana* data set because only calculated by TransRate if paired-end data is available.
[d] Sum of *complete single-copy* and *complete duplicated* BUSCOs.

rnaQUAST.

We used rnaQUAST [39] (v1.5.1) to calculate various statistics for each assembly and to demonstrate the completeness and correctness levels of the assembled transcripts. The tool was run with reference transcriptomes to calculate the *sensitivity* and *specificity* of an assembly. In order to check for redundancy in the assemblies, we have included the *duplication ratio* from the sensitivity report as one metric. Furthermore, rnaQUAST calculates various bar plots and histograms to visualize basic statistics such as transcript lengths, mismatch rates and the number of transcript alignments per isoform. All plots and detailed statistics can be found in the Electronic Supplement, Fig. S5.

TransRate.
`TransRate` [42] (v1.0.3) examines an assembly and compares it to experimental evidence such as the reads the assembly was built on. One of our metrics relies on the optimal reference-free `TransRate` score that utilizes only the reads that were used to generate the assembly as an evidence (Tab. 4). Such a metric should be generally better to optimize the assembly process because the comparison to a reference will always penalize genuine biological novelty contained in the assembly. The score is produced for the whole assembly and for every single contig. Currently, the score can be only calculated for paired-end data. The score of an assembly is calculated as the geometric mean of all contig scores multiplied by the proportion of input reads that provide positive support for the assembly [42]. Thus, the score captures how confident one can be in what was assembled, as well as how complete the assembly is. The minimum possible score is 0.0, while 1.0 is the maximum score.

DETONATE.
We further used the `DETONATE` workflow: a pipeline for the "DE novo TranscriptOme rNa-seq Assembly with or without the Truth Evaluation" [41] (v1.11). We mainly focused on `DETONATEs` RSEM-EVAL score. This statistically based evaluation score utilizes multiple factors, such as the compactness of the assembly and its support from the RNA-Seq reads. Therefore, the RSEM-EVAL score can be used to evaluate assemblies even when the ground truth is unknown. Assemblies with higher RSEM-EVAL scores are considered better. `DETONATE` was run for all assemblies as recommended in the online vignette [58]. The main metrics calculated by `DETONATE` can be found in Electronic Supplement Tab. S9.

BUSCO.
We benchmarked universal single-copy orthologs with `BUSCO` v2.0 (RRID:SCR_015008) [43]. The tool detects orthologous candidate genes in the assemblies and assesses the presence and abundance of single-copy orthologs as an evaluation criteria. The so-called BUSCOs are selected from OrthoDB orthologous groups at major species radiations requiring orthologs to be present as single-copy genes in the vast majority (> 90 %) of available species. `BUSCO` provides a quantitative assessment of the completeness of an assembly in terms of expected gene content. The results are further simplified into categories of (i) complete and single-copy, (ii) complete and duplicated, (iii) fragmented, or (iv) missing BUSCOs. For our evaluation, we summed up the amount of complete/single-copy and complete/duplicated BUSCOs to also take into account the different isoforms reconstructed from the assembly tools.

For the evaluation of the simulated human data set, the Euarchontoglires reference data set was reduced to `BUSCO` orthologs originating only from human chromosome 1 (# 671 BUSCOs). The full `BUSCO` output for each data set can be found in the Electronic Supplement, Fig. S8.

Calculation of normalized evaluation scores

We investigated the performance of ten *de novo* assembly tools $a_k \in \{a_1, \ldots, a_{10}\}$ on nine RNA-Seq data sets $d_i \in \{d_1, \ldots, d_9\}$ using 20 pre-selected metrics $m_j \in \{m_1, \ldots, m_{20}\}$. For each combination of a data set d_i and a metric m_j we define a vector $\bar{v}^{i,j}$ of raw scores $r_k^{i,j}$ for each assembly tool a_k as

$$\bar{v}^{i,j} = (r_1^{i,j}, \ldots, r_{10}^{i,j})$$

Then, we normalized the values of the vector $\bar{v}^{i,j}$ to the interval (0,1) using

$$normalize(\bar{v}_k^{i,j}) = \frac{\bar{v}_k^{i,j} - min(\bar{v}^{i,j})}{max(\bar{v}^{i,j}) - min(\bar{v}^{i,j})} = n_k^{i,j}$$

and denoted the resulting vector of (0, 1)-normalized scores as

$$\bar{n}^{i,j} = (n_1^{i,j}, \ldots, n_{10}^{i,j})$$

For example, the following vector of raw scores results for the *E. coli* data set d_{eco}, the metric *overall mapping rate* m_{omr}, and the corresponding raw scores of all ten assembly tools:

$$\bar{v}^{eco,omr} = (77.0, 49.1, 95.7, 56.6, 87.4, 71.1, 34.3, 76.7, 88.0, 89.0)$$

In this case, the assembly tool a_3 achieved an overall mapping rate of 95.7. After (0,1)-normalization the vector results in:

$$\bar{n}^{eco,omr} = (0.7, 0.24, 1.0, 0.36, 0.86, 0.6, 0.0, 0.69, 0.88, 0.89)$$

This normalization of the raw metric values to the interval (0,1) yields the same results as a *z*-score transformation with additional (0,1)-normalization.

We define the *metric score MS* for an assembly tool a_k and a data set d_i as the sum of all (0, 1)-normalized scores $\bar{n}_k^{i,j}$ over all 20 pre-selected metrics m_j as

$$MS(d_i, a_k) = \sum_{j=1}^{20} \bar{n}_k^{i,j}$$

An $MS(d_i, a_k)$ of 14.62 would mean that the assembler a_k for data set d_i achieved a normalized and summarized score of 14.62 from a maximum possible score of 20 (the number of metrics; denoted as 14.62/20).

To get a more general overview of the performance of each assembler, we summed up the metric scores *MS* an assembler achieved for each data set d_i to calculate an *overall metric score* (*OMS*) for each assembler:

$$OMS(a_k) = \sum_{i=1}^{9} MS(d_i, a_k)$$

The three human RNA-Seq data sets treated with the Ebola virus 3, 7, and 23 h post infection [33] are based on the same sequencing parameters and comprise roughly the same amount of reads (Fig. 1 and Tab. S1). Due to this similarity, we decided to reduce the impact of systematic assembly errors when calculating the *OMS* for one assembly tool and used the mean of all three *MS* scores for these three data sets (Fig. 2). For example, `Trans-ABySS` [9] performed very well in constructing the human transcripts out of all three Ebola-infected data sets regarding the *MS* (14.35/20, 14.11/20, and 13.87/20), whereas `BinPacker` [15] did not (4.81/20, 9.17/20, and 7.55/20), see Fig. 2.

The maximum achievable metric score for the *E. coli* and *A. thaliana* data sets is 18 and not 20, because the *optimal score* and the *percentage of uncovered bases* are only calculated by `TransRate` [42] in the case of paired-end data. The calculated *metric scores* (*MS*) and *overall metric scores* (*OMS*) are summarized in Fig. 2.

Computational resources

All calculations were run on two symmetric multiprocessing servers with 14 TB storage (raid-5) and 48 CPU cores each, comprising four AMD Opteron 6238 CPUs and 512 GB RAM. Each assembly was executed on 48 threads.

Usability

We further aimed to install and run all tools without root rights on our test system (Debian GNU/Linux 8 (jessie) 64-bit). Of course, how easy a tool can be installed and executed heavily depends on the used machine, the server setup and how familiar the user is with the programing language the tool is based on. Nevertheless, it should be the goal of each public available piece of software to be as user-friendly as possible. Therefore, we collected our experiences during the installation and execution of each assembler to share our observations (Tab. 3).

Availability of supporting data and materials

This study is accompanied by a comprehensive Electronic Supplement publicly available at `www.rna.uni-jena.de/supplements/assembly` [38]. The electronic supplement will stay consistent with the results presented in this paper. Updates, including new assembly tools, versions, and data sets, will be marked and additionally linked on subpages online. In addition, we have uploaded all processed read data, assemblies, blast alignments, mapping files and the complete electronic supplement as an additional archive into the Open Science Framework under accession doi.org/10.17605/OSF.IO/5ZDX4 [59]. Additional intermediate and final result files for evaluation tools such as BUSCO and TransRate as well as other results are also archived in the GigaScience GigaDB respository [60].

Declarations

Competing interests

None declared.

Funding

This work has been founded by the German Research Foundation (DFG) projects: Collaborative Research Center/Transregio 124 – "Pathogenic fungi and their human host: Networks of Interaction", subproject B5; DFG SPP-1596 – "Ecology and species barriers in emerging viral diseases"; and CRC 1076 "AquaDiva", subproject A06.

Authors' contributions

MM conceived the research idea. MH designed the project, performed calculations and analysis, interpreted the data and wrote the main manuscript. MM contributed in discussions and in proofreading the final manuscript. This work is part of the doctoral thesis of MH. All authors read and approved the final manuscript.

Acknowledgements

We delightfully thank the team of the HPC-Cluster ARA, located at the University of Jena, that was very helpful in speeding up preliminary calculations and tests.

References

1. Wang Z, Gerstein M, Snyder M. RNA-Seq: a revolutionary tool for transcriptomics. Nat Rev Genet. 2009 Jan;10:57–63.
2. Martin JA, Wang Z. Next-generation transcriptome assembly. Nat Rev Genet. 2011 Sep;12:671–682.
3. Corney DC. RNA-Seq using Next Generation Sequencing. Mater Methods. 2013;3:203.
4. Conesa A, Madrigal P, Tarazona S, Gomez-Cabrero D, Cervera A, McPherson A, et al. A survey of best practices for RNA-seq data analysis. Genome Biol. 2016;17(1):13.
5. Hrdlickova R, Toloue M, Tian B. RNA-Seq methods for transcriptome analysis. Wiley Interdisciplinary Reviews: RNA. 2017;8(1):e1364.
6. Sahraeian SME, Mohiyuddin M, Sebra R, Tilgner H, Afshar PT, Au KF, et al. Gaining comprehensive biological insight into the transcriptome by performing a broad-spectrum RNA-Seq analysis. Nat Commun. 2017;8(1):59.
7. Haas BJ, Zody MC. Advancing RNA-Seq analysis. Nat Biotechnol. 2010 May;28:421–423.
8. Lima L, Sinaimeri B, Sacomoto G, Lopez-Maestre H, Marchet C, Miele V, et al. Playing hide and seek with repeats in local and global *de novo* transcriptome assembly of short RNA-Seq reads. AMB. 2017;12:2.
9. Robertson G, Schein J, Chiu R, Corbett R, Field M, Jackman SD, et al. *De novo* assembly and analysis of RNA-seq data. Nat Methods. 2010;7(11):909–912.
10. Grabherr MG, Haas BJ, Yassour M, Levin JZ, Thompson DA, Amit I, et al. Full-length transcriptome assembly from RNA-Seq data without a reference genome. Nat Biotechnol. 2011;29(7):644–652.
11. Schulz MH, Zerbino DR, Vingron M, Birney E. Oases: robust *de novo* RNA-Seq assembly across the dynamic range of expression levels. Bioinformatics. 2012 Apr;28:1086–1092.
12. Peng Y, Leung HCM, Yiu SM, Lv MJ, Zhu XG, Chin FYL. IDBA-tran: a more robust *de novo de Bruijn* graph assembler for transcriptomes with uneven expression levels. Bioinformatics. 2013 Jul;29:i326–i334.
13. Xie Y, Wu G, Tang J, Luo R, Patterson J, Liu S, et al. SOAPdenovo-Trans: *de novo* transcriptome assembly with short RNA-Seq reads. Bioinformatics. 2014 Jun;30:1660–1666.
14. Chang Z, Li G, Liu J, Zhang Y, Ashby C, Liu D, et al. Bridger: a new framework for *de novo* transcriptome assembly using RNA-seq data. Genome Biol. 2015;16:30.
15. Liu J, Li G, Chang Z, Yu T, Liu B, McMullen R, et al. BinPacker: Packing-Based *De Novo* Transcriptome Assembly from RNA-seq Data. PLOS Comput Biol. 2016;12:e1004772.
16. Kannan S, Hui J, Mazooji K, Pachter L, Tse D. Shannon: An Information-Optimal *de Novo* RNA-Seq Assembler. bioRxiv. 2016;p. 039230.
17. Bushmanova E, Antipov D, Lapidus A, Przhibelskiy AD. rnaSPAdes: a *de novo* transcriptome assembler and its application to RNA-Seq data. bioRxiv. 2018;p. 420208.
18. Bankevich A, Nurk S, Antipov D, Gurevich AA, Dvorkin M, Kulikov AS, et al. SPAdes: a new genome assembly algorithm and its applications to single-cell sequencing. J Comput Biol. 2012;19(5):455–477.
19. Kumar S, Blaxter ML. Comparing *de novo* assemblers for 454 transcriptome data. BMC Genom. 2010 Oct;11:571.
20. Chen G, Yin K, Wang C, Shi T. De novo transcriptome assembly of RNA-Seq reads with different strategies. Sci China Life Sci. 2011 Dec;54:1129–1133.
21. Zhao QY, Wang Y, Kong YM, Luo D, Li X, Hao P. Optimizing *de novo* transcriptome assembly from short-read RNA-Seq data: a comparative study. BMC Bioinf. 2011 Dec;12 Suppl 14:S2.

22. Lu B, Zeng Z, Shi T. Comparative study of *de novo* assembly and genome-guided assembly strategies for transcriptome reconstruction based on RNA-Seq. Sci China Life Sci. 2013 Feb;56:143–155.
23. Clarke K, Yang Y, Marsh R, Xie L, Zhang KK. Comparative analysis of *de novo* transcriptome assembly. Sci China Life Sci. 2013 Feb;56:156–162.
24. Wang S, Gribskov M. Comprehensive evaluation of *de novo* transcriptome assembly programs and their effects on differential gene expression analysis. Bioinformatics. 2017;33(3):327–333.
25. Geniza M, Jaiswal P. Tools for building *de novo* transcriptome assembly. Curr Plant Biol. 2017;11:41–45.
26. Voshall A, Moriyama EN. Next-Generation Transcriptome Assembly: Strategies and Performance Analaysis. In: Abdurakhmonov I, editor. Bioinformatics in the Eera of Post Genomics and Big Data. London: IntechOpen; 2018. p. 15–36.
27. Andrews S. FastQC: a quality control tool for high throughput sequence data.; 2010. Available online at: http://www.bioinformatics.babraham.ac.uk/projects/fastqc.
28. Schmieder R, Edwards R. Quality control and preprocessing of metagenomic datasets. Bioinformatics. 2011;27:863–864.
29. Griebel T, Zacher B, Ribeca P, Raineri E, Lacroix V, Guigó R, et al. Modelling and simulating generic RNA-Seq experiments with the flux simulator. Nucleic Acids Res. 2012;40:10073–10083.
30. Thomason MK, Bischler T, Eisenbart SK, Förstner KU, Zhang A, Herbig A, et al. Global transcriptional start site mapping using differential RNA sequencing reveals novel antisense RNAs in *Escherichia coli*. J Bacteriol. 2015 Jan;197:18–28.
31. Cottier F, Tan ASM, Chen J, Lum J, Zolezzi F, Poidinger M, et al. The transcriptional stress response of *Candida albicans* to weak organic acids. G3 (Bethesda). 2015 Jan;5:497–505.
32. Lai Z, Schluttenhofer CM, Bhide K, Shreve J, Thimmapuram J, Lee SY, et al. MED18 interaction with distinct transcription factors regulates multiple plant functions. Nat Commun. 2014;5:3064.
33. Hölzer M, Krähling V, Amman F, Barth E, Bernhart SH, Carmelo VAO, et al. Differential transcriptional responses to Ebola and Marburg virus infection in bat and human cells. Sci Rep. 2016;6:34589.
34. Flicek P, Amode MR, Barrell D, Beal K, Brent S, Carvalho-Silva D, et al. Ensembl 2012. Nucleic Acids Res. 2012 Jan;40:D84–D90.
35. Ensembl. Bacteria Collection.; 2018. Accessed 14 July 2018. ftp://ftp.ensemblgenomes.org/pub/bacteria/release-34/gtf/bacteria_0_collection/escherichia_coli_str_k_12_substr_mg1655/.
36. Ensembl. Plant Collection.; 2018. Accessed 14 December 2018. ftp://ftp.ensemblgenomes.org/pub/release-34/plants/fasta/arabidopsis_thaliana/dna/.
37. CGD. *Candida* Genome Database.; 2018. Accessed 14 December 2018. www.candidagenome.org.
38. Hölzer M. Electronic Supplement. *De novo* transcriptome assembly: A comprehensive cross-species comparison of short-read RNA-Seq assemblers.; 2018. Accessed 14 December 2018. http://www.rna.uni-jena.de/supplements/assembly.
39. Bushmanova E, Antipov D, Lapidus A, Suvorov V, Prjibelski AD. rnaQUAST: a quality assessment tool for *de novo* transcriptome assemblies. Bioinformatics. 2016;32:2210–2212.
40. Kim D, Langmead B, Salzberg SL. HISAT: a fast spliced aligner with low memory requirements. Nat Methods. 2015;12(4):357–360.
41. Li B, Fillmore N, Bai Y, Collins M, Thomson JA, Stewart R, et al. Evaluation of *de novo* transcriptome assemblies from RNA-Seq data. Genome Biol. 2014 Dec;15:553.
42. Smith-Unna R, Boursnell C, Patro R, Hibberd JM, Kelly S. TransRate: reference-free quality assessment of *de novo* transcriptome assemblies. Genome Res. 2016 Aug;26:1134–1144.
43. Simão FA, Waterhouse RM, Ioannidis P, Kriventseva EV, Zdobnov EM. BUSCO: assessing genome assembly and annotation completeness with single-copy orthologs. Bioinformatics. 2015 Oct;31:3210–3212.
44. Waterhouse RM, Seppey M, Simão FA, Manni M, Ioannidis P, Klioutchnikov G, et al. BUSCO applications from quality assessments to gene prediction and phylogenomics. Mol Biol Evol. 2017;35(3):543–548.
45. Zerbino DR, Birney E. Velvet: algorithms for *de novo* short read assembly using *de Bruijn* graphs. Genome Res. 2008 May;18:821–829.
46. Chevreux B, Pfisterer T, Drescher B, Driesel AJ, Müller WE, Wetter T, et al. Using the miraEST assembler for reliable and automated mRNA transcript assembly and SNP detection in sequenced ESTs. Genome Res. 2004;14(6):1147–1159.
47. Simpson JT, Wong K, Jackman SD, Schein JE, Jones SJM, Birol I. ABySS: a parallel assembler for short read sequence data. Genome Res. 2009 Jun;19:1117–1123.
48. Altschul SF, Gish W, Miller W, Myers EW, Lipman DJ. Basic local alignment search tool. J Mol Biol. 1990;215(3):403–410.
49. Feldmann H, Klenk HD, Sanchez A. Molecular biology and evolution of filoviruses. Arch Virol Suppl. 1993;7:81–100.
50. Rana SB, Zadlock FJ, Zhang Z, Murphy WR, Bentivegna CS. Comparison of *De Novo* Transcriptome Assemblers and k-mer Strategies Using the Killifish, *Fundulus heteroclitus*. PLOS One. 2016;11:e0153104.
51. Chopra R, Burow G, Farmer A, Mudge J, Simpson CE, Burow MD. Comparisons of *de novo* transcriptome assemblers in diploid and polyploid species using peanut (Arachis spp.) RNA-Seq data. PLOS One. 2014;9:e115055.
52. Moreton J, Dunham SP, Emes RD. A consensus approach to vertebrate *de novo* transcriptome assembly from RNA-seq data: assembly of the duck (*Anas platyrhynchos*) transcriptome. Front Genet. 2014;5:190.
53. Luo R, Liu B, Xie Y, Li Z, Huang W, Yuan J, et al. SOAPdenovo2: an empirically improved memory-efficient short-read *de novo* assembler. GigaScience. 2012 Dec;1:18.
54. Trapnell C, Williams BA, Pertea G, Mortazavi A, Kwan G, van Baren MJ, et al. Transcript assembly and quantification by RNA-Seq reveals unannotated transcripts and isoform switching during cell differentiation. Nat Biotechnol. 2010 May;28:511–515.
55. Patro R, Duggal G, Love MI, Irizarry RA, Kingsford C. Salmon provides fast and bias-aware quantification of transcript expression. Nat Methods. 2017;14(4):417.
56. Consortium U. UniProt: a hub for protein information. Nucleic Acids Res. 2014;43(D1):D204–D212.
57. Shah N, Nute MG, Warnow T, Pop M. Misunderstood parameter of NCBI BLAST impacts the correctness of bioinformatics workflows. Bioinformatics. 2018;.
58. Li B. Vignette for DETONATE.; 2018. Accessed 14 December 2018. http://deweylab.biostat.wisc.edu/detonate/vignette.html.
59. Hölzer M, Marz M. Supplement: Transcriptome assembly comparison (Open Science Framework); 2019. https://doi.org/10.17605/OSF.IO/5ZDX4.
60. Hölzer M, Marz M. Supporting data for "*De novo* transcriptome assembly: A comprehensive cross-species comparison of short-read RNA-Seq assemblers"; 2019. Giga-

Science Database. http://dx.doi.org/10.5524/100576.
