# Supplementary material for: De novo transcriptome assembly: A comprehensive cross-species comparison of short-read RNA-Seq assemblers
Source: Gigascience. 2019 May 11;8(5):giz039. doi: 10.1093/gigascience/giz039 (PMC6511074; doi:10.1093/gigascience/giz039)
Supplement: Response_to_Reviewer_Comments_Original_Submission.pdf [file giz039_response_to_reviewer_comments_original_submission.pdf]

Author's Response To Reviewer Comments

Close

Dear Dr. Zhou,

please find attached the revised version of our manuscript entitled

“De novo transcriptome assembly: A comprehensive cross-species comparison of short-read RNA-Seq assemblers”

which was submitted as an original research article to GigaScience (GIGA-D-18-00307).

We thank the reviewers for the enthusiastic interest in our study and the very insightful and thoughtful comments.

We have addressed each point of criticism of the reviewers as outlined in the attached point-by-point response.

As suggested, we re-run new versions of assembly tools and re-calculated all evaluations and statistics. We
updated all figures, the text, and our Electronic Supplement (http://www.rna.uni-jena.de/supplements/assembly/) accordingly.
Furthermore, we introduced a more robust scoring scheme using scores normalized between 0 and 1. Next to our Electronic Supplement, all data
(processed read files, all assemblies, . . .) are now also publicly available at the Open Science Framework under
accesion: https://doi.org/10.17605/OSF.IO/5ZDX4. In our eyes, updating the assembly tool versions and introducing a
new scoring scheme has significantly improved the study.

As we also included figures/tables in the point-to-point letter, we uploaded a PDF as additional file

hoelzer_p2p.pdf

Please pass our responses (hoelzer_p2p.pdf) together with our revised manuscript to the reviewers.

Again, we confirm that this work is original and has not been published elsewhere, nor is it currently considered
for publication elsewhere.

The manuscript in its current state has been seen and approved by all listed authors.

Thank you again for your consideration and please do not hesitate to contact me if there are any further questions.

With my best regards for the new year,
Martin Hoelzer

Close
