# Supplementary material for: De novo transcriptome assembly: A comprehensive cross-species comparison of short-read RNA-Seq assemblers
Source: Gigascience. 2019 May 11;8(5):giz039. doi: 10.1093/gigascience/giz039 (PMC6511074; doi:10.1093/gigascience/giz039)
Supplement: Response_to_Reviewer_Comments_Revision_1.pdf [file giz039_response_to_reviewer_comments_revision_1.pdf]

Author's Response To Reviewer Comments

Close

Dear Dr. Zhou,

please find attached the revised version of our manuscript entitled

“De novo transcriptome assembly: A comprehensive cross-species comparison of short-read RNA-Seq assemblers”

which was submitted as an original research article to GigaScience (GIGA-D-18-00307R1).

We are pleased that our study has now been considered potentially acceptable for publication in GigaScience. Again, we want to thank you and the both referees' for the great review process.

Regarding the one missing point mentioned by Reviewer #2:

My only final critique of the manuscript refers to page 10, left column, lines 60-61: "but failed on the 23h data set (many small contigs, longest hit: 10,677 nt).", which conflicts with what was written in the response to reviewers and in the main text at right column, lines 12-13: "Trinity built two contigs of similar length that together would cover the entire viral genome."

We totally agree and just removed this sentence. This was a remnant before we used the new version of Trinity and now with the additional sentence ("Trinity built two contigs...") we already make this point clear.

Accordingly, we submit now the manuscript like seen and approved by the two referees', just without this sentence.

The manuscript in its current state has been seen and approved by all listed authors.

Please do not hesitate to contact me if there are any further questions.

Yours Sincerely,
Martin Hoelzer

Close
