# Supplementary material for: De novo transcriptome assembly: A comprehensive cross-species comparison of short-read RNA-Seq assemblers
Source: Gigascience. 2019 May 11;8(5):giz039. doi: 10.1093/gigascience/giz039 (PMC6511074; doi:10.1093/gigascience/giz039)
Supplement: Response_to_Reviewer_Comments_Revision_2.pdf [file giz039_response_to_reviewer_comments_revision_2.pdf]

Author's Response To Reviewer Comments

Close

I included the following requested changes:

1) added a reference [59] for the OSF and cited it in the data availability section

2) added a reference [60] for GigaDB and wrote in the data availability section:
"Additional intermediate and final result files for evaluation tools such as BUSCO and TransRate as well as other results are also archived in the GigaScience GigaDB repository [60]."

3) added RRIDs for:
- Trans-ABySS (RRID:SCR_013322)
- Trinity (RRID:SCR_013048)
- Oases (RRID:SCR_011896)
- IDBA-Tran (RRID:SCR_011891)
- SOAPdenovo-Trans (RRID:SCR_013268)
- ABySS (RRID:SCR_010709)
- Velvet (RRID:SCR_010755)
- SOAPdenovo2 (RRID:SCR_014986)
- SPAdes (RRID:SCR_000131)
- HISAT2 (RRID:SCR_015530)
- BLASTX (RRID:SCR_001653)
- UniProtKB/Swiss-Prot (RRID:SCR_004426)
- BUSCO (RRID:SCR_015008)

Thank you very much and best wishes,
Martin Hoelzer

Close
