# Supplementary material for: De novo transcriptome assembly: A comprehensive cross-species comparison of short-read RNA-Seq assemblers
Source: Gigascience. 2019 May 11;8(5):giz039. doi: 10.1093/gigascience/giz039 (PMC6511074; doi:10.1093/gigascience/giz039)
Supplement: Reviewer_1_Report_Original_Submission -- Andrey D. Prjibelski, M.Sc. — 9/26/2018 Reviewed [file giz039_reviewer_1_report_original_submission.pdf]

Reviewer Report

Title: De novo transcriptome assembly: A comprehensive cross-species comparison of short-read RNA-Seq assemblers

Version: Original Submission **Date:** 9/26/2018

Reviewer name: Andrey D. Prjibelski, M.Sc.

Reviewer Comments to Author:

General comments
The described work is devoted to the comparison of different popular de novo transcriptome assemblers. For this purpose, the authors use various state-of-the-art tools and metrics to assess the assemblies on multiple datasets sequences from different types of organisms. The paper is well-structured, easy to read, has clear and informative figures and tables. Selected data sets seem to cover the majority of possible use cases for de novo RNA-Seq assemblers, performed analysis is clear, and conclusions made regarding different tools seem to be reasonable as well. In addition to quality evaluation, the authors also assess the assemblers performance and usability, which may often be important when choosing the assembly method. It worths noting, the authors provided all technical information, such as command lines, software versions, datasets and full quality evaluation tables in the supplementary material, which makes the research completely reproducible and also allows to independently analyze the obtained results.
Although similar studies were made regarding de novo genome assembly software (Salzberg et al., 2012; Magoc et al., 2013), no complete assessment of transcriptome assemblers was previously performed. Taking into account the popularity of projects that involve de novo RNA-Seq assembly nowadays, I, therefore, consider this work to be very useful for the bioinformatics community. However, this work contains rather technical, but very important issue, that I suggest to address before publication.
Major comments
The main concern is the software version of the assemblers being used. I clearly realize that this work most likely started quite a time ago and it may take significant time to run all the tools on all datasets. However, versions of the assemblers that are still being supported and have new releases are significantly outdated (more than 1,5 years). Moreover, the developers of these assemblers claim that significant improvements were introduced in the new releases (see below). Since the authors make the analysis and conclusions based on the old versions, the manuscript in its current form, unfortunately, does not provide the entirely updated information to the community, and therefore partially loses its key point.
Indeed, new results may change some conclusions. However, the good news are that only three assembler are still being improved:
- Trinity. New version: 2.8.4 (12.09.2018), used version: 2.3.2 (20.11.2016). New versions includes both algorithmic and technical improvements (see https://github.com/trinityrnaseq/trinityrnaseq/releases).
- rnaSPAdes. New version: 3.12.0 (14.05.2018), used version: (04.12.2016). New version includes various improvemetns in transcriptome assembly algorithms, multiple perfromance improvemetns and bugfixes

(see http://cab.spbu.ru/files/release3.12.0/changelog.html). Note, that BayesHammer error correction module is the same for SPAdes-sc and SPAdes-rna and did not change since 3.9.0. Reusing corrected reads may also save time.
- Trans-ABySS. New version: 2.8.4 (12.09.2018), used version: 2.3.2 (20.11.2016). New version does not contain any algorithmic improvemetns, only support for ABySS 2 (http://www.bcgsc.ca/platform/bioinfo/software/trans-abyss). Probably no need to rerun.

Minor comments and suggestions

Below I point out minor issues and advices that, in my opinion, may make this work even more complete in terms of assembly analysis.

1. I suggest to consider adding one of such metrics as the number of 95%-assembled genes/isoforms (rnaQUAST) or cov95 (TransRate reference based analysis) since they represent how well known genes/isoforms are actually restored in the assembly, which is an important property of the de novo assembler. To keep the reasonable number of metrics the authors may, for example, substitute "mean isoform coverage", as it does not really reflect assembly contiguity.
2. Another useful and representative metrics, which can be also included in the set of main metrics is "duplication ratio" from sensitivity rnaQUAST report. Although the authors mention similar metrics like Duplicated BUSCOs and number of ambiguous bases, in general there are no stats representing assembly redundancy.
3. As far as I know, although SPAdes-sc and IDBA-tran use several k-mers, in fact they not perform merging of several outputs in a common way, but rather do an iterative assembly (reusing contigs obtained with smaller k during next iteration). Thus, it probably makes sense to rephrase the sentence about merging functionality (page 5, left column, first paragraph), e.g. to remove notion "merge".
4. The authors mention high memory peaks for some tools. Adding peak RAM (or median peak) to the performance table may also be useful in addition to median RAM, since it also may show the limitations of using the software on smaller machines.
5. Is there any specific reason why there are two dots after "Performance" and "Memory consumption" paragraph titles (pages 8 and 10) or just typos?
6. Regarding comparison of SPAdes-sc and rnaSPAdes. rnaSPAdes was initially designed based on SPAdes-sc, so it is easy to enable multi k-mer option as well. However, we noticed that using small k-mer size may lead to higher number of false junctions and, therefore, misassemblies (1566 vs 570 misassemblies for SPAdes-sc and SPAdes-rna respectively in Table 2 and mostly similar ratio in supplementary tables). To perform more accurate assembly we decided to disable multiple k-mer option, but slightly lowered k-mer size being used as default (Bushmanova et al., 2018). We also suspect that Trinity also has rather high number of misassemblies possibly due to small k-mer size being used. To join sequences with small overlap rnaSPAdes uses some kind of gap closing procedure based on read-pairs (Bushmanova et al., 2018). Indeed, this is exactly the reason why rnaSPAdes has worse assemblies for single-end data.

These details are provided mainly for the information, and author are free to ignore them since rnaSPAdes preprint was released lately.

7. The authors suggest to use "mean isoform coverage" metric in the discussion section. Although this metric is informative, I suggest to highlight the number of 95%-assembled genes/isoforms (or "mean isoform assembly") and here is the main reason why. E.g. assembler A assembles the entire gene in a

single contig, while assembler B assembles the same gene in two contigs (each covering only a half). Mean isoform coverage for both will be 1.0, while mean isoform assembly will be 1.0 and 0.5 respectively. Thus, "mean isoform coverage" represents recall only at nucleotide level, but not at a gene level, and does not represent assembly contiguity. In addition, both mean isoform coverage and mean isoform assembly are highly affected by filtering contigs that represent poorly assembly genes â€” assembler that tries to restore all possible genes gets worse values that the one that report only long reliable contigs (although the number of 95%-assembled genes/isoforms can be the same).

The author are free to contact regarding any questions.

Sincerely yours

Andrey Prjibelski

References

Bushmanova, E., Antipov, D., Lapidus, A., & Prjibelski, A. D., 2018. rnaSPAdes: a de novo transcriptome assembler and its application to RNA-Seq data. bioRxiv, p.048942.

Magoc, T., Pabinger, S., Canzar, S., Liu, X., Su, Q., Puiu, D., Tallon, L.J. and Salzberg, S.L., 2013. GAGE-B: an evaluation of genome assemblers for bacterial organisms. Bioinformatics, 29(14), pp.1718-1725.

Salzberg, S.L., Phillippy, A.M., Zimin, A., Puiu, D., Magoc, T., Koren, S., Treangen, T.J., Schatz, M.C., Delcher, A.L., Roberts, M. and Marcais, G., 2012. GAGE: A critical evaluation of genome assemblies and assembly algorithms. Genome research, 22(3), pp.557-567.

Methods

Are the methods appropriate to the aims of the study, are they well described, and are necessary controls included? Choose an item.

Conclusions

Are the conclusions adequately supported by the data shown? Choose an item.

Reporting Standards

Does the manuscript adhere to the journal's guidelines on minimum standards of reporting? Choose an item.

Choose an item.

Statistics

Are you able to assess all statistics in the manuscript, including the appropriateness of statistical tests used? Choose an item.

Quality of Written English

Please indicate the quality of language in the manuscript: Choose an item.

Declaration of Competing Interests

Please complete a declaration of competing interests, considering the following questions:

- Have you in the past five years received reimbursements, fees, funding, or salary from an organisation that may in any way gain or lose financially from the publication of this manuscript, either now or in the future?
- Do you hold any stocks or shares in an organisation that may in any way gain or lose financially from the publication of this manuscript, either now or in the future?
- Do you hold or are you currently applying for any patents relating to the content of the manuscript?
- Have you received reimbursements, fees, funding, or salary from an organization that holds or has applied for patents relating to the content of the manuscript?
- Do you have any other financial competing interests?
- Do you have any non-financial competing interests in relation to this paper?

If you can answer no to all of the above, write 'I declare that I have no competing interests' below. If your reply is yes to any, please give details below.

I declare that I have no competing interests

I agree to the open peer review policy of the journal. I understand that my name will be included on my report to the authors and, if the manuscript is accepted for publication, my named report including any attachments I upload will be posted on the website along with the authors' responses. I agree for my report to be made available under an Open Access Creative Commons CC-BY license (http://creativecommons.org/licenses/by/4.0/). I understand that any comments which I do not wish to be included in my named report can be included as confidential comments to the editors, which will not be published.

I agree to the open peer review policy of the journal

To further support our reviewers, we have joined with Publons, where you can gain additional credit to further highlight your hard work (see: https://publons.com/journal/530/gigascience). On publication of this paper, your review will be automatically added to Publons, you can then choose whether or not to claim your Publons credit. I understand this statement.

Yes Choose an item.
