# Supplementary material for: De novo transcriptome assembly: A comprehensive cross-species comparison of short-read RNA-Seq assemblers
Source: Gigascience. 2019 May 11;8(5):giz039. doi: 10.1093/gigascience/giz039 (PMC6511074; doi:10.1093/gigascience/giz039)
Supplement: Reviewer_2_Report_Original_Submission -- Brian Haas — 9/27/2018 Reviewed [file giz039_reviewer_2_report_original_submission.pdf]

Reviewer Report

Title: De novo transcriptome assembly: A comprehensive cross-species comparison of short-read RNA-Seq assemblers

Version: Original Submission **Date:** 9/27/2018

Reviewer name: Brian Haas

Reviewer Comments to Author:

The work by Holzer and Marz aims to provide a comprehensive assessment of the performance of de novo transcriptome assemblers. The authors target the assembly of RNA-seq data sets spanning diverse organisms representing bacteria, plants, animal, and fungi, using 10 different transcriptome assemblers and characterizing their performance using 20 different performance metrics. While there have been previously published works to assess performance of transcriptome assemblies, the work presented here is more comprehensive than earlier studies, targeting data sets representing greater breadth of evolutionary diversity and including most all de novo transcriptome assembly tools currently available. The manuscript is well written and the analyses lend insights into the behavior of the different assembly methods on these data sets, in addition to providing an overall ranking of methods from least to highest performance. There are certain areas where I expect the work could be improved, which I detail below. I also add the disclaimer that I am Brian Haas, lead developer of the Trinity de novo transcriptome assembly software (one of the tools being assessed here), and some of my comments will reflect specific concerns that I have about Trinity's performance on certain data sets.

Assembler performance is characterized by a metric score (MS), defined as the count of times that a given assembler is found as one of the top 3 best tools according to a given metric: There are 20 metrics, and each metric can be ranked from lowest to highest on a leaderboard. If any assembler ends up in one of the top 3 positions of the leaderboard for that metric, it's MS is incremented by one. This choice was a clever way of scoring the tools, but is not entirely robust. The choice of top 3 for the leaderboard positions is somewhat arbitrary. Setting it to a different number instead of three will have a major effect on the relative rankings of the different tools. An alternative approach that may be more principled is to rank according to the mean ranking value across all metrics. One might also consider the distribution of rankings across the different data sets, as certain methods may be routinely outperforming others on certain metrics and performing very poorly in other metric categories, whereas others may be performing admirably (even if not best) across most if not all metrics explored.

Many of these metrics being used are not providing independent assessment metrics. If all metrics are indeed useful for assessing assembly quality, and some number of metrics are highly correlated, any method that performs well in that group of highly correlated metrics will have a great advantage in the scoring scheme. Ideally, highly correlated metrics would be weighted in assessment, or one might prune the list of metrics to avoid such bias. For example, the TransRate 'Optimal.score' and 'Percentage.good.mappings' are nearly perfectly correlated, as are the pair of metrics 'Reference.coverage' and 'Database.coverage'. Perhaps one or the other should be used. Note that, however, by accounting for this, the overall results presented in the manuscript do not appear to be

heavily influenced by metric correlation based on my own independent evaluation of their results.
I am generally skeptical about whether all metrics chosen do provide a useful measure of assembly quality. I'm convinced that metrics such as those from DETONATE (KC.score and RSEM.EVAL), BUSCO (Missing.BUSCOs), and 'Percentage.good.mappings' are excellent metrics for characterizing assembly quality. The N50 value is well known to be an imperfect metric for assembly transcriptome assembly quality. In Trinity, we advocate using an expression-informed ExN50 statistic instead of the N50 to compensate for short lowly expressed transcripts that can dominate a transcriptome assembly and drive the N50 values towards small values on high quality assemblies. The use of 'Complete.single.copy.BUSCOs' as a metric unfair for transcriptome assemblers that excel at modeling alternatively spliced transcripts. Instead, the sum of all complete BUSCO entries (single or duplicate) should be a better metric for transcriptome assembly evaluation. Also note that this study does not include a metric that addresses the quality of alternatively spliced transcript reconstruction, which would be an important aspect of any comprehensive transcriptome assembly assessment. Another metric that I personally favor is counting the number of fully reconstructed transcripts. This may not be fully independent from the BUSCO statistics, but is still a useful metric to include in any comprehensive assessment, just be sure to take into account highly correlated metrics when cumulatively scoring methods.

Now on to my Trinity concerns. Overall, I'm pleased to see that Trinity is well portrayed in this evaluation and is shown to perform well. From looking at the Detonate scores (RSEM.EVAL and KC.score) across metrics, in addition to the overall rankings, it's clear that something fairly catastrophic must have happened for the Trinity run on the HumanEboV3h data set. Here, Trinity is a clear outlier and strikingly poor compared to all other methods. This is very surprising. I'm curious to know if something unusual happened during that run. It would be worthwhile to try running Trinity on that one data set again to see if the poor performance there is reproducible. The other issue has to do with Trinity not assembling the full length Ebola virus in the infected samples. There was actually a bug in the earlier version of Trinity that hindered the assembly of certain very long transcripts in non-strand-specific RNA-Seq assemblies (strand-specific data was largely unaffected). This bug was fixed in later Trinity releases. I did download and assemble each of these three Ebola-infected samples from SRA and assembled each using Trinity v2.8.4 (current release). In each case, Trinity did fully reconstruct the 18kb Ebola virus, thankfully. I don't expect you to have to rerun all samples with the latest version of Trinity, but it is worth noting that the reason for the noted problematic behavior here has been addressed. This bug was brought to our attention by a Trinity user earlier this year. Since it only seemed to impact exceedingly long transcripts (outliers) with non-strand-specific data, this behavior wasn't noted in earlier routine evaluations of standard RNA-Seq data sets and was easily overlooked.

I appreciate that the authors made the table of metrics available through their website. I was able to download these data and reanalyze them as part of my review. I encourage the authors to include a full data table of metrics as supplementary data, in csv or tsv format. Also, it would be useful to have the individual assemblies and other key data, including commands executed for assembly and metric generation as part of the supplementary material to companion the published manuscript, as opposed to being available at some volatile website location. I expect these data will be useful for future work evaluating alternative metrics for assembly quality assessment and further comparative studies.

Methods

Are the methods appropriate to the aims of the study, are they well described, and are necessary controls included? Choose an item.

Conclusions

Are the conclusions adequately supported by the data shown? Choose an item.

Reporting Standards

Does the manuscript adhere to the journal's guidelines on [minimum standards of reporting?]() Choose an item.

Choose an item.

Statistics

Are you able to assess all statistics in the manuscript, including the appropriateness of statistical tests used? Choose an item.

Quality of Written English

Please indicate the quality of language in the manuscript: Choose an item.

Declaration of Competing Interests

Please complete a declaration of competing interests, considering the following questions:

- Have you in the past five years received reimbursements, fees, funding, or salary from an organisation that may in any way gain or lose financially from the publication of this manuscript, either now or in the future?
- Do you hold any stocks or shares in an organisation that may in any way gain or lose financially from the publication of this manuscript, either now or in the future?
- Do you hold or are you currently applying for any patents relating to the content of the manuscript?
- Have you received reimbursements, fees, funding, or salary from an organization that holds or has applied for patents relating to the content of the manuscript?
- Do you have any other financial competing interests?
- Do you have any non-financial competing interests in relation to this paper?

If you can answer no to all of the above, write 'I declare that I have no competing interests' below. If your reply is yes to any, please give details below.

1. No 2. No 3. No 4. No 5. I'm the lead developer of the Trinity software, one of the tools used in this manuscript. 6. No

I agree to the open peer review policy of the journal. I understand that my name will be included on my report to the authors and, if the manuscript is accepted for publication, my named report including any attachments I upload will be posted on the website along with the authors' responses. I agree for my report to be made available under an Open Access Creative Commons CC-BY license (http://creativecommons.org/licenses/by/4.0/). I understand that any comments which I do not wish to be included in my named report can be included as confidential comments to the editors, which will not be published.

I agree to the open peer review policy of the journal

To further support our reviewers, we have joined with Publons, where you can gain additional credit to further highlight your hard work (see: https://publons.com/journal/530/gigascience). On publication of this paper, your review will be automatically added to Publons, you can then choose whether or not to claim your Publons credit. I understand this statement.

Yes Choose an item.
