# Supplementary material for: De novo transcriptome assembly: A comprehensive cross-species comparison of short-read RNA-Seq assemblers
Source: Gigascience. 2019 May 11;8(5):giz039. doi: 10.1093/gigascience/giz039 (PMC6511074; doi:10.1093/gigascience/giz039)
Supplement: Reviewer_2_Report_Revision_1 -- Brian Haas — 1/27/2019 Reviewed [file giz039_reviewer_2_report_revision_1.pdf]

**Reviewer Report**

**Title: De novo transcriptome assembly: A comprehensive cross-species comparison of short-read RNA-Seq assemblers**

**Version: Revision 1** **Date:** 1/27/2019

**Reviewer name: Brian Haas**

**Reviewer Comments to Author:**

I appreciate the authors response to my and the other reviewer's comments, and I feel that the manuscript has been significantly improved as a result of the revisions. The revised scoring system used by the authors is far superior to the earlier method used. The adjustment to the metrics used to account for correlations along with including the additional metrics also added to the rigor of the work. I commend the authors for all their efforts.
My only final critique of the manuscript refers to page 10, left column, lines 60-61: "but failed on the 23h data set (many small contigs, longest hit: 10,677 nt).", which conflicts with what was written in the response to reviewers and in the main text at right column, lines 12-13: "Trinity built two contigs of similar length that together would cover the entire viral genome."

**Methods**

Are the methods appropriate to the aims of the study, are they well described, and are necessary controls included? Choose an item.

**Conclusions**

Are the conclusions adequately supported by the data shown? Choose an item.

**Reporting Standards**

Does the manuscript adhere to the journal's guidelines on [minimum standards of reporting?](minimum standards of reporting?) Choose an item.

Choose an item.

**Statistics**

Are you able to assess all statistics in the manuscript, including the appropriateness of statistical tests used? Choose an item.

**Quality of Written English**

Please indicate the quality of language in the manuscript: Choose an item.

**Declaration of Competing Interests**

Please complete a declaration of competing interests, considering the following questions:

- Have you in the past five years received reimbursements, fees, funding, or salary from an organisation that may in any way gain or lose financially from the publication of this manuscript, either now or in the future?
- Do you hold any stocks or shares in an organisation that may in any way gain or lose financially from the publication of this manuscript, either now or in the future?
- Do you hold or are you currently applying for any patents relating to the content of the manuscript?
- Have you received reimbursements, fees, funding, or salary from an organization that holds or has applied for patents relating to the content of the manuscript?
- Do you have any other financial competing interests?
- Do you have any non-financial competing interests in relation to this paper?

If you can answer no to all of the above, write 'I declare that I have no competing interests' below. If your reply is yes to any, please give details below.

I am currently the lead developer of the Trinity software which is one of the tools being assessed in this manuscript.

I agree to the open peer review policy of the journal. I understand that my name will be included on my report to the authors and, if the manuscript is accepted for publication, my named report including any attachments I upload will be posted on the website along with the authors' responses. I agree for my report to be made available under an Open Access Creative Commons CC-BY license (http://creativecommons.org/licenses/by/4.0/). I understand that any comments which I do not wish to be included in my named report can be included as confidential comments to the editors, which will not be published.

I agree to the open peer review policy of the journal

To further support our reviewers, we have joined with Publons, where you can gain additional credit to further highlight your hard work (see: https://publons.com/journal/530/gigascience). On publication of this paper, your review will be automatically added to Publons, you can then choose whether or not to claim your Publons credit. I understand this statement.

Yes Choose an item.
